# Effectiveness of acupuncture and moxibustion therapy on glycolipid metabolism in patients with obese-type polycystic ovarian syndrome: A systematic review and network meta-analysis

**DOI:** 10.1097/MD.0000000000042812

**Published:** 2025-06-13

**Authors:** Zhili Yu, Shumin Chen, Qiao Zhang, Wenbin Fu, Hongling Geng, Cong Wang, Hao Wen

**Affiliations:** a Department of Acupunctue-Moxibustion and Tuina, The Third Affiliated Hospital of Guangzhou University of Chinese Medicine, Guangzhou, China; b Department of Traditional Chinese Medicine, Wenquan Hospital of Guangdong Province Veteran Cadre Affairs Center, Guangzhou, China; c Medical College of Acupuncture-Moxibustion and Rehabilitation, Guangzhou University of Chinese Medicine, Guangzhou, China; d The Second Affiliated Hospital of Guangzhou University of Chinese Medicine, Guangzhou, China; e Department of Acupuncture, Guangzhou Huangpu District Hospital of Traditional Chinese Medicine, Guangzhou, China.

**Keywords:** acupuncture, glycolipid metabolism, moxibustion, network meta-analysis, obesity, polycystic ovarian syndrome

## Abstract

**Background:**

The efficacy of acupuncture and moxibustion-related therapy (AMRT) on the glycolipid metabolism in patients with polycystic ovarian syndrome (PCOS), especially those with an obese phenotype, is still uncertain. This paper aims to analyze the impact of AMRT on glycolipid metabolism in managing obese-type PCOS.

**Methods:**

This systematic review and meta-analysis synthesized evidence from randomized controlled trials on AMRT, compared to conventional western medicine or blank control for obese PCOS focusing on their glycolipid metabolism, searched across 7 databases. Direct and Indirect Meta-analysis was conducted using Review Manager 5.4 and R program, presenting continuous data as mean differences (MD) with 95% confidence intervals (CIs). The Cochrane risk-of-bias tool for trials 2 (ROB2) was used for methodological quality assessment, and the Grading of Recommendation Assessment, Development, and Evaluation (GRADE) approach evaluated the certainty of evidence.

**Results:**

Thirty randomized controlled trials were included. Compared to control treatments, AMRT significantly improved fasting plasma glucose (FPG) (MD = −0.19, 95% CI = −0.28 to −0.09, moderate certainty), Weight (W) (MD = −2.34, 95% CI = −4.51 to −0.17, moderate certainty) and triglycerides (TG) (MD = −0.35, 95% CI = −0.40 to −0.29, moderate certainty), and showed similar benefits for low-density lipoprotein (MD = −0.20, 95% CI = −0.40 to −0.01, moderate certainty) and total cholesterol (MD = −0.36, 95% CI = −0.49 to −0.23, moderate certainty). The other indicators were deemed unreliable or showed negative results. During network meta-analysis, Moxibustion combined with catgut embedding was the most effective measure in the improvement of FPG, fasting insulin (FIN) and insulin resistance (IR). Manual acupuncture combined with metformin ranked third improving BMI, TG and IR, while ranked first in waist-hip ratio (WHR). No statistical incoherence in all comparisons of direct with indirect evidence for efficacy and treatment discontinuation.

**Conclusion:**

Acupuncture and related therapies can effectively improve glycolipid metabolism (FPG, W, total cholesterol, TG, low-density lipoprotein) in obese PCOS patients (moderate certainty evidence) and have a comparable effect to conventional medicine on improving IR (moderate certainty evidence). Among them, moxibustion plus catgut embedding was the most optimal AMRT on reducing blood glucose, while manual acupuncture combined with metformin was the best AMRT applying on ameliorating blood lipids.

## 1. Introduction

Polycystic ovarian syndrome (PCOS) is recognized as one of the most prevalent reproductive endocrine metabolic disorders, known to cause hyperandrogenism, anovulation, and increased risk of type 2 diabetes mellitus (T2DM) and cardiovascular disease. Large-scale epidemiological studies have reported the prevalence of PCOS to vary from 2.2% to 11.2% in China and 4% to 13% in the UK and USA, depending on the selected study populations and the diagnostic criteria applied.^[1]^ In more focused studies, such as those involving health sciences students, hospital, and city-based populations, prevalence rates approximate 8%.^[2–5]^ Moreover, it is well-documented that individuals with PCOS are at an elevated risk of developing metabolic disorders and obesity.^[1]^ Notably, PCOS, as a metabolic disturbance, may affect both men and women.^[6,7]^ A study conducted at a specialist Endocrine Clinic in South Asia reported metabolic disorders in 30.6% of the PCOS group compared to 6.34% of controls, with 51.20% of women with PCOS having a BMI > 25 kg/m^2^.^[8]^ The incidence of both syndromes increases with age.^[9]^

International guidelines recommend various Western medicines for managing PCOS, which, while effective, are known to have certain side effects, such as nausea and vomiting. Non-pharmaceutical interventions, including acupuncture and moxibustion, have been widely accepted due to their minimal side effects and cost-effectiveness. Several meta-analyses have concluded that acupuncture can improve menstrual cycle disorders, glucose metabolism, and insulin sensitivity.^[10,11]^ Furthermore, acupuncture and moxibustion-related therapies (AMRT) as complementary and alternative medicine might enhance the therapeutic effects of Western medicines.^[12,13]^

However, most meta-analyses have focused on ovulation rate, pregnancy rate, and sex hormone levels,^[11–14]^ with fewer studies addressing glucolipid metabolism, and these limited to the direct comparison among simple acupuncture interventions.^[10,15]^ The effectiveness of both acupuncture and moxibustion in improving glycolipid metabolism in obese patients with PCOS has yet to be summarized comprehensively. Therefore, our study aims to use network meta-analysis to evaluate and rank the effectiveness of AMRT on glycolipid metabolism in obese patients with PCOS from direct or indirect evidence.

## 2. Methods

The protocol for this systematic review and meta-analysis, titled “Effectiveness of AMR for glycolipid metabolism in patients with polycystic ovarian syndrome: a systematic review and meta-analysis,” was registered with PROSPERO (CRD42023421584), and is reported according to the Preferred Reporting Items for Systematic Reviews and Meta-Analyses (PRISMA) guideline for network meta-analysis extension statement (see Supplementary Material S16, Supplemental Digital Content, https://links.lww.com/MD/P149, for PRISMA checklist).

### 2.1. Eligibility criteria

Studies were included if they: involved patients diagnosed with obesity-type PCOS, as defined by recognized clinical criteria (e.g., ESHRE/ASRM 2003; AE-PCOS 2006); included interventions of acupuncture or moxibustion; involved acupuncture needles inserted into the skin; used moxibustion with wormwood or its derivatives; combined acupuncture or moxibustion with other interventions; had control groups consisting of placebo control, waitlist control, no intervention or conventional Western medicine. Moreover, Western medicine can include contraceptives like Diane-35 to treat hyperandrogenism; ovulation induction drug like Clomiphene citrate or chorionic gonadotropin to assist reproductive; Metformin to adjust metabolic disorders, based on recommendations from the 2023 international evidence-based guideline for the assessment and management of PCOS. Reported outcomes included weight (W), body mass index (BMI), waist circumference (WC), waist-hip ratio (WHR), fasting blood glucose (FPG), fasting insulin (FIN), insulin resistance (IR), total cholesterol (TC), triglycerides (TG), low-density lipoprotein (LDL) cholesterol (LDL-C), or high-density lipoprotein (HDL) cholesterol (HDL-C), measured before and after treatment, with or without follow-up. Were randomized controlled trials (RCTs) published in English or Chinese. Studies were excluded if their control groups included special or rarely-used interventions not listed in internationally accepted guidelines, or they were conference papers or academic dissertations.

### 2.2. Search strategy

Literature searches were conducted across 7 databases: PubMed, Medline, Cochrane Library, ClinicalKey, Embase, China National Knowledge Infrastructure, and Wanfang Data, using a combination of Mesh terms, entry terms, and keywords related to Polycystic Ovary Syndrome, Acupuncture, Moxibustion, and RCTs. The search strategy’s full details are available in Supplementary Material S14, Supplemental Digital Content, https://links.lww.com/MD/P149, with no time limit up to April 26, 2024.

### 2.3. Study selection

Two authors, Zhili Yu (ZY) and Qiao Zhang (QZ), independently and in duplicate screened the titles and abstracts, excluding those obviously irrelevant to these reviews. Subsequently, the full texts of the studies were accessed and carefully reviewed based on the eligibility criteria. Any discrepancies were discussed among 3 authors (ZY, QZ, SC) until a consensus was reached.

### 2.4. Data extraction and synthesis

Data extracted from each study by 2 authors (ZY, QZ) included: title, first author’s name, publication year, type of intervention in each group, frequency of acupuncture or moxibustion, duration of acupuncture or moxibustion sessions, specific type of PCOS, and number of participants in each group. The primary outcome measures were indicators of glycolipid metabolism (W, BMI, WC, WHR, FPG, FIN, IR, TC, TG, LDL-C, HDL-C).

### 2.5. Risk-of-bias

The methodological quality of studies has been assessed by 2 independent authors in duplicate (ZY, QZ) using the Cochrane Collaboration’s tool including 5 domains of bias. Each domain was evaluated and classified into 3 grades, that are, high risk, some concerns and low risk. A final evaluation categorized the studies as high, some concerns or low quality. Any discrepancies will be discussed by 3 authors (ZY, QZ, CS) until an agreement can be reached.

## 3. Outcomes

The outcomes were the efficacy of the interventions at the end of treatment. The primary outcome was FPG and TG and the secondary outcome was BMI, WHR, FIN, TC, IR, HDL, LDL, W, and WC.

### 3.1. Evidence certainty assessment

We utilized the GRADEpro GDT software (GRADEpro GDT 2023) to assess evidence quality across 5 GRADE domains: risk-of-bias, inconsistency, indirectness, imprecision, and publication bias.^[16]^ When limitations were identified, evidence quality was downgraded accordingly.

### 3.2. Statistical analysis

To minimize the impact of baseline differences on outcomes, changes in outcomes between the pretreatment and posttreatment were extracted and analyzed. When articles provided only pre- and posttreatment values, a formula^[16]^ was used to calculate the changes in outcomes. If there were more than 2 intervention groups, a formula^[16]^ recommended by the Cochrane Handbook was used to merge the mean difference and standard deviation. We used primary outcome (FPG and TG) and secondary outcome (BMI, WHR, FIN, IR, TC, HDL, LDL, W, and WC) to measure efficacy. As an assessment of the statistical heterogeneity in each pairwise comparison, we calculated the I^2^ statistic. The justification for model selection primarily based on the characteristic of the included studies (i.e., the number of intervention types) as well as the I^2^ statistic. Subgroup or sensitivity analyses were conducted to explore the sources of heterogeneity when the heterogeneity was high. If the quantity of included studies was adequate (n ≥ 10), a funnel plot was employed to assess publication bias.

We incorporated indirect comparisons with direct comparisons using random-effects network meta-analyses within a Bayesian network framework with a Monte Carlo Markov chain model using ‘gemtc 0.8 to 7’ and its dependent packages in R software (version 4.0.5, the R Foundation, https://www.r-project.org). We simultaneously conducted 4 Monte Carlo Markov Chain models and the number of simulations was set up to 20,000, with the number of iterations set up to 70,000. The network plot was used to describe the geometry of the network of evidence. All the results of network meta-analyses for binary outcomes were present in league tables with effect sizes (mean difference [MD]) and *P*-values (with a family-wise alpha level of .05) and 95% confidence interval (CI) (according to whether the CI included the null value) to assess significance. We also assessed the ranking probabilities for all AMRT by calculating the MD for each AMRT compared with any control group and counting the proportion of iterations of the Markov chain in which each AMRT had the highest MD, the second-highest, the third-highest, and so on.

Coherent assumption behind network meta-analysis is a key assumption – i.e., that direct and indirect evidence on the same comparisons do not disagree beyond chance; thus, we assessed incoherence between direct and indirect sources of evidence using the node-splitting analysis.

## 4. Results

### 4.1. Study characteristics

**Figure 1.**
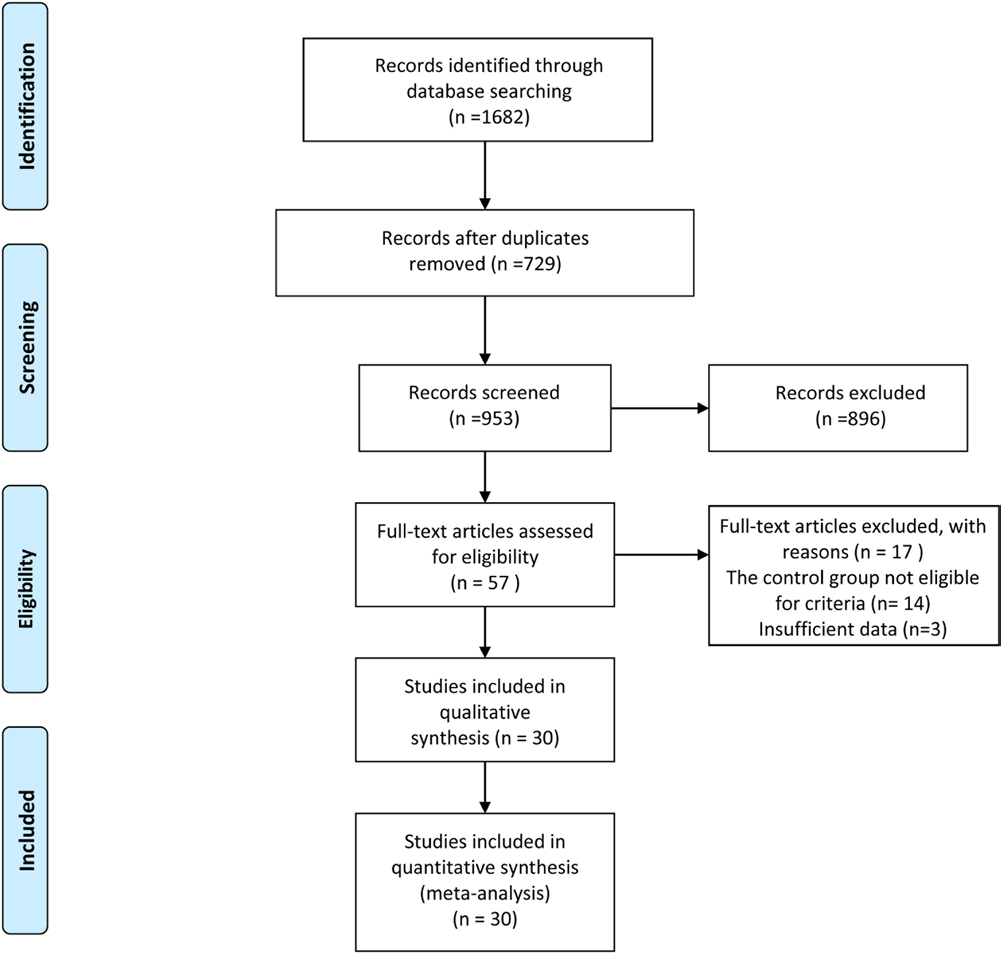
PRISMA flow diagram. PRISMA = preferred reporting items for systematic reviews and meta-analyses.

The review process, as depicted in Figure 1, and the selection of papers summarized in Table 1, resulted in 30 RCTs^[17–46]^ meeting the inclusion criteria. A total of 2589 participants, predominantly aged between 20 and 30 years, were included in the meta-analysis. Among these studies, 5 recruited obese PCOS patients specifically dealing with infertility issues. In terms of treatment modalities, 7 articles examined the use of simple acupuncture or catgut embedding in comparison to Western medicine, 7 articles provided acupuncture treatment in addition to the control group’s regimen, and only 1 article explored the addition of simple moxibustion to the control treatment. The remaining studies investigated the combined effects of acupuncture or moxibustion with other therapies. For control groups, 1 study utilized placebo acupuncture, another employed a blank control, while the majority used conventional Western medicine, predominantly metformin. The interventions spanned either 3 or 6 months/menstrual cycles. (Note: Table 1 would provide detailed information on the included studies, such as authors, year of publication, study population, intervention details, comparison groups, outcomes measured, and key findings. Similarly, Table 2 would summarize the main points of the included studies, focusing on methodologies, acupoint selections, and intervention durations.)

**Table 1:** A summary of studies meeting the inclusion criteria for the systematic review on acupuncture or moxibustion-related interventions in women with obesity-type PCOS: randomized controlled trials.

Authors, year	Age of participants			Additional type	Sample (T/C)	Intervention (T)				Intervention (C)	Outcome indicators	Intervention frequency	Intervention length
	T		C										
Li et al^[17]^	27.1 ± 2.5		25.2 ± 1.8	Infertility	25/25	Metformin + acupuncture	Acupuncture			Metformin + placebo acupuncture	BMI, WHR, FPG, FIN, IR, TG, TC, HDL, LDL	A: once a day	6 mo
Xin-xiong et al^[18]^	27.15			Infertility	30/30	Electroacupuncture + catgut embedding				Metformin	BMI, WHR	A: once a day in the first 10 d, twice daily after	3 mo
Yan et al^[19]^	28.12 ± 3.62		28.24 ± 5.41		52/52	C + Dong acupuncture				Metformin	FPG, TG, TC	A: twice a week	3 mo
Pang^[20]^	29.92 ± 3.17		30.05 ± 3.25		23/22	C + thunder-fire moxibustion				Metformin	BMI, FPG, FIN, IR	M: once every 10 d	3 menstrual cycles
J-yuan et al^[21]^	26.43 ± 3.52	26.46 ± 3.74	26.23 ± 3.48		30/30/30	C + acupuncture			Acupuncture	Metformin	BMI, WHR, FPG, IR, TG	A: once a day, 5 times a wk	3 mo
Wang and Zhongcheng ^[22]^	25.8 ± 2.6				30/30	Acupuncture + catgut embedding				Metformin	BMI, WC, W	A: once every 2 d, 6 times a wk	3 mo
Wang^[23]^	30.5 ± 2.4	30.3 ± 2.7	31.0 ± 3.1	Infertility	28/28/28	Acupuncture + Chinese medicine + Diane-35		Acupuncture + Chinese medicine		Diane-35	BMI, FPG, FIN, IR	A: once a day	3 menstrual cycles
Chun-fang et al^[24]^	31.70 ± 4.14		31.43 ± 4.60		59/59	Acupuncture + Chinese medicine				Metformin	BMI, FPG, FIN, TG, TC, HDL, LDL	A: once every 2 d	3 menstrual cycles
Wu et al^[25]^	28.64 ± 3.73		29.43 ± 2.97		56/56	C + acupuncture				Metformin	BMI	A: twice a wk	3 menstrual cycles
Chuan-hua et al^[26]^	24.07 ± 2.26		24.42 ± 3.10		40/40	Acupuncture + Chinese medicine + Moxibustion				Metformin	BMI, IR	A: twice a day M: once a day	3 mo
Min et al^[27]^	27.8 ± 4.8		28.2 ± 4.5		48/48	Electroacupuncture				Metformin	BMI, WHR, IR	A: 3 times a week	6 mo
Guochao et al^[28]^	32.75 ± 6.18		33.45 ± 6.29		53/53	C + Pulse-cutting acupuncture				Letrozole	BMI	A: 2–3 times a wk	3 mo
Jin et al^[29]^	26.03 ± 4.38	26.68 ± 4.56	27.45 ± 4.31		40/40/40	ZHU Lian Acupuncture + moxibustion	Acupuncture + moxibustion			Diane-35	BMI	A and M: once a day, 5 times a wk	3 mo
Xiaoxue et al^[30]^	28.52 ± 2.64		29.26 ± 3.27		60/60	Needle warming through moxibustion				Clomiphene citrate + urogonadotropin + chorionic gonadotropin	BMI, WHR	A and M: once every 2 d	3 menstrual cycles
Yaqin^[31]^	28.3 ± 4.2		27.9 ± 4.3		64/64	C + acupuncture				Metformin	BMI, FPG, FIN, IR	A: once a day	3 mo
Zhang^[32]^	26.3 ± 4.4		27.2 ± 4.1		50/50	C + acupuncture				Metformin	BMI, WHR	A: once a day	6 mo
Yan-Hua et al^[33]^	26.5 ± 3.0		24.9 ± 4.9		43/43	Acupuncture				Metformin	BMI, FPG, FIN, IR, TG, TC, HDL, LDL	A: once a day	6 mo
Xianbing et al^[34]^	29.5 ± 2.3				25/30/25	Electroacupuncture	Catgut embedding			Metformin	BMI, WHR, FPG, FIN	A: once every 2 d	3 mo
Zengxiu et al^[35]^	29.43 ± 4.32		28.75 ± 4.19		38/39	Acupuncture + catgut embedding				Orlistat	BMI, TG, TC	A: once a day after catgut embedding	3 mo
Xuan et al^[36]^	30.55 ± 4.67		30.25 ± 4.58		50/50	C + Acupuncture + Chinese medicine				Metformin	BMI, WHR, FIN, IR	A: once every 2 d	3 mo
Xuan^[37]^	26.59 ± 2.48		26.63 ± 2.51		36/36	C + Acupuncture + Chinese medicine				Metformin + Diane-35	BMI	A: once every 2 d	3 mo
Zhenyuan^[38]^	23.7 ± 2.2		23.1 ± 1.9	Infertility	40/40	C + Catgut embedding				Metformin	BMI, W, WC, WHR, FPG, FIN	C: once a wk	3 mo
Xiaona et al^[39]^	29.65 ± 4.32		28.20 ± 4.16		48/48	Acupuncture + Chinese medicine				Metformin	BMI, IR	A: once every 2 d	3 mo
W-hui et al^[40]^	27.23 ± 2.58		27.03 ± 3.15		30/30	Electroacupuncture + cupping				Metformin	BMI, W, WHR, FPG, FIN, IR, TG, TC, HDL, LDL	A: once every 2 d	3 mo
Xiaoli and Wenhua^[41]^	33.5 ± 3.9	32.7 ± 4.2	34.5 ± 2.5		30/30	C + Catgut embedding + moxibustion	Catgut embedding + moxibustion			Metformin + Diane-35	BMI, FPG, FIN, HDL, TG	C:Once every 10 d M:once a wk	3 mo
Na and Xiaobin^[42]^	23.81 ± 4.18	25.21 ± 4.82	24.05 ± 3.74		20/20/20	Catgut embedding + Chinese medicine	Catgut embedding			Blank control	BMI, WHR, FPG, FIN	C: once every 2 wk	3 menstrual cycles
Maohua et al^[43]^	16.7 ± 1.1		17.3 ± 1.2			Acupuncture + Chinese medicine				Metformin + Diane-35	BMI, WHR, IR, FIN	A: 3 times a wk	6 mo
Mao-hua et al^[44]^	26.5 ± 3.0		24.9 ± 4.9		43/43	Acupuncture				Metformin	BMI, FPG, FIN, IR, TG, TC, HDL, LDL	A: once a day	6 mo
Ning et al^[45]^					14/14/14	C + Abodominal acupuncture	Abdominal acupuncture			Metformin	BMI, W, WC	A: once every 2 d	6 mo
Saisai^[46]^	25.1 ± 2.3		24.1 ± 2.2	Infertility	75/75	C + acupuncture				Metformin	BMI, WHR	A: once a day	6 mo

Key points from the included studies are detailed in Table 2. The most frequently targeted meridian was the Stomach meridian, referenced 103 times. Within this meridian, the Zusanli (ST 36) acupoint was selected in 17 studies, and the Tianshu (ST 25) acupoint was chosen in 19 studies. Following this, the Ren meridian was the second most commonly utilized, cited in 94 instances, with Guanyuan (CV4) being selected 31 times and Zhongwan (CV 12) 22 times. The Spleen meridian ranked third in frequency, selected in 50 studies, where the Sanyinjiao (SP 6) acupoint was used 24 times and Daheng (SP 15) was chosen in 9 studies. The most commonly employed extra-ordinary point was Zigong (EX-CA1), which appeared in 19 studies.

A = acupuncture, BMI = body mass index, C = catgut embedding, FIN = fasting insulin, FPG = fasting blood glucose, HDL = high-density lipoprotein cholesterol, IR = insulin resistance, LDL = low-density lipoprotein cholesterol, M = moxibustion, PCOS = polycystic ovarian syndrome, TC = total cholesterol, TG = triglyceride, W = weight, WC = waistline, WHR = waist-hip rate.

**Table 2:** A summary of the main points of the included studies.

Authors, year	Main points used in AMRT
Li et al^[17]^	Zhongji (CV 3), Guanyuan (CV 4), Zigong (EX-CA1), Sanyinjiao (SP 6), Fuliu (KI 7), Zusanli (ST 36), Qixue (KI 13)
Xin-xiong et al^[18]^	Catgut embedding: Zhongwan (CV 12), Xiawan (CV 10), Tianshu (bilateral) (ST 25), Daheng (bilateral) (SP 15), Daimai (bilateral) (GB 26), Qihai (CV 6), Guanyuan (CV 4), Shuidao (bilateral) (ST 28), Guilai (bilateral) (ST 29) Acupuncture: Fuke (bilateral), Linggu (bilateral) (Dong extra-ordinary point), Sanyinjiao (bilateral) (SP 6), Xuehai (bilateral) (SP10), Zhongwan (CV 12), Xiawan (CV 10), Tianshu (bilateral) (ST 25), Daimai (bilateral) (GB 26), Qihai (bilateral) (CV 6), Guanyuan (CV 4)
Yan et al^[19]^	Zigong (EX-CA1), Guanyuan (CV 4), Dong extra-ordinary point: “Renhuang,” “Tianhuang,” “Huanchao” and “Fuke.”
Pang^[20]^	Thunder-fire moxibustion: abdomen and both ears; the acupoints of Shenque (CV 8), Fengfu (GV16) and Fengchi (GB 20) were selected. The acupoints of Pishu (BL20), Ciliao (BL32), Sanyinjiao (SP 6) and Shenshu (BL 23) were selected to exchange.
J-yuan et al^[21]^	Zhongwan (CV 12), Tianshu (ST 25), Daheng (SP 15), Damai (GB 26), Qihai (CV 6), Guanyuan (CV 4), Shuidao (ST 28), Zigong (EX-CA1), Zusanli (ST 36), Yinlingquan (SP 9), Fenglong (ST 40), Taixi (KI 3)
Wang and Zhongcheng^[22]^	Acupuncture: Liangmen (bilateral) (ST 21), Tianshu (bilateral) (ST 25), Daimai (bilateral) (GB 26), Guilai (bilateral) (ST 29), Xuehai (bilateral) (SP10), Sanyinjiao (bilateral) (SP 6); Catgut embedding: Zhongwan (bilateral) (CV 12), Tianshu (bilateral) (ST 25), Qihai (bilateral) (CV 6), Shangjuxu (bilateral) (ST 37);
Wang^[23]^	Zigong (EX-CA1), Zhongji (CV 3), Sanyinjiao (SP 6), Guanyuan (CV 4), Quchi (LI 11), Zhongwan (CV 12), Fenglong (ST 40)
Chun-fang et al^[24]^	Zhongwan (CV 12), Qihai (CV 6), Guanyuan (CV 4), Zhongji (CV 3), Zigong (EX-CA1), Dahe (KI 12) and Wailing (ST 26); the auxiliary acupoints are Zusanli (ST 36), Fenglong (CV 6) and Sanyinjiao (SP 6).
Wu et al^[25]^	Xiawan (CV 10), Zhongwan (CV 12), Guanyuan (CV 4), Qihai (CV 6), Tianshu (ST 25), Shuidao (ST 28) and bilateral Liangmen (ST 21) acupoints
Chuan-hua et al^[26]^	Qixue (KI 13), Zhongzhu (KI 15), Taixi (KI 3), Zhongwan (CV 12), Tianshu (ST 25), Daheng (SP 15), Qihai (CV 6), Guanyuan (CV 4), Zusanli (ST 36), Sanyinjiao (SP 6), Yinlingquan (SP 9), Fenglong (ST 40), Zhigou (SJ 6), Taichong (LR 3)
Min et al^[27]^	Danzhong (CV 17), Ganshu (bilateral) (BL 18), Tianshu (bilateral) (ST 25), Zigong (bilateral) (EX-CA1), Zusanli (bilateral) (ST 36), Qimen (bilateral) (LR 14), Zhongwan (CV 12), Guanyuan (CV 4), Sanyinjiao (bilateral) (SP 6), and Taichong (bilateral) (LR 3).
Guochao et al^[28]^	Zhongwan (CV 12), Qihai (CV 6), Zigong (EX-CA1), Sanyinjiao (SP 6), Xuehai (SP 10), Zusanli (ST 36), Taixi (KI 3), Zhaohai (KI 6), Shuidao (ST 28), Zhongji (CV 3), Guanyuan (CV 4), Dahe (KI 12), Zhangmen (LR 13), Qimen (LR 14), Damai (GB 26), Tianshu (ST 25), Daheng (SP 15), Fenglong (ST 40), Quchi (LI 11), Shangjuxu (ST 37)
Jin et al^[29]^	Guanyuan (CV 4), Zhongji (CV 3), Tianshu (bilateral) (ST 25), Daheng (bilateral) (SP 15), Zusanli (bilateral) (ST 36), Sanyinjiao (bilateral) (SP 6), Pishu (bilateral) (BL20), Shenshu (bilateral) (BL23), Guanyuanshu (bilateral) (BL20) and Ciliao (bilateral) (BL 32).
Xiaoxue et al^[30]^	(1) Main acupoints: Zhongwan (CV 12), Xiawan (CV 10), Guanyuan (CV 4), Tianshu (ST 25), Qihai (CV 6), bilateral Shuidao (ST 28), bilateral Zigong (EX-CA1) and bilateral Liangmen (ST 21). (2) Main points: bilateral Pishu (BL 20), Geshu (BL 17), Shenshu (BL 23), Ganshu (BL 18), Guanyuanshu (BL20) and Ciliao (BL 32)
Yaqin^[31]^	Zhongji (CV 3), Guanyuan (CV 4), Zigong (EX-CA1), Sanyinjiao (SP 6), Fuliu (KI 7), Zusanli (ST 36), Qixue (KI 13), Xuehai (SP 10), Zhongshu (DU 7).
Zhang^[32]^	Zhongji (CV 3), Guanyuan (CV 4), Zigong (EX-CA1), Sanyinjiao (SP 6), Fuliu (KI 7), Zusanli (ST 36), Qixue (KI 13)
Yan-Hua et al^[33]^	Guanyuan (CV4), Qihai (CV6), Xiawan (CV10), and Zhongwan (CV12), Liangmne (ST21), Tianshu (ST25), and Shuidao (ST28)
Xianbing et al^[34]^	Acupuncture: Zhongwan (CV 12), Liangmen (ST21), Tianshu (ST 25), Daimai (GB 26), Qihai (CV 6), Guanyuan (CV 4), Shuidao (ST28), Xuehai (SP 10), Liangqiu (ST34), Zusanli (ST 36), Shangjuxu (ST 37), Sanyinjiao (SP 6) Catgut embedding: Zhongwan (CV 12), Liangmen (ST21), Tianshu (ST25), Daimai (GB 26), Guanyuan (CV 4), Shuidao (ST28), Xuehai (SP 10), Zusanli (ST 36), Sanyinjiao (SP 6), Dachangshu (BL 25), Dazhui (DU 14)
Zengxiu et al^[35]^	Catgut embedding: Zusanli (ST 36), Fenglong (ST 40), Yinlingquan (SP 9), Futu (ST 32), Taichong (ST 32), Naohui (SJ 13), Xiaoluo (SJ 12), Binao (LI 14), Quchi (LI 11), Shangwan (CV 13), Zhongwan (CV 12), bilateral Tianshu (ST25), bilateral Daheng (SP 15), bilateral Shuidao (ST 28), bilateral Guilai (ST 29), bilateral Siman (KI 14), bilateral Daimai (GB 26) Acupuncture: Shangwan (CV 13), Zhongwan (CV 12), Guanyuan (CV 4), bilateral Tianshu (ST25), bilateral Daheng (SP 15), bilateral Shuidao (ST 28), bilateral Guilai (ST 29), bilateral Siman (KI 14), bilateral Daimai (GB 26), Luanchao, Zigong (EX-CA1), Liangmen (ST21), Shangjuxu (ST 37), Taichong (ST 32)
Xuan et al^[36]^	Follicular phase: Zigong (EX-CA1), Guanyuan (CV 4), Qihai (CV 6), Tianshu (ST25), Sanyinjiao (SP 6), Guilai (ST 29), Dahe Guilai (KI 12), Jingmen (GB25); Ovulatory stage: Zigong (EX-CA1), Guanyuan (CV 4), Qihai (CV 6), Tianshu (ST25), Sanyinjiao (SP 6), Xuehai (SP 10), Qimen (LR 14), Yinlingquan (SP 9), Sanyinjiao (SP 6), Wushu (GB 27). Luteal phase: Zigong (EX-CA1), Guanyuan (CV 4), Qihai (CV 6), Zhongwan (CV 12), Shuifen (CV 8), Zhangmen (LR 13); Back-shu points and Front-mu points: Ganshu (BL 18), Pishu (BL 20), Weishu (BL 21), Shenshu (BL 23), Dachangshu (BL 25).
Xuan^[37]^	Qihai (CV 6), Guanyuan (CV 4), Tianshu (ST25), Shenshu (BL 23), Pishu (BL 20), Diji (SP 8), Zigong (EX-CA1), Shuidao (ST 28), Guilai (ST 29), Xuehai (SP 10), Fenglong (ST 40), Liangmen (ST 21), Zusanli (ST 36), Hegu (LI4), Sanyinjiao (SP 6)
Zhenyuan^[38]^	The first group included Ganshu (BL 18), Geshu (BL23), Zhongji (CV 3), Daimai (GB 26), Guanyuan (CV 4), Zusanli (ST36) and Sanyinjiao (SP 6). The second group included Shenshu (BL 23), Pishu (BL 23), Shuihui (GV 20), Luanchao, Fenglong (ST 40), Tianshu (ST 25) and Yinlingquan (SP 9).
Xiaona et al^[39]^	Baihui (GV 20), Zhongwan (CV 12), Tianshu (ST 25), Guanyuan (CV 4), Shuidao (bilateral) (ST 28), Daimai (bilateral) (GB 26), Luanchao (bilateral), Zusanli (bilateral) (ST 36), Fenglong (bilateral) (ST 40), Sanyinjiao (bilateral) (SP 6), Taixi (bilateral) (KI 3), Taichong (bilateral) (ST 32)
W-hui et al^[40]^	Group 1: Fenglong (ST 40), Pishu (BL 20), Zusanli (ST 36), Shenshu (BL 23), Sanyinjiao (SP 6), Ganshu (BL 18) and Taixi (KI 3). Group 2: Tianshu (ST25), Zigong (EX-CA1), Guanyuan (CV 4), Xuehai (SP 10), Zusanli (ST 36), Fenglong (ST 40), Sanyinjiao (SP 6), Taixi (KI 3)
Xiaoli and Wenhua^[41]^	Moxibustion: Ren and Du meridians. Catgut embedding: Guanyuan (CV 4), Sanyinjiao (SP 6), Tianshu (ST 25), Fenglong (ST 40), Qihai (CV 6), Zhongwan (CV 12), Zigong (EX-CA1), Liangmen (ST 21)
Na and Xiaobin^[42]^	Catgut embedding: Zhongwan (CV 12), Zigong (EX-CA1), Tianshu (ST 25), Daheng (SP 15), Shuidao (ST 28), Daimai (GB 26), Guanyuan (CV 4), Shenshu (BL 23)
Maohua et al^[43]^	Zhongwan (CV 12), Tianshu (ST 25), Guanyuan (CV 4)
Mao-hua et al^[44]^	Zhongwan (CV 12), Xiawan (CV 10), Qihai (CV 6), Guanyuan (CV 4), bilateral Liangmen (ST 21), Tianshu (ST 25), Shuidao (ST 28)
Ning et al^[45]^	Tianshu (ST 25), Zhongwan (CV 12), Guanyuan (CV 4), Qihai (CV 6), Daheng (SP 15), Huaroumen (ST 24), Shuidao (ST 28), Fuai (SP 16), Fujie (SP 14), Guilai (ST 29)
Saisai^[46]^	Zhongji (CV 3), Guanyuan (CV 4), Zigong (EX-CA1), Sanyinjiao (SP 6), Fuliu (KI 7), Zusanli (ST 36), Qixue (KI 13)

AMRT = acupuncture and moxibustion-related therapy.

### 4.2. Quality assessment

The findings from the quality assessment are detailed in Supplementary Material S2, Supplemental Digital Content, https://links.lww.com/MD/P149. Regarding performance bias, due to the inherent challenges in blinding participants and personnel in interventions like acupuncture or moxibustion, only 1 study was rated as “low risk” by employing placebo acupuncture, while the rest were considered “some concerns” The mean difference in fasting plasma glucose (FPG) before treatment reported in Zhang 2017^[31]^ markedly deviated from other studies, raising concerns about data accuracy and resulting in a “high risk” rating for measurement of outcomes domain.. Notably, both the experimental design and data between Zheng 2013^[33]^ and Lai 2010^[44]^ showed high similarity, leading to a “high risk” rating for other biases in Zheng 2013.^[33]^ According to our risk-of-bias synthesis framework (Supplementary Material S3, Supplemental Digital Content, https://links.lww.com/MD/P149), the included studies’ risk percentages were as follows: high risk 10%, some concerns 86.7%, low risk 3.3%.

### 4.3. Meta-analysis results

We did direct comparisons (Table 3), showing that compared to control treatments, AMRT significantly improved BMI (MD = −1.62, 95% CI = −2.05 to −1.19), waist-to-hip ratio (WHR) (MD = −0.07, 95% CI = −0.11 to −0.04), weight (W) (MD = −2.34, 95% CI = −4.51 to −0.17), WC (MD = −3.13, 95% CI = −4.61 to −1.64), FPG (MD = −0.19, 95% CI = −0.28 to −0.09) TC (MD = −0.36, 95% CI = −0.49 to −0.23), LDL (MD = −0.20, 95% CI = −0.40 to −0.01) triglycerides (TG) (MD = −0.35, 95% CI = −0.40 to −0.29, moderate certainty) and LDL (MD = −0.20, 95% CI = −0.40 to −0.01), and showed similar benefits for Fast insulin (FIN) (MD = −0.19, 95% CI = −0.28 to −0.09), IR (MD = −0.18, 95% CI = −0.67 to 0.31), while reported less effect on HDL (MD = 0.13, 95% CI = 0.06–0.20). The detail of forest plot and funnel plot can be seen in Supplementary Material S4, Supplemental Digital Content, https://links.lww.com/MD/P149.

**Table 3:** A summary of direct meta-analysis results.

	Number of studies	Number of participants	Efficacy
AMRT vs control group			
BMI	29	2740	−1.62 (−2.05 to −1.19)
WHR	15	1381	−0.08 (−0.12 to −0.04)
W	4	242	−2.34 (−4.51 to −0.71)
WC	3	182	−3.13 (−4.61 to −1.64)
FPG	13	1166	−0.19 (−0.28 to −0.09)
FIN	14	1230	−0.94 (−2.33 to 0.45)
IR	14	1244	−0.18 (−0.67 to 0.31)
TG	14	711	−0.35 (−0.40 to −0.29)
TC	7	684	−0.36 (−0.49 to −0.23)
HDL	6	593	0.13 (0.06–0.20)
LDL	5	503	−0.20 (−0.40 to −0.01)

AMRT = acupuncture and moxibustion-related therapy, BMI = body mass index, FIN = fasting insulin, FPG = fasting blood glucose, HDL = high-density lipoprotein cholesterol, IR = insulin resistance, LDL = low-density lipoprotein cholesterol, TC = total cholesterol, TG = triglyceride, W = weight, WC = waistline, WHR = waist-hip rate.

**Figure 2.**
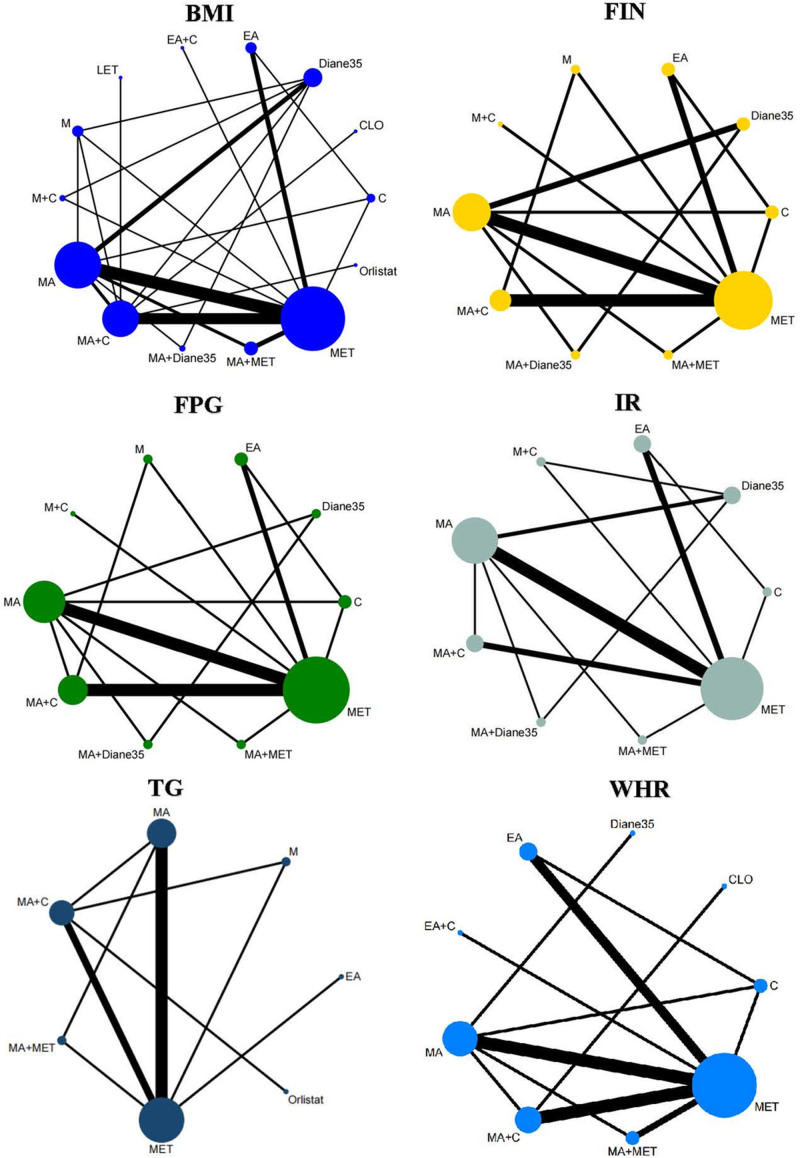
Network of eligible comparisons.

We presented all the networks for specific outcomes in Figure 2. In the network plot, the nodes and edges were weighted according to the number of interventions and comparisons. The width of the lines was proportional to the number of trials comparing every pair of treatments, and the size of each node was proportional to the number of randomly assigned participants. Too few studies included in W, WC, TC, HDL, LDL and hence it was difficult to implemented network meta analysis.

**Figure 3.**
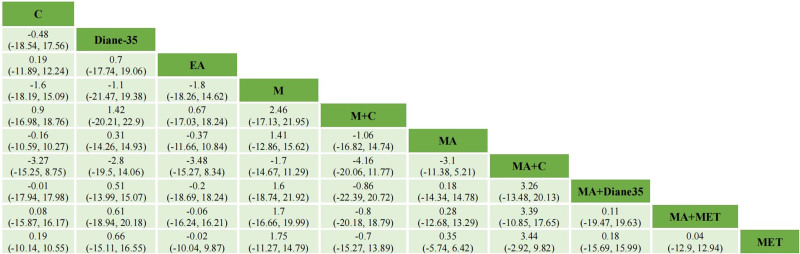
Network meta-analysis of FPG at the end of treatment. FPG = fasting plasma glucose.

**Figure 4.**
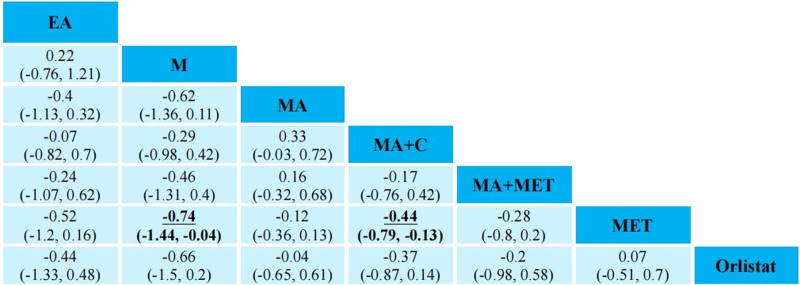
Network meta-analysis of TG at the end of treatment. C = catgut embedding, CLO = clomiphene citrate, EA = electroacupuncture, EA + catgut embedding = electroacupuncture + catgut embedding, M = moxibustion, M + catgut embedding = moxibustion + catgut embedding, MA = acupuncture, MA + C = acupuncture + catgut embedding, MA + MET = acupuncture + metformin, MA + Diane-35 = acupuncture + Diane-35, LET = letrozole, MET = metformin, TG = triglyceride.

The primary outcome results of the network meta-analysis are presented in Figures 3 and 4. In terms of FPG (13RCTs, compromising 1166 participants), there was no statistical significance among AMRT. As to TG (14RCTs, compromising 711 participants), M (MD = −0.74, 95% CI = −1.44 to −0.04) and MA + C (MD = −0.44, 95% CI = −0.79 to −0.13) were more effective than MET.

The secondary outcome results of the network meta-analysis are presented in Supplementary Materials S6 to S9, Supplemental Digital Content, https://links.lww.com/MD/P149. When it comes to BMI, MA (MD = −1.66, 95% CI = −3.15 to −0.19), MA + C (MD = −1.84, 95% CI = −3.5 to −0.22), MA + MET (MD = −2.37, 95% CI = −4.54 to −0.22), MA + Diane-35 (MD = −4.05, 95% CI = −6.9 to −1.19) were superior to Diane-35. Compared to LET, MA (MD = −5.43, 95% CI = −9.08 to −1.76), MA + C (MD = −5.61, 95% CI = −9.06 to −2.16), MA + MET (MD = −6.14, 95% CI = −10.1 to −2.2), MA + Diane-35 (MD = −7.82, 95% CI = −12.4 to −3.18), C (MD = −5.91, 95% CI = −10.06 to −1.74), EA (MD = −4.85, 95% CI = −8.85 to −0.87), EA + C (MD = −6.99, 95% CI = −12.37 to −1.57) were more effective. MA (MD = −1.44, 95% CI = −2.39 to −0.51), MA + C (MD = −1.63, 95% CI = −2.69 to −0.59), MA + MET (MD = −2.15, 95% CI = −3.78 to −0.54), MA + Diane-35 (MD = −3.84, 95% CI = −6.8 to −0.86) were superior to MET. However, both WHR and FIN haven’t shown any statistical significance among AMRT. In terms of IR, Diane-35 was less effective than M + C (MD = 1.29, 95% CI = 0.22–2.42).

**Figure 5.**
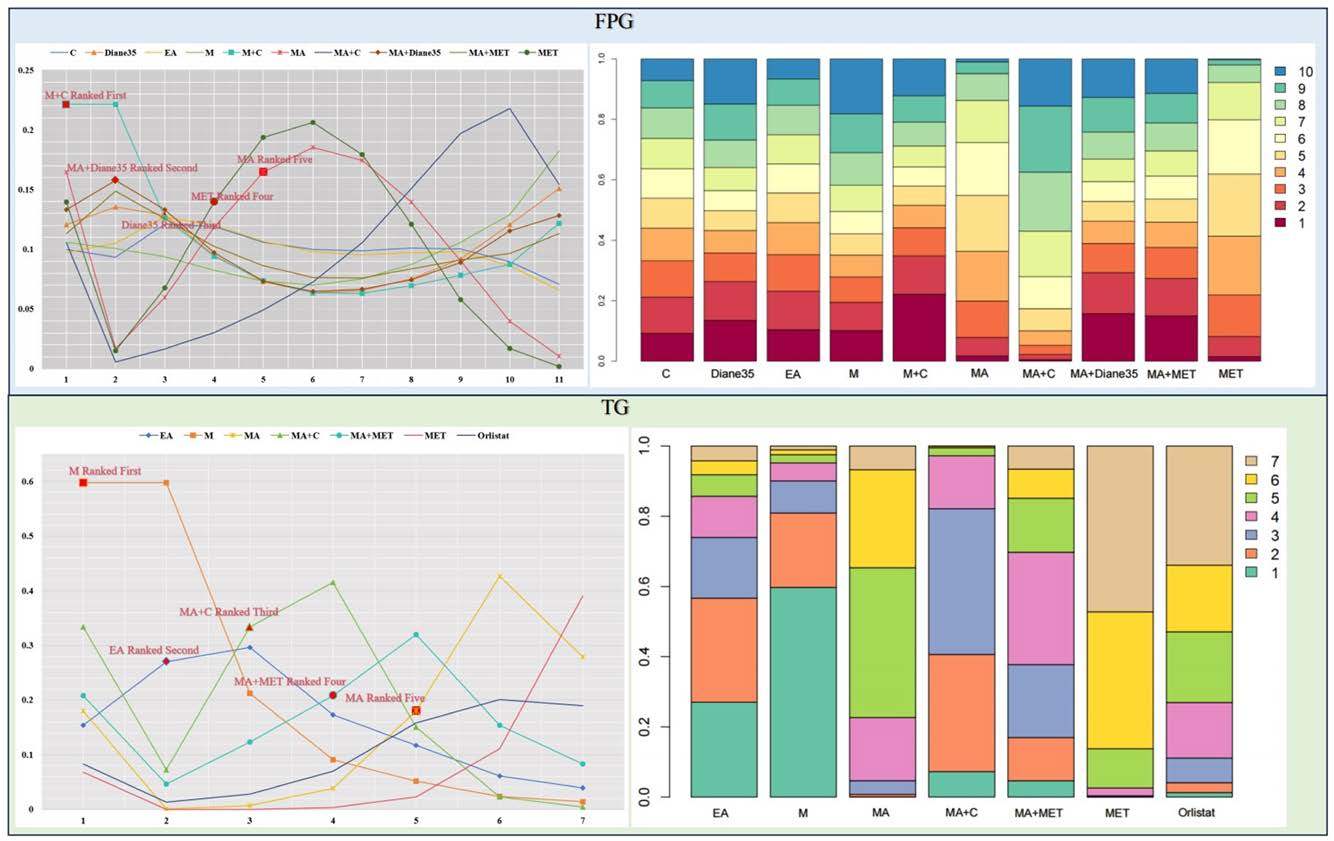
Ranking for FPG and TG. Ranking indicates the probability to be the best treatment, the second best, the third best and so on, among interventions. C = catgut embedding, CLO = clomiphene citrate, EA = electroacupuncture, EA + C = electroacupuncture + catgut embedding, LET = letrozole, M = moxibustion, M + C = moxibustion + catgut embedding, MA = acupuncture, MA + C = acupuncture + catgut embedding, MA + MET = acupuncture + metformin, MA + Diane-35 = acupuncture + Diane-35, MET = metformin, TG = triglyceride.

The FPG and TG distribution of probabilities of each AMRT being ranked at each of the possible 10/7 positions is presented in Figure 5. As to FPG, M + C, MA + Diane-35 and MET were among the most effective treatments. The largest cumulative probabilities of each AMRT being ranked at each of the possible 10 positions were (from highest to lowest rank): M + C (22.12%), MA + Diane-35 (13.31%), MET (13.97%), MA (16.46%), C (9.98%), MA + C (10.57%), EA (9.72%), M (10.60%), Diane-35 (12.07%), MA + MET (11.32%). In terms of TG, M, MA + C and MA + MET were among the most effective treatments. The largest cumulative probabilities of each AMRT being ranked at each of the possible 10 positions were (from highest to lowest rank): M (59.73%), EA (29.62%), MA + C (41.52%), MA + MET (32.00%), MA (42.65%), MET (39.03%), Orlistat (33.98%).

The rank of the other secondary outcome indicators was presented in Supplementary Materials S10 to S13, Supplemental Digital Content, https://links.lww.com/MD/P149. When it comes to BMI, MA + Diane-35, EA + C and MA + MET were among the most effective treatments. As to WHR, MA + MET, MET, MA were among the most effective treatments. In terms of FIN, M + C, MA + C and MA were among the most effective treatments. When it comes to IR, M + C, MA + C and MA + MET were among the most effective treatments.

The node-splitting analysis indicated no statistical incoherence in all comparisons of direct with indirect evidence for efficacy and treatment discontinuation.

The overall evidence certainty varied across indicators, with very low certainty for BMI, low certainty for WC, WHR, FIN, and IR moderate certainty for W, FPG, HDL, LDL, TG and TC. Simple acupuncture or catgut embedding subgroup analyses for IR yielded moderate certainty (Supplementary Material S14, Supplemental Digital Content, https://links.lww.com/MD/P149).

## 5. Discussion

This systematic review and meta-analysis evaluated the efficacy of acupuncture and moxibustion-related therapy (AMRT) on glycolipid metabolism in obese patients with polycystic ovarian syndrome (PCOS), uncovering positive outcomes with varying degrees of evidence quality. Specifically, moderate certainty was found in improvements in FPG, LDL, weight (W), and a subgroup of IR related to acupuncture or catgut embedding, whereas triglycerides (TG) and TC demonstrated high certainty in efficacy. The notable results included better effects on FPG and TG, while comparable outcomes were observed for LDL, TC, and W. However, the limited number of studies on moxibustion-related therapy calls into question the reliability of these findings. Additionally, the analyses of BMI, WHR, fasting insulin (FIN), and IR were compromised by substantial heterogeneity and low or very low evidence quality. 1 study were excluded on the discovery of heterogeneity source. The FPG number of Zhang 2017^[31]^ in the control group was obviously different from the other literatures in the same group.

Our meta-analysis distinguished itself from previous works^[11–15]^ by focusing on a broader range of interventions and specifically addressing glycolipid metabolism – a crucial but often overlooked aspect of PCOS management, particularly in obese patients. In contrast to existing literature that primarily concentrates on ovulation, menstrual regulation, and pregnancy outcomes, our work underscores the significance of metabolic health in PCOS management.

During network meta-analysis, Moxibustion combined with catgut embedding was the most effective measure in the improvement of FPG, FIN and IR. Manual acupuncture combined with metformin ranked third improving BMI, TG and IR, while ranked first in WHR.

The clinical implications of our findings are multifaceted: simple acupuncture or catgut embedding demonstrated similar efficacy to Western medicine in managing IR, with the added advantage of minimal side effects. Acupuncture-related therapies were notably effective in reducing FPG and improving lipid metabolism, affirming their potential as either standalone treatments or adjuncts to conventional medicine. From network meta-analysis, Moxibustion combined with catgut embedding was the most optimal AMRT on reducing blood glucose, while Manual acupuncture combined with metformin was the best AMRT applying on ameliorating blood lipids. Future research on AMRT, especially studies exploring moxibustion, should prioritize glycolipid metabolism outcomes and strive for methodological rigor, including standardized blinding methods.

Both M + C and MA + MET belongs to acupuncture-related therapy, which played an important role in the treatment of PCOS with obese. The summary of the main points from the included studies indicates that the stomach and spleen meridians were most frequently utilized, following the Ren meridian. Specifically, Zhongwan (CV 12) and Guanyuan (CV4), the most commonly used points in the Ren meridian, are the mu points of the stomach and the small intestine, respectively. Furthermore, Zusanli (ST 36) and Tianshu (ST 25), the most frequently used points in the Stomach meridian, are the lower He-sea point of the stomach meridian and the mu point of the large intestine, respectively. Lastly, Sanyinjiao (SP 6) represents the junction of the 3 Yin meridians, while Daheng (SP 15) is the junction between the spleen meridian and the Yin Wei meridian. These findings underscore the close relationship between acupuncture therapy and the spleen and stomach systems.

Therefore the potential mechanisms underlying the beneficial effects of acupuncture and related therapies on metabolic parameters may be attributed to their influence on the spleen and stomach meridians, pivotal in traditional Chinese medicine for the regulation of Qi and blood through the body’s transformation and transportation processes. Modern research suggests that acupuncture affects glucolipid metabolism through skin lipid metabolism, interorgan regulatory mechanisms of adipose tissue, and interactions between the nervous system and adipose tissue.^[47]^

This meta-analysis contributes to the field by: expanding the scope of intervention types analyzed and focusing on glycolipid metabolism, an area not extensively covered in previous meta-analyses. Making an indirect comparison and rank of different AMRT to find the optimal combination of Chinese and western therapy. Incorporating the GRADE evaluation system for a rigorous assessment of evidence quality. Utilizing differential values before and after treatment to mitigate the impact of baseline differences.

Nonetheless, this study is not without limitations, including a scarcity of high-quality research, absence of long-term follow-up data on glycolipid metabolism, and a lack of geographic diversity among study participants, pointing to the need for further international studies on AMRT in PCOS management.

## 6. Conclusions

Acupuncture and related therapies can effectively improve glycolipid metabolism (FPG, W, TC, TG, LDL) in obese PCOS patients, supported by moderate certainty evidence. Moreover, simple acupuncture or catgut embedding have a comparable effect to conventional medicine on improving IR, with moderate certainty evidence. Finally, moxibustion combined with catgut embedding was the most optimal AMRT on reducing blood glucose, while Manual acupuncture combined with metformin was the best AMRT applying on ameliorating blood lipids. This highlights the importance of integrating such therapies into holistic PCOS management strategies, underscoring the need for further high-quality, diverse, and long-term research in this area.

## Author contributions

**Conceptualization:** Zhili Yu, Shumin Chen, Qiao Zhang, Hongling Geng, Hao Wen, Cong Wang.

**Data curation:** Zhili Yu, Shumin Chen, Qiao Zhang, Hao Wen.

**Formal analysis:** Zhili Yu, Shumin Chen, Qiao Zhang, Hao Wen.

**Funding acquisition:** Wenbin Fu, Hongling Geng, Cong Wang.

**Investigation:** Zhili Yu, Qiao Zhang.

**Methodology:** Zhili Yu, Shumin Chen, Qiao Zhang.

**Project administration:** Zhili Yu.

**Software:** Zhili Yu, Qiao Zhang.

**Supervision:** Wenbin Fu.

**Validation:** Hongling Geng, Hao Wen.

**Visualization:** Hao Wen, Cong Wang.

**Writing – original draft:** Zhili Yu, Qiao Zhang, Hao Wen.

**Writing – review & editing:** Zhili Yu, Shumin Chen, Cong Wang.

## Supplementary Material


