# Supplementary material for: Effectiveness of acupuncture and moxibustion therapy on glycolipid metabolism in patients with obese-type polycystic ovarian syndrome: A systematic review and network meta-analysis
Source: Medicine (Baltimore). 2025 Jun 13;104(24):e42812. doi: 10.1097/MD.0000000000042812 (PMC12173337; doi:10.1097/MD.0000000000042812)
Supplement: Supplementary file 1 [file medi-104-e42812-s001.pdf]

Comparative efficacy of psychosocial interventions for opioid dependent persons receiving methadone maintenance treatment: A network meta-analysis

Supplementary Material

Content

S1. The search strategy 3
S2. Risk of bias 5
S3. PRISMA Checklist 7
S4. The Forest plot and Funnel plot 10
S5. The Trace plot, Density plot, and Gelman plot 19
S6. Network meta-analysis of BMI at the end of treatment 34
S7. Network meta-analysis of WHR at the end of treatment 35
S8. Network meta-analysis of FIN at the end of treatment 36
S9. Network meta-analysis of IR at the end of treatment 37
S10. Ranking for BMI (Ranking indicates the probability to be the best treatment, the second best, the third best and so on, among interventions). 38
S11. Ranking for WHR (Ranking indicates the probability to be the best treatment, the second best, the third best and so on, among interventions). 38
S12. Ranking for FIN (Ranking indicates the probability to be the best treatment, the second best, the third best and so on, among interventions). 39
S13. Ranking for FPG (Ranking indicates the probability to be the best treatment, the second best, the third best and so on, among interventions). 39
S14. Ranking for IR (Ranking indicates the probability to be the best treatment, the second best, the third best and so on, among interventions). 40
S15. Ranking for TG (Ranking indicates the probability to be the best treatment, the second best, the third best and so on, among interventions). 40
S16. The node-splitting analysis 41
S17. Evidence quality of acupuncture or moxibustion-related therapy 47

S1.The search strategy

Database: Medline(via Pubmed)
Retrieval time: from inception to April 26th 2024
Retrieval strategy:

(((((Randomized Controlled Trial[Publication Type])) OR (((Randomized Controll ed Trials as Topic[MeSH Terms])) OR (clinical trials[Title/Abstract]))) NOT (((mouse[Title/Abstract])) OR (rat[Title/Abstract]))) AND ((((moxibustion[MeSH Terms])) OR (moxabustion[Title/Abstract])) OR ((((((Acupuncture[MeSH Terms])) OR (Acupuncture therapy[MeSH Terms])) OR (Acupuncture Treatment[Title/Ab stract])) OR (Pharmacoacupuncture[Title/Abstract])) OR (Acupotom*[Title/Abstra ct])))) AND (((((Polycystic Ovary Syndrome[MeSH Terms])) OR (Stein Leventh al Syndrome[Title/Abstract])) OR (Sclerocystic Ovarian Degeneration[Title/Abstra ct])) OR (Sclerocystic Ovary[Title/Abstract]))

Database: Embase
Retrieval time: from inception to April 26th 2024
Retrieval strategy:

#1 'ovary polycystic disease'/exp OR 'ovary polycystic disease' OR 'stein levent hal syndrome':ab,ti OR 'sclerocystic ovarian degeneration':ab,ti OR 'sclerocystic ovary':ab,ti
#2 'acupuncture'/exp OR 'acupuncture' OR 'acupuncture treatment':ab,ti OR 'phar macoacupuncture':ab,ti OR 'acupotom*':ab,ti OR 'moxibustion'/exp OR 'moxibusti on' OR 'moxabustion':ab,ti
#3 ('randomized controlled trial':it OR 'controlled clinical trial':it OR (('clinical t rial'/exp OR 'clinical trial') AND topic) OR randomized:ab,ti OR randomised:ab, ti OR randomly:ab,ti OR placebo:ab,ti OR trial:ab,ti) NOT (('animal'/exp OR 'a nimal') NOT ('human'/exp OR 'human'))
#4 #1 AND #2 AND #3

Database: Web of science
Retrieval time: from inception to April 26th 2024
Retrieval strategy:

Web of science
#1 TS= ('Polycystic Ovary Syndrome' OR 'Stein Leventhal Syndrome' OR 'Scle rocystic Ovarian Degeneration' OR 'Sclerocystic Ovary')
#2 TS= ('Acupuncture' OR 'Acupotom*' OR 'moxibustion' OR 'moxabustion')
#3 TS=('randomized controlled trial' OR 'controlled clinical trial' OR 'clinical tri als as topic' OR randomized OR randomised OR randomly OR placebo OR tri al) NOT TS= ('animals' NOT 'humans')

#4 #1 AND #2 AND #3

Database: Cochrane Library
Retrieval time: from inception to April 26th 2024
Retrieval strategy:

#1 ("polycystic ovary syndrome"):ti,ab,kw OR (Stein Leventhal Syndrome):ti,ab,kw OR (Sclerocystic Ovarian Degeneration):ti,ab,kw OR (Sclerocystic Ovary):ti,ab,kw
#2 (Acupuncture):ti,ab,kw OR (Acupotom):ti,ab,kw OR (moxibustion):ti,ab,kw OR (moxabustion):ti,ab,kw
#3 (randomized controlled trial):ti,ab,kw OR (clinical trial):ti,ab,kw NOT (mouse):ti,ab,kw NOT (rat):ti,ab,kw
#4 #1 AND #2 AND #3

Database: CNKI
Retrieval time: from inception to April 26th 2024
Retrieval strategy:

It mainly searches for: (polycystic ovary syndrome (Mesh term) + pcos (entry term)) AND ((acupuncture (Mesh term) + Moxibustion (Mesh term)) AND (Randomized Controlled Trials as Topic (Mesh term))
(SU = '多囊卵巢综合症' OR SU = 'pcos' OR SU = '多囊') AND (SU = '针刺' OR SU = '艾灸' OR SU = '针' OR SU = '灸') AND (FT = '随机' OR FT = '临床试验' NOT FT = '动物')

Database: Wanfang
Retrieval time: from inception to April 26th 2024
Retrieval strategy:

It mainly searches for: (polycystic ovary syndrome (Mesh term) + pcos (entry term)) AND ((acupuncture (Mesh term) + Moxibustion (Mesh term)) AND (Randomized Controlled Trials as Topic (Mesh term))
(多囊卵巢综合症 OR 多囊 OR PCOS) AND (针刺 OR 针 OR 艾灸 OR 灸) AND ((随机 OR 临床试验) NOT 动物

S2. Risk of bias

S2 Figure 1 Risk of bias summary

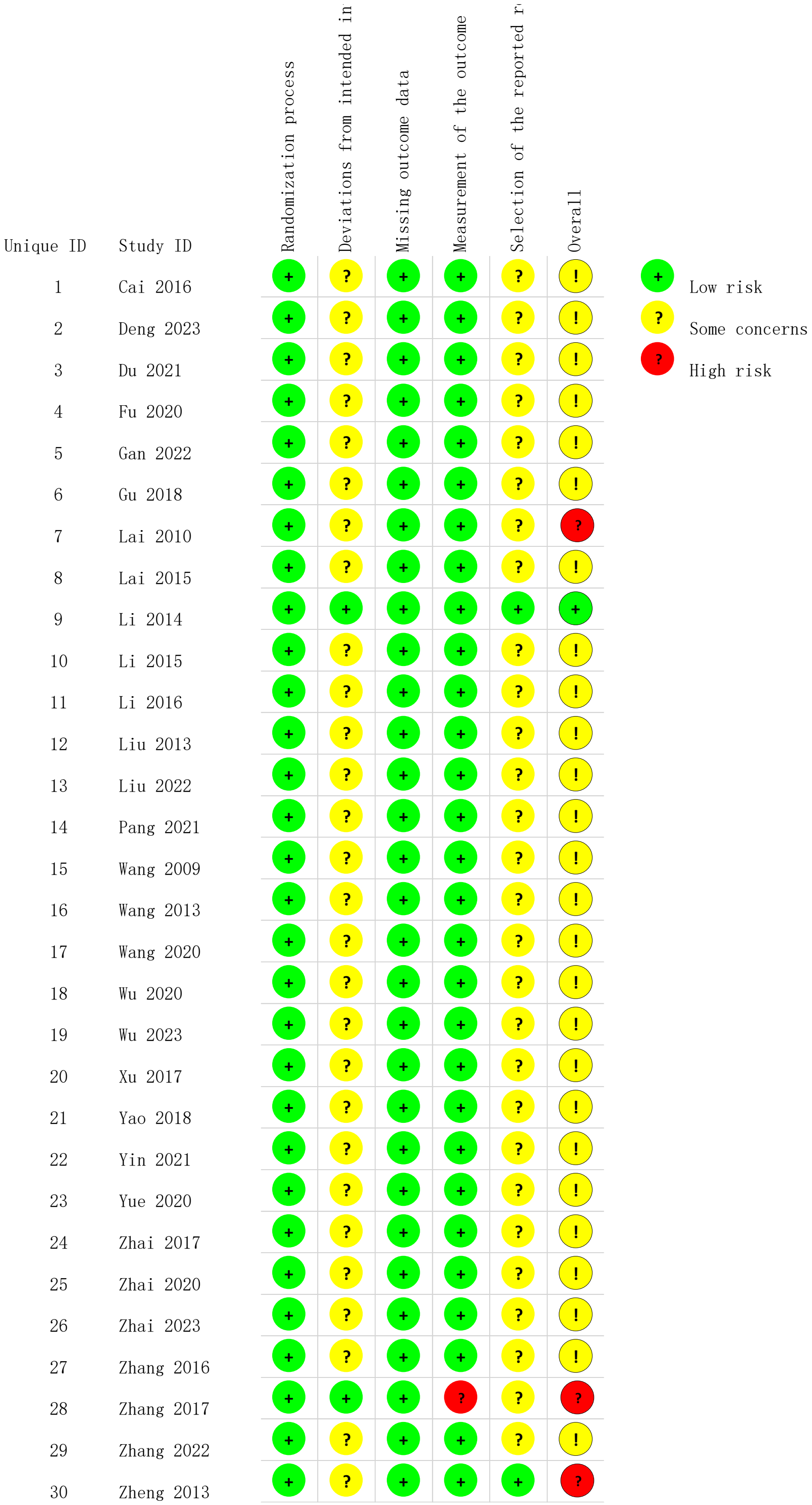

S2 Figure 2 Risk of bias graph

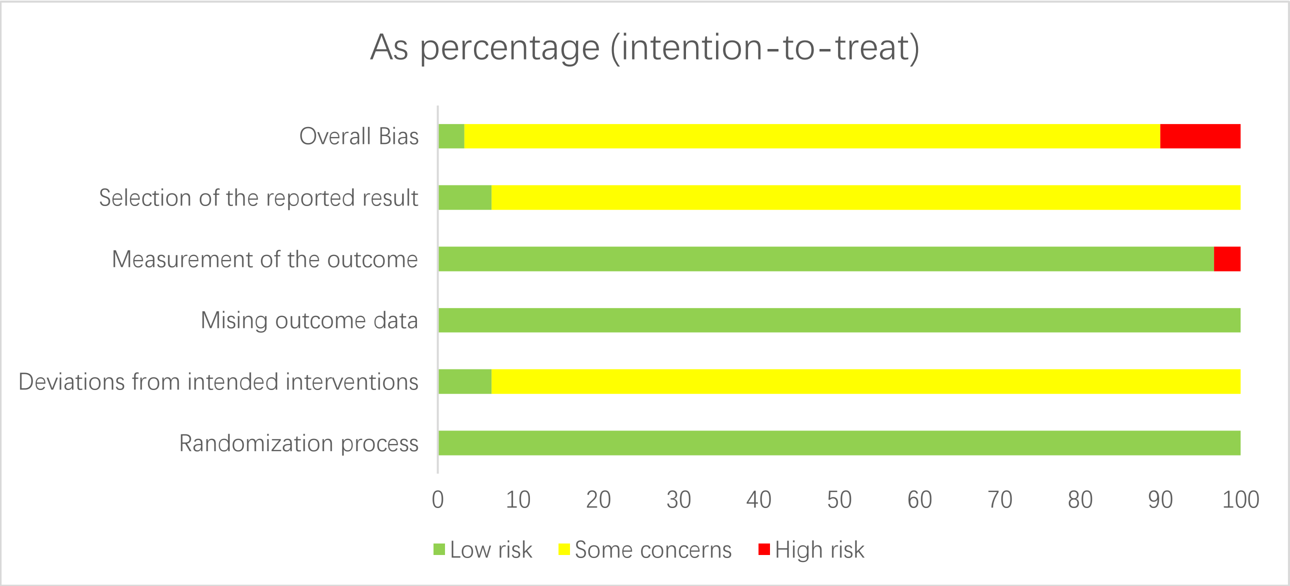
As percentage (intention-to-treat)
Overall Bias
Selection of the reported result
Measurement of the outcome
Mising outcome data
Deviations from intended interventions
Randomization process
0
10
20
30
40
50
60
70
80
90
100
Low risk
Some concerns
High risk

S3.PRISMA Checklist

S3 Table 1 PRISMA Checklist

Section/topic	#	Checklist item	Reported on page #
**TITLE**			
Title	1	Identify the report as a systematic review, meta-analysis, or both.	1
**ABSTRACT**			
Structured summary	2	Provide a structured summary including, as applicable: background; objectives; data sources; study eligibility criteria, participants, and interventions; study appraisal and synthesis methods; results; limitations; conclusions and implications of key findings; systematic review registration number.	2
**INTRODUCTION**			
Rationale	3	Describe the rationale for the review in the context of what is already known.	3
Objectives	4	Provide an explicit statement of questions being addressed with reference to participants, interventions, comparisons, outcomes, and study design (PICOS).	3
**METHODS**			
Protocol and registration	5	Indicate if a review protocol exists, if and where it can be accessed (e.g., Web address), and, if available, provide registration information including registration number.	3
Eligibility criteria	6	Specify study characteristics (e.g., PICOS, length of follow-up) and report characteristics (e.g., years considered, language, publication status) used as criteria for eligibility, giving rationale.	3-4
Information sources	7	Describe all information sources (e.g., databases with dates of coverage, contact with study authors to identify additional studies)	4

		in the search and date last searched.	
Search	8	Present full electronic search strategy for at least one database, including any limits used, such that it could be repeated.	4
Study selection	9	State the process for selecting studies (i.e., screening, eligibility, included in systematic review, and, if applicable, included in the meta-analysis).	4
Data collection process	10	Describe method of data extraction from reports (e.g., piloted forms, independently, in duplicate) and any processes for obtaining and confirming data from investigators.	4
Data items	11	List and define all variables for which data were sought (e.g., PICOS, funding sources) and any assumptions and simplifications made.	4
Risk of bias in individual studies	12	Describe methods used for assessing risk of bias of individual studies (including specification of whether this was done at the study or outcome level), and how this information is to be used in any data synthesis.	4
Summary measures	13	State the principal summary measures (e.g., risk ratio, difference in means).	4-5
Synthesis of results	14	Describe the methods of handling data and combining results of studies, if done, including measures of consistency (e.g., I) for each meta-analysis.	4-5

**Section/topic**	**#**	**Checklist item**	**Reported on page #**
Risk of bias across studies	15	Specify any assessment of risk of bias that may affect the cumulative evidence (e.g., publication bias, selective reporting within studies).	5
Additional analyses	16	Describe methods of additional analyses (e.g., sensitivity or subgroup analyses, meta-regression), if done, indicating which were pre-specified.	5
**RESULTS**			

Study selection	17	Give numbers of studies screened, assessed for eligibility, and included in the review, with reasons for exclusions at each stage, ideally with a flow diagram.	6
Study characteristics	18	For each study, present characteristics for which data were extracted (e.g., study size, PICOS, follow-up period) and provide the citations.	6
Risk of bias within studies	19	Present data on risk of bias of each study and, if available, any outcome level assessment (see item 12).	6
Results of individual studies	20	For all outcomes considered (benefits or harms), present, for each study: (a) simple summary data for each intervention group (b) effect estimates and confidence intervals, ideally with a forest plot.	6-7
Synthesis of results	21	Present results of each meta-analysis done, including confidence intervals and measures of consistency.	6-7
Risk of bias across studies	22	Present results of any assessment of risk of bias across studies (see Item 15).	8
Additional analysis	23	Give results of additional analyses, if done (e.g., sensitivity or subgroup analyses, meta-regression [see Item 16]).	8
**DISCUSSION**			
Summary of evidence	24	Summarize the main findings including the strength of evidence for each main outcome; consider their relevance to key groups (e.g., healthcare providers, users, and policy makers).	8-9
Limitations	25	Discuss limitations at study and outcome level (e.g., risk of bias), and at review-level (e.g., incomplete retrieval of identified research, reporting bias).	9-10
Conclusions	26	Provide a general interpretation of the results in the context of other evidence, and implications for future research.	10
**FUNDING**			
Funding	27	Describe sources of funding for the systematic review and other support (e.g., supply of data); role of funders for the systematic review.	NA

From: Moher D, Liberati A, Tetzlaff J, Altman DG, The PRISMA Group (2009). Preferred Reporting Items for Systematic Reviews and Meta-Analyses: The PRISMA Statement. PLoS Med 6(7): e1000097. doi:10.1371/journal.pmed1000097

S4. The Forest plot and Funnel plot

01-The forest plot, funnel plot of BMI

Study or Subgroup	Experimental Mean	Experimental SD	Experimental Total	Control Mean	Control SD	Control Total	Weight	Mean Difference IV, Random, 95% CI
**1.1.1 Acupuncture/Catgut embedding**								
Cai 2016	-4.15	2.52	55	-2.38	2.24	25	3.0%	-1.77 [-2.87, -0.67]
Gu 2018	-1.24	1.7	20	0.43	2.14	20	2.9%	-1.67 [-2.87, -0.47]
Lai 2010	-7.1	3.7	30	-4.9	4.06	30	2.1%	-2.20 [-4.17, -0.23]
Li 2014	-4.4	2.26	49	-5	2	51	3.3%	0.60 [-0.24, 1.44]
Li 2016	-1.77	1.8	14	-1.98	1.44	14	2.9%	0.21 [-1.00, 1.42]
Liu 2013	-5.1	5.8	30	-2.1	5.37	30	1.4%	-3.00 [-5.83, -0.17]
Wang 2009	-1.49	2.54	30	-2.6	4.4	30	2.3%	1.11 [-0.71, 2.93]
Wang 2020	-1.9	2.49	30	-0.66	2.55	30	2.8%	-1.24 [-2.52, 0.04]
Yao 2018	-1.7	1.93	48	-1.7	1.73	48	3.4%	0.00 [-0.73, 0.73]
Zheng 2013	-7.1	3.7	43	-4.9	4.06	43	2.4%	-2.20 [-3.84, -0.56]
**Subtotal (95% CI)**			**349**			**321**	**26.5%**	**-0.84 [-1.61, -0.07]**
Heterogeneity: Tau² = 1.02; Chi² = 32.78, df = 9 (P = 0.0001); I² = 73%								
Test for overall effect: Z = 2.15 (P = 0.03)								
**1.1.2 Acupuncture+Western medicine**								
Li 2014	-6	2.7	53	-5	2	51	3.2%	-1.00 [-1.91, -0.09]
Li 2015	-7.7	2.61	75	-5.5	2.23	75	3.3%	-2.20 [-2.98, -1.42]
Li 2016	-3.47	1.75	28	-1.98	1.44	14	3.1%	-1.49 [-2.48, -0.50]
Wang 2020	-2.31	2.42	30	-0.66	2.55	30	2.8%	-1.65 [-2.91, -0.39]
Wu 2020	-2.28	1.94	59	-2.17	2.44	59	3.3%	-0.11 [-0.91, 0.69]
Yin 2021	-8.86	5.09	53	-3.24	5.03	53	2.1%	-5.62 [-7.55, -3.69]
Zhai 2017	-1.29	0.58	40	-0.51	0.5	40	3.7%	-0.78 [-1.02, -0.54]
Zhang 2016	-8.4	2.31	50	-3.9	2.4	50	3.2%	-4.50 [-5.42, -3.58]
Zhang 2017	-5.26	2.49	64	-3.91	2.36	64	3.3%	-1.35 [-2.19, -0.51]
**Subtotal (95% CI)**			**452**			**436**	**28.1%**	**-1.94 [-2.82, -1.05]**
Heterogeneity: Tau² = 1.58; Chi² = 95.50, df = 8 (P < 0.00001); I² = 92%								
Test for overall effect: Z = 4.29 (P < 0.0001)								
**1.1.3 Acupuncture+Chinese medicine**								
Du 2021	-7.97	1.62	48	-5.27	1.85	48	3.4%	-2.70 [-3.40, -2.00]
Gu 2018	-1.31	1.9	20	0.43	2.14	20	2.8%	-1.74 [-2.99, -0.49]
Lai 2015	-5.62	3.58	30	-4.01	3.95	43	2.3%	-1.61 [-3.35, 0.13]
Wang 2013	-5	2.72	28	-0.1	2.15	28	2.8%	-4.90 [-6.18, -3.62]
Wu 2023	-2.18	0.79	56	-1.64	0.72	56	3.7%	-0.54 [-0.82, -0.26]
**Subtotal (95% CI)**			**182**			**195**	**15.1%**	**-2.27 [-3.82, -0.72]**
Heterogeneity: Tau² = 2.81; Chi² = 70.44, df = 4 (P < 0.00001); I² = 94%								
Test for overall effect: Z = 2.86 (P = 0.004)								
**1.1.4 Acupuncture+Western medicine+Chinese medicine**								
Wang 2013	-5.7	2.84	28	-0.1	2.15	28	2.8%	-5.60 [-6.92, -4.28]
Zhai 2020	-3.06	0.77	36	-1.62	0.94	36	3.6%	-1.44 [-1.84, -1.04]
Zhai 2023	-3.19	1.14	50	-4.1	1.08	50	3.6%	0.91 [0.47, 1.35]
**Subtotal (95% CI)**			**114**			**114**	**10.0%**	**-1.95 [-4.42, 0.53]**
Heterogeneity: Tau² = 4.61; Chi² = 118.59, df = 2 (P < 0.00001); I² = 98%								
Test for overall effect: Z = 1.54 (P = 0.12)								
**1.1.5 Acupuncture+moxibustion+Western/Chinese medicine**								
Gan 2022	-4.17	2.9	30	-0.98	3.85	30	2.3%	-3.19 [-4.91, -1.47]
Xu 2017	-4.48	1.87	40	-3.54	1.99	40	3.3%	-0.94 [-1.79, -0.09]
Zhang 2022	-6.66	2.8	60	-4.59	3.07	60	3.1%	-2.07 [-3.12, -1.02]
**Subtotal (95% CI)**			**130**			**130**	**8.7%**	**-1.89 [-3.08, -0.70]**
Heterogeneity: Tau² = 0.74; Chi² = 6.37, df = 2 (P = 0.04); I² = 69%								
Test for overall effect: Z = 3.12 (P = 0.002)								
**1.1.6 Acupuncture+Moxibustion/Cupping/Catgut embedding**								
Deng 2023	-4.46	1.56	38	-3.27	1.79	39	3.4%	-1.19 [-1.94, -0.44]
Fu 2020	-4.2	3.22	30	-2.02	3.31	30	2.4%	-2.18 [-3.83, -0.53]
Gan 2022	-3.38	3.27	30	-0.98	3.85	30	2.3%	-2.40 [-4.21, -0.59]
Yue 2020	-0.88	1.37	80	-0.17	1.38	40	3.5%	-0.71 [-1.23, -0.19]
**Subtotal (95% CI)**			**178**			**139**	**11.6%**	**-1.25 [-1.92, -0.57]**
Heterogeneity: Tau² = 0.21; Chi² = 5.68, df = 3 (P = 0.13); I² = 47%								
Test for overall effect: Z = 3.63 (P = 0.0003)								
**Total (95% CI)**			**1405**			**1335**	**100.0%**	**-1.62 [-2.05, -1.19]**
Heterogeneity: Tau² = 1.30; Chi² = 347.57, df = 33 (P < 0.00001); I² = 91%								
Test for overall effect: Z = 7.35 (P < 0.00001)								
Test for subgroup differences: Chi² = 5.66, df = 5 (P = 0.34), I² = 11.6%								

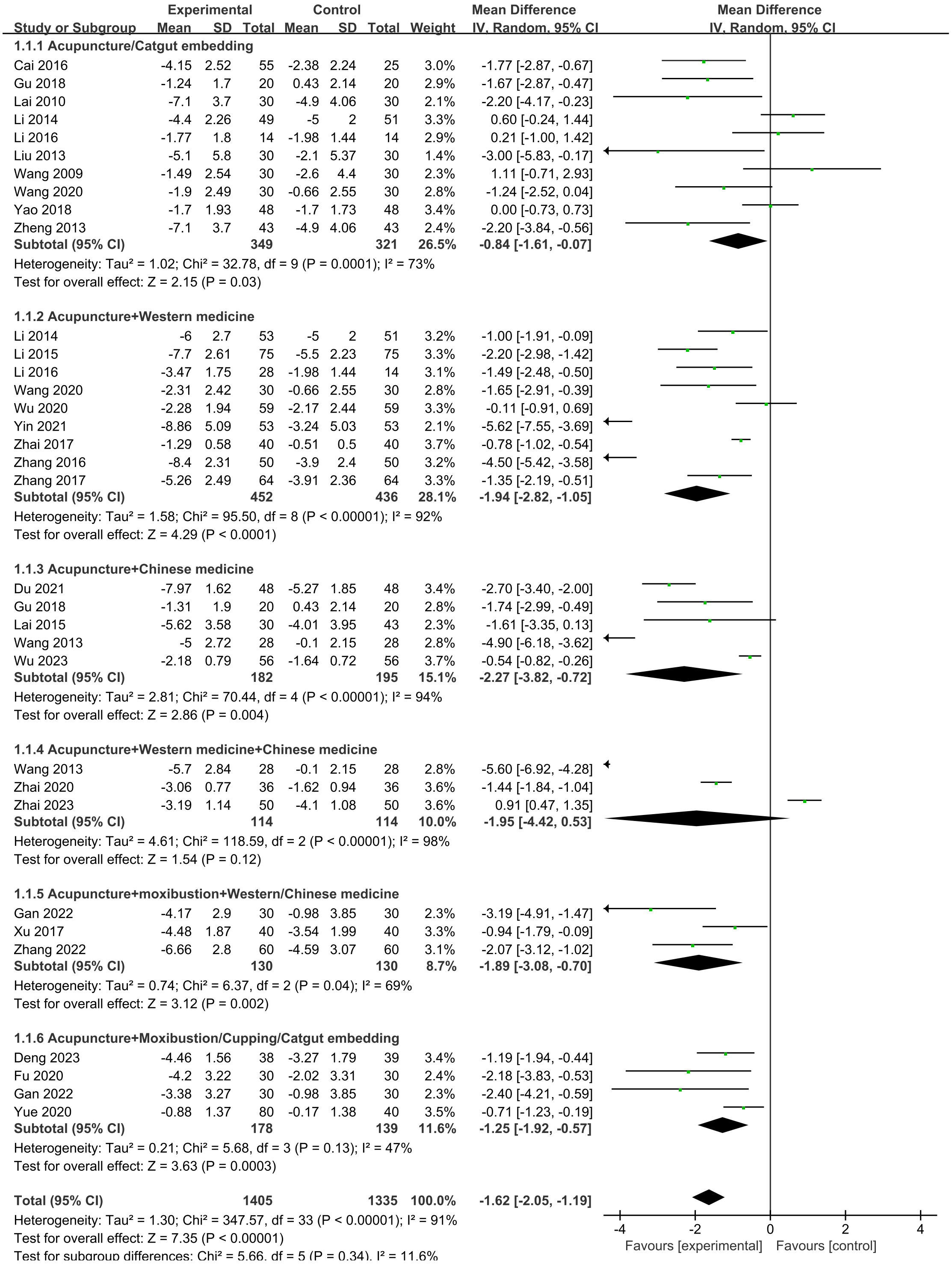

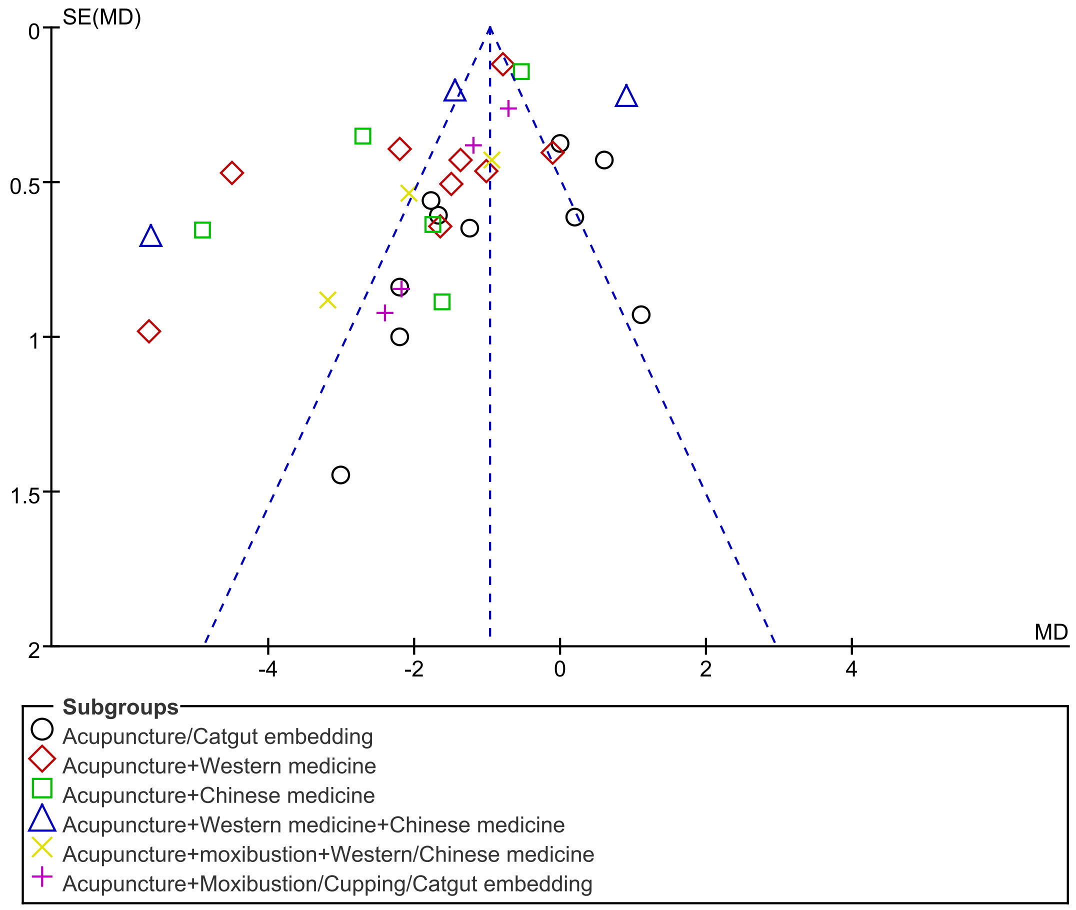

02-The forest plot, funnel plot of WHR

Study or Subgroup	Experimental Mean	Experimental SD	Experimental Total	Control Mean	Control SD	Control Total	Weight	Mean Difference IV, Random, 95% CI
**2.1.1 Acupuncture/Catgut embedding**								
Cai 2016	-0.14	0.05	55	-0.04	0.06	25	7.4%	-0.10 [-0.13, -0.07]
Gu 2018	-0.02	0.05	20	-0.02	0.04	20	7.4%	0.00 [-0.03, 0.03]
Lai 2010	-0.12	0.08	43	-0.08	0.07	43	7.3%	-0.04 [-0.07, -0.01]
Li 2014	-0.13	1.18	49	-0.1	1.9	51	0.3%	-0.03 [-0.65, 0.59]
Liu 2013	-0.04	0.07	30	-0.01	0.08	30	7.1%	-0.03 [-0.07, 0.01]
Wang 2020	-0.05	0.23	30	-0.1	0.2	30	4.3%	0.05 [-0.06, 0.16]
Yao 2018	-0.04	0.07	48	-0.03	0.08	48	7.3%	-0.01 [-0.04, 0.02]
Zheng 2013	-0.12	0.08	43	-0.08	0.07	43	7.3%	-0.04 [-0.07, -0.01]
**Subtotal (95% CI)**			**318**			**290**	**48.4%**	**-0.03 [-0.06, -0.00]**
Heterogeneity: Tau² = 0.00; Chi² = 33.36, df = 7 (P < 0.0001); I² = 79%								
Test for overall effect: Z = 2.08 (P = 0.04)								
**2.1.2 Acupuncture+Western medicine**								
Li 2014	-0.24	2.3	53	-0.1	1.9	51	0.2%	-0.14 [-0.95, 0.67]
Li 2015	-0.24	2.3	75	-0.13	0.17	75	0.4%	-0.11 [-0.63, 0.41]
Wang 2020	-0.1	0.25	30	-0.1	0.2	30	4.1%	0.00 [-0.11, 0.11]
Zhai 2017	-0.03	0.03	40	-0.01	0.04	40	7.6%	-0.02 [-0.04, -0.00]
Zhang 2016	-0.55	0.21	50	0.1	0.22	50	5.3%	-0.65 [-0.73, -0.57]
**Subtotal (95% CI)**			**248**			**246**	**17.6%**	**-0.20 [-0.54, 0.15]**
Heterogeneity: Tau² = 0.12; Chi² = 208.13, df = 4 (P < 0.00001); I² = 98%								
Test for overall effect: Z = 1.12 (P = 0.26)								
**2.1.3 Acupuncture+Chinese medicine**								
Gu 2018	-0.04	0.04	20	0.01	0.04	20	7.5%	-0.05 [-0.07, -0.03]
Lai 2015	-0.07	0.18	30	0.02	0.18	30	5.0%	-0.09 [-0.18, 0.00]
**Subtotal (95% CI)**			**50**			**50**	**12.5%**	**-0.05 [-0.08, -0.03]**
Heterogeneity: Tau² = 0.00; Chi² = 0.69, df = 1 (P = 0.41); I² = 0%								
Test for overall effect: Z = 4.32 (P < 0.0001)								
**2.1.4 Acupuncture+Moxibustion+Western medicine**								
Zhang 2022	-0.17	0.03	60	-0.09	0.05	60	7.6%	-0.08 [-0.09, -0.07]
**Subtotal (95% CI)**			**60**			**60**	**7.6%**	**-0.08 [-0.09, -0.07]**
Heterogeneity: Not applicable								
Test for overall effect: Z = 10.63 (P < 0.00001)								
**2.1.5 Acupuncture+Western medicine+Chinese medicine**								
Zhai 2023	-0.21	0.14	50	-0.15	0.14	50	6.5%	-0.06 [-0.11, -0.01]
**Subtotal (95% CI)**			**50**			**50**	**6.5%**	**-0.06 [-0.11, -0.01]**
Heterogeneity: Not applicable								
Test for overall effect: Z = 2.14 (P = 0.03)								
**2.1.6 Acupuncture+Cupping**								
Fu 2020	-0.07	0.04	30	-0.03	0.06	30	7.4%	-0.04 [-0.07, -0.01]
**Subtotal (95% CI)**			**30**			**30**	**7.4%**	**-0.04 [-0.07, -0.01]**
Heterogeneity: Not applicable								
Test for overall effect: Z = 3.04 (P = 0.002)								
**Total (95% CI)**			**756**			**726**	**100.0%**	**-0.07 [-0.11, -0.04]**
Heterogeneity: Tau² = 0.00; Chi² = 264.39, df = 17 (P < 0.00001); I² = 94%								
Test for overall effect: Z = 4.11 (P < 0.0001)								
Test for subgroup differences: Chi² = 13.39, df = 5 (P = 0.02), I² = 62.7%								

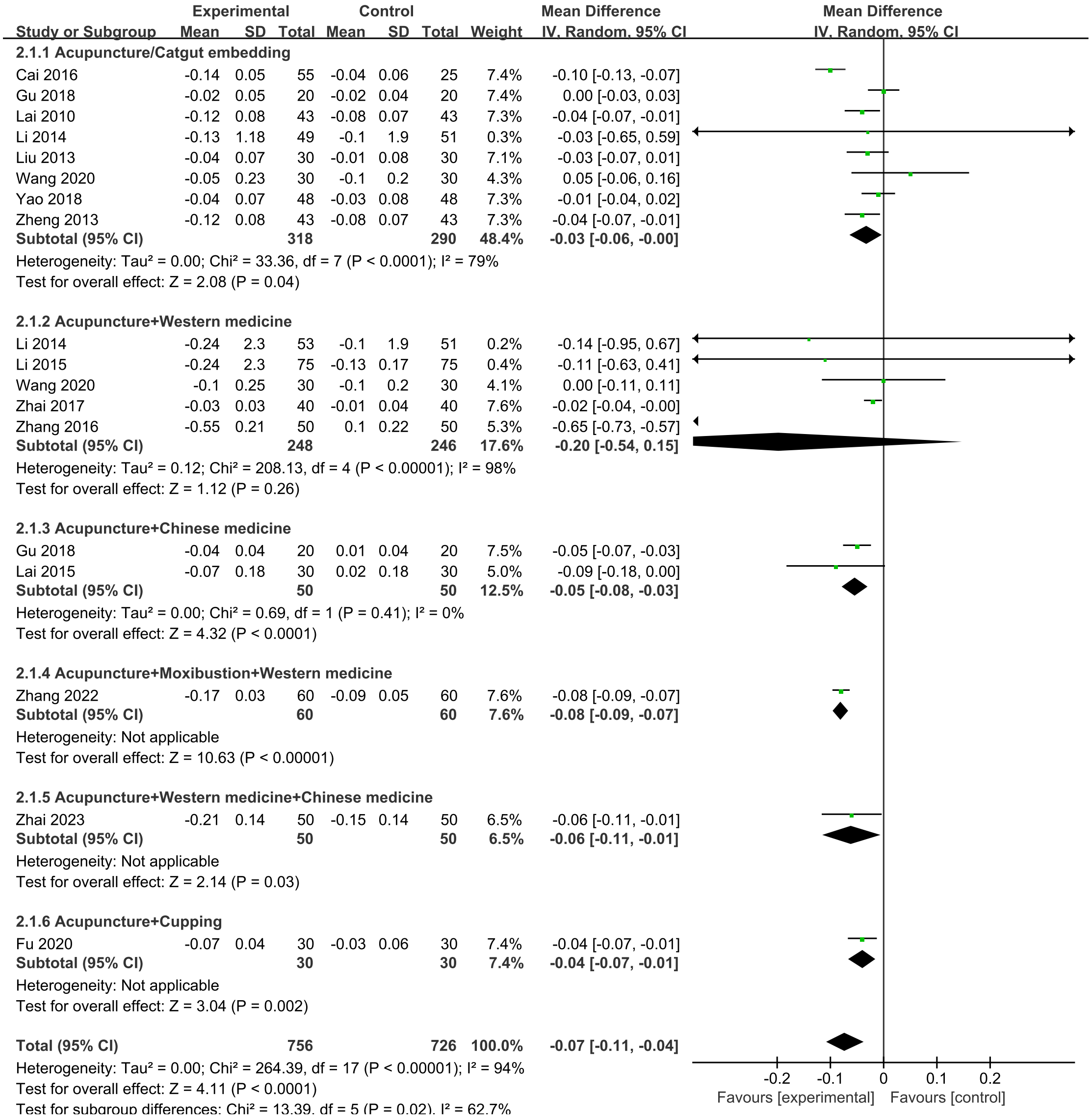

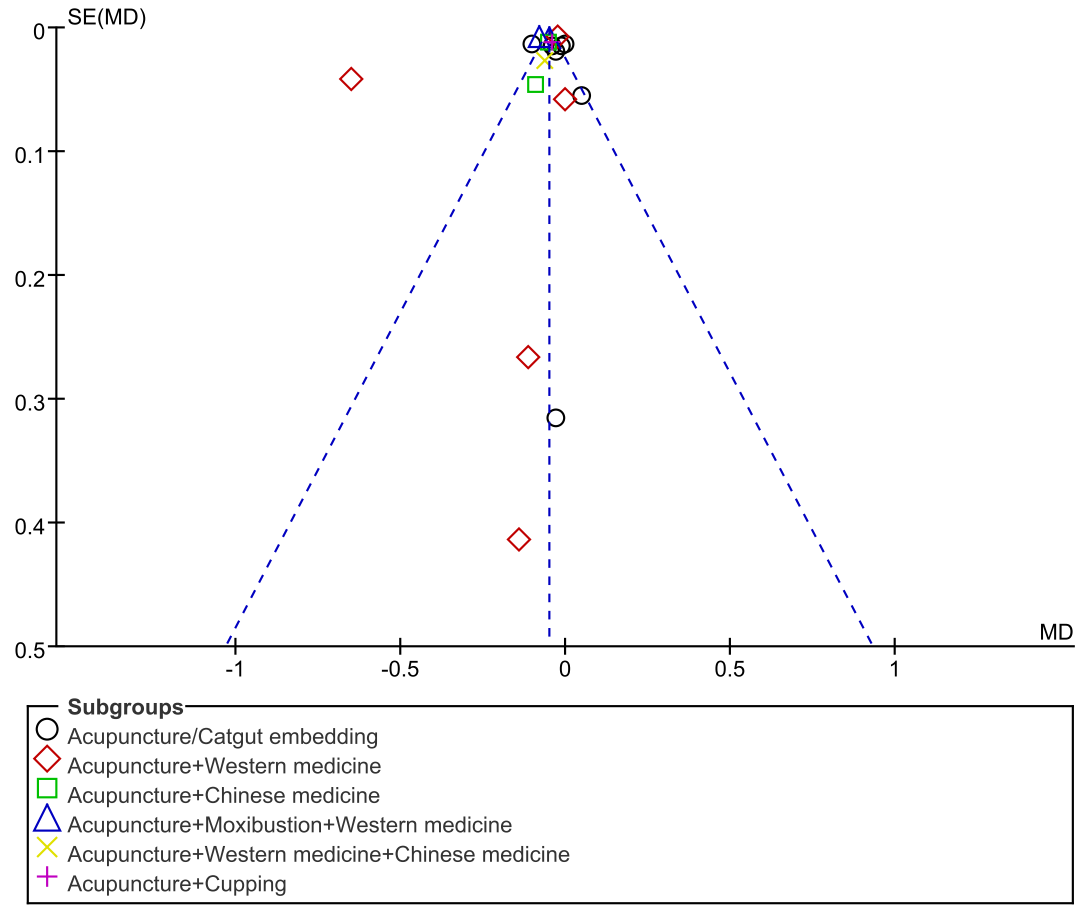

03-The forest plot of W

Study or Subgroup	Experimental Mean	Experimental SD	Experimental Total	Control Mean	Control SD	Control Total	Weight	Mean Difference IV, Fixed, 95% CI
Fu 2020	-10.81	10.23	30	-5.27	11.25	30	15.9%	-5.54 [-10.98, -0.10]
Li 2016	-6.69	5.65	28	-5.17	4.93	14	42.5%	-1.52 [-4.84, 1.80]
Wang 2009	-7.3	10.95	30	-2.3	10.78	30	15.5%	-5.00 [-10.50, 0.50]
Zhai 2017	-1.11	9.36	40	-0.97	10.01	40	26.1%	-0.14 [-4.39, 4.11]
**Total (95% CI)**			**128**			**114**	**100.0%**	**-2.34 [-4.51, -0.17]**

Heterogeneity: Chi² = 3.49, df = 3 (P = 0.32); I² = 14%
Test for overall effect: Z = 2.12 (P = 0.03)

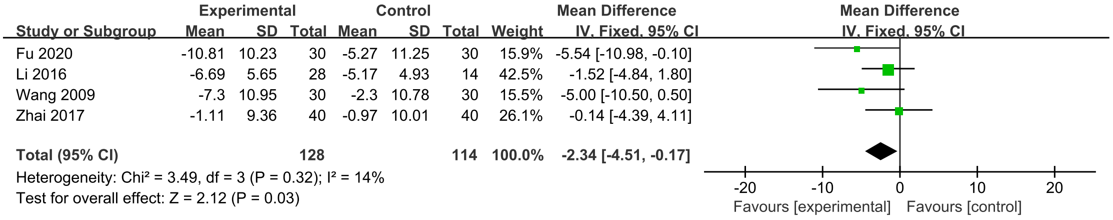

04-The forest plot of WC

Study or Subgroup	Experimental Mean	Experimental SD	Experimental Total	Control Mean	Control SD	Control Total	Weight	Mean Difference IV, Fixed, 95% CI
Li 2016	-13.8	7.81	28	-9.13	5.06	14	14.3%	-4.67 [-8.59, -0.75]
Wang 2009	-8.8	9.02	30	-1.7	9.27	30	10.3%	-7.10 [-11.73, -2.47]
Zhai 2017	-3.56	3.72	40	-1.27	4.08	40	75.4%	-2.29 [-4.00, -0.58]
**Total (95% CI)**			**98**			**84**	**100.0%**	**-3.13 [-4.61, -1.64]**

Heterogeneity: Chi² = 4.34, df = 2 (P = 0.11); I² = 54%
Test for overall effect: Z = 4.13 (P < 0.0001)

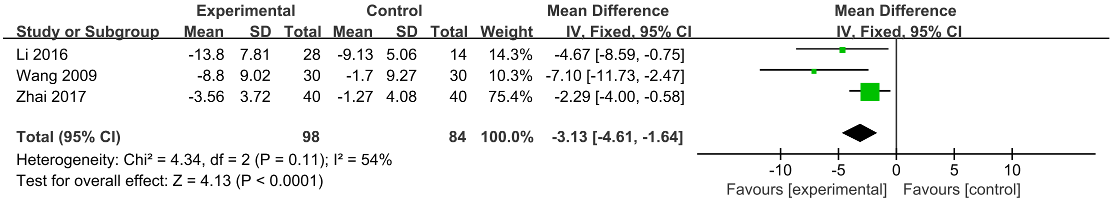

05-The forest plot, funnel plot of FPG

Study or Subgroup	Experimental Mean	Experimental SD	Experimental Total	Control Mean	Control SD	Control Total	Weight	Mean Difference IV, Fixed, 95% CI
**3.1.1 Acupuncture related therapy**								
Cai 2016	-0.57	0.64	55	-0.67	0.45	25	14.9%	0.10 [-0.14, 0.34]
Fu 2020	-0.48	0.54	60	-0.26	0.53	30	16.3%	-0.22 [-0.45, 0.01]
Gan 2022	-0.17	0.71	60	-0.13	0.72	30	9.1%	-0.04 [-0.35, 0.27]
Gu 2018	-0.08	0.38	40	0.09	0.33	20	25.7%	-0.17 [-0.36, 0.02]
Lai 2010	-0.9	1.93	43	-1	1.71	43	1.5%	0.10 [-0.67, 0.87]
Li 2014	-0.6	1.57	102	-0.1	1.82	51	2.6%	-0.50 [-1.09, 0.09]
Liu 2022	-4.53	2.47	52	-2.9	2.55	52	1.0%	-1.63 [-2.59, -0.67]
Wang 2013	-0.7	0.63	56	-0.3	0.6	28	11.7%	-0.40 [-0.68, -0.12]
Wang 2020	-1.23	1.12	60	-0.78	1.14	30	3.6%	-0.45 [-0.95, 0.05]
Wu 2023	-0.98	1.14	59	-0.88	0.97	59	6.1%	-0.10 [-0.48, 0.28]
Zhai 2017	-1.06	0.91	40	-0.75	1.08	40	4.7%	-0.31 [-0.75, 0.13]
Zhang 2017	-0.37	0.45	64	-21.24	2.89	64		Not estimable
Zheng 2013	-0.9	1.93	43	-1	1.71	43	1.5%	0.10 [-0.67, 0.87]
**Subtotal (95% CI)**			**670**			**451**	**98.6%**	**-0.18 [-0.27, -0.08]**
Heterogeneity: Chi² = 20.84, df = 11 (P = 0.04); I² = 47%								
Test for overall effect: Z = 3.70 (P = 0.0002)								
**3.1.2 Moxibustion related therapy**								
Pang 2021	-1.46	1.42	23	-0.75	1.35	22	1.4%	-0.71 [-1.52, 0.10]
**Subtotal (95% CI)**			**23**			**22**	**1.4%**	**-0.71 [-1.52, 0.10]**
Heterogeneity: Not applicable								
Test for overall effect: Z = 1.72 (P = 0.09)								
**Total (95% CI)**			**693**			**473**	**100.0%**	**-0.19 [-0.28, -0.09]**
Heterogeneity: Chi² = 22.46, df = 12 (P = 0.03); I² = 47%								
Test for overall effect: Z = 3.88 (P = 0.0001)								
Test for subgroup differences: Chi² = 1.63, df = 1 (P = 0.20), I² = 38.5%								

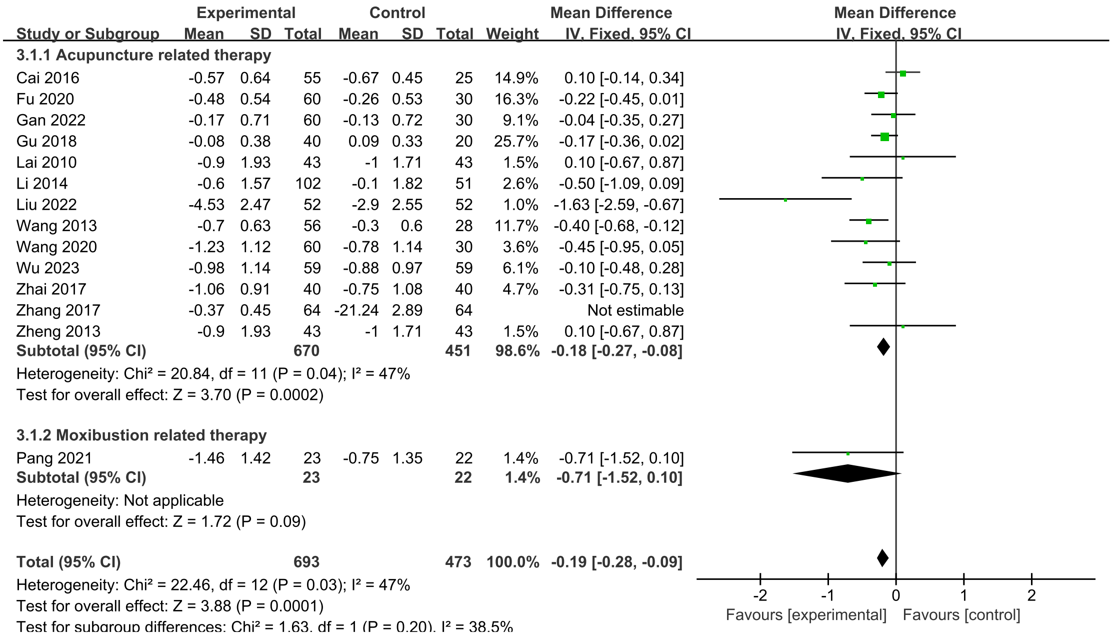

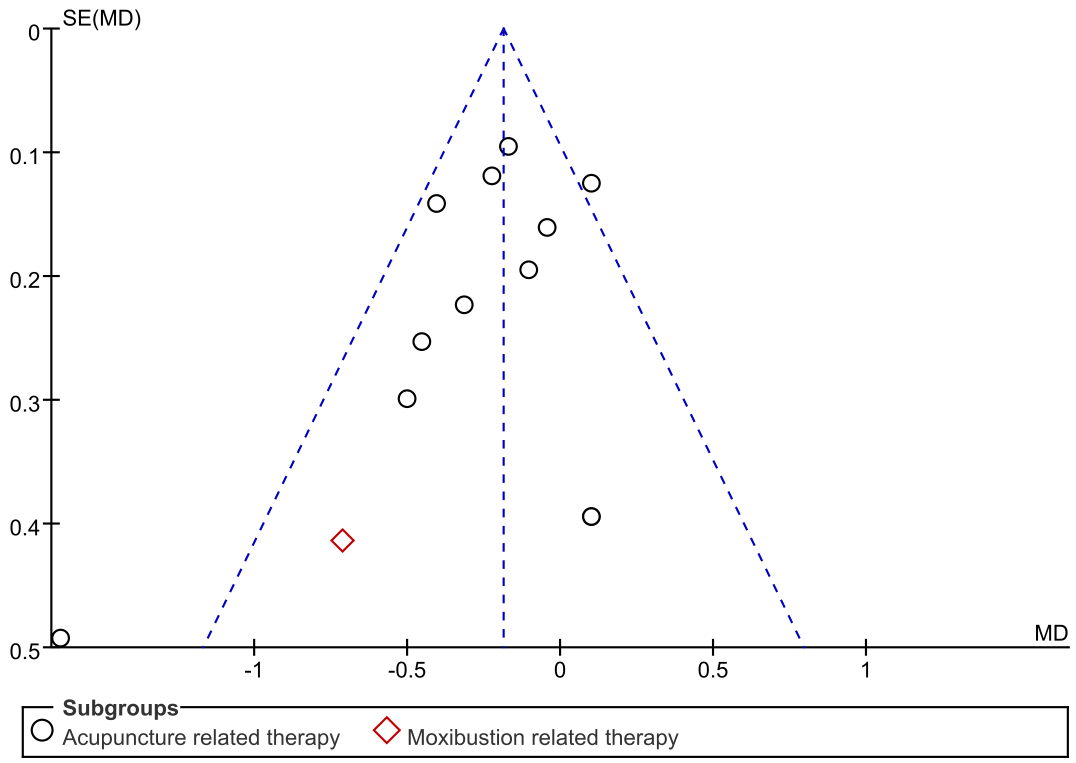

06-The forest plot, funnel plot of FIN

Study or Subgroup	Experimental Mean	Experimental SD	Experimental Total	Control Mean	Control SD	Control Total	Weight	Mean Difference IV, Random, 95% CI
**4.1.1 Acupuncture/Catgut embedding**								
Cai 2016	-4.2	3.2	55	-4.87	2.43	25	6.5%	0.67 [-0.60, 1.94]
Gu 2018	-2.22	4.37	20	-0.39	4.85	20	5.3%	-1.83 [-4.69, 1.03]
Lai 2010	-3.8	5.16	43	-4.9	6.94	43	5.5%	1.10 [-1.48, 3.68]
Li 2014	-10.4	1.93	49	-8.8	1.55	51	6.8%	-1.60 [-2.29, -0.91]
Zheng 2013	-3.8	5.16	43	-4.9	6.94	43	5.5%	1.10 [-1.48, 3.68]
**Subtotal (95% CI)**			**210**			**182**	**29.7%**	**-0.21 [-1.67, 1.24]**
Heterogeneity: Tau² = 1.77; Chi² = 14.82, df = 4 (P = 0.005); I² = 73%								
Test for overall effect: Z = 0.28 (P = 0.78)								
**4.1.2 Acupuncture+Western medicine**								
Li 2014	-10.3	2.25	53	-8.8	1.55	51	6.7%	-1.50 [-2.24, -0.76]
Zhai 2017	-8.6	6.26	40	-4.87	3.75	22	5.6%	-3.73 [-6.22, -1.24]
Zhang 2017	-17.2	11.15	64	-6.02	11.75	0		Not estimable
**Subtotal (95% CI)**			**157**			**73**	**12.4%**	**-2.28 [-4.37, -0.20]**
Heterogeneity: Tau² = 1.61; Chi² = 2.82, df = 1 (P = 0.09); I² = 65%								
Test for overall effect: Z = 2.15 (P = 0.03)								
**4.1.3 Acupuncture+Chinese medicine**								
Gu 2018	-7.14	4.2	20	-0.39	4.85	20	5.4%	-6.75 [-9.56, -3.94]
Lai 2015	-9.62	2.57	30	-5.89	4.14	30	6.2%	-3.73 [-5.47, -1.99]
Wang 2013	-5	1.99	28	-6.2	2.03	28	6.6%	1.20 [0.15, 2.25]
Wu 2023	-11.1	4.72	59	-9.12	4.53	59	6.2%	-1.98 [-3.65, -0.31]
**Subtotal (95% CI)**			**137**			**137**	**24.4%**	**-2.65 [-5.84, 0.53]**
Heterogeneity: Tau² = 9.65; Chi² = 43.57, df = 3 (P < 0.00001); I² = 93%								
Test for overall effect: Z = 1.63 (P = 0.10)								
**4.1.4 Acupuncture+Western medicine+Chinese medicine**								
Wang 2013	-1.2	1.66	28	-6.2	2.03	28	6.6%	5.00 [4.03, 5.97]
Zhai 2023	-10.67	3.03	50	-13.67	3.02	50	6.5%	3.00 [1.81, 4.19]
**Subtotal (95% CI)**			**78**			**78**	**13.2%**	**4.03 [2.07, 5.99]**
Heterogeneity: Tau² = 1.69; Chi² = 6.54, df = 1 (P = 0.01); I² = 85%								
Test for overall effect: Z = 4.03 (P < 0.0001)								
**4.1.5 Acupuncture+Moxibustion+Western medicine**								
Gan 2022	-7.36	8.44	30	-5.29	8.25	30	4.2%	-2.07 [-6.29, 2.15]
Pang 2021	-8.33	4.1	23	-4.87	3.75	22	5.8%	-3.46 [-5.75, -1.17]
**Subtotal (95% CI)**			**53**			**52**	**10.0%**	**-3.14 [-5.16, -1.13]**
Heterogeneity: Tau² = 0.00; Chi² = 0.32, df = 1 (P = 0.57); I² = 0%								
Test for overall effect: Z = 3.06 (P = 0.002)								
**4.1.6 Acupuncture+Cupping/Moxibustion**								
Fu 2020	-6.85	3.64	30	-4.31	3.71	30	6.1%	-2.54 [-4.40, -0.68]
Gan 2022	-6.23	8.13	30	-5.29	8.25	30	4.3%	-0.94 [-5.08, 3.20]
**Subtotal (95% CI)**			**60**			**60**	**10.4%**	**-2.27 [-3.97, -0.57]**
Heterogeneity: Tau² = 0.00; Chi² = 0.48, df = 1 (P = 0.49); I² = 0%								
Test for overall effect: Z = 2.62 (P = 0.009)								
**Total (95% CI)**			**695**			**582**	**100.0%**	**-0.94 [-2.33, 0.45]**
Heterogeneity: Tau² = 7.31; Chi² = 240.97, df = 16 (P < 0.00001); I² = 93%								
Test for overall effect: Z = 1.33 (P = 0.18)								
Test for subgroup differences: Chi² = 35.29, df = 5 (P < 0.00001), I² = 85.8%								

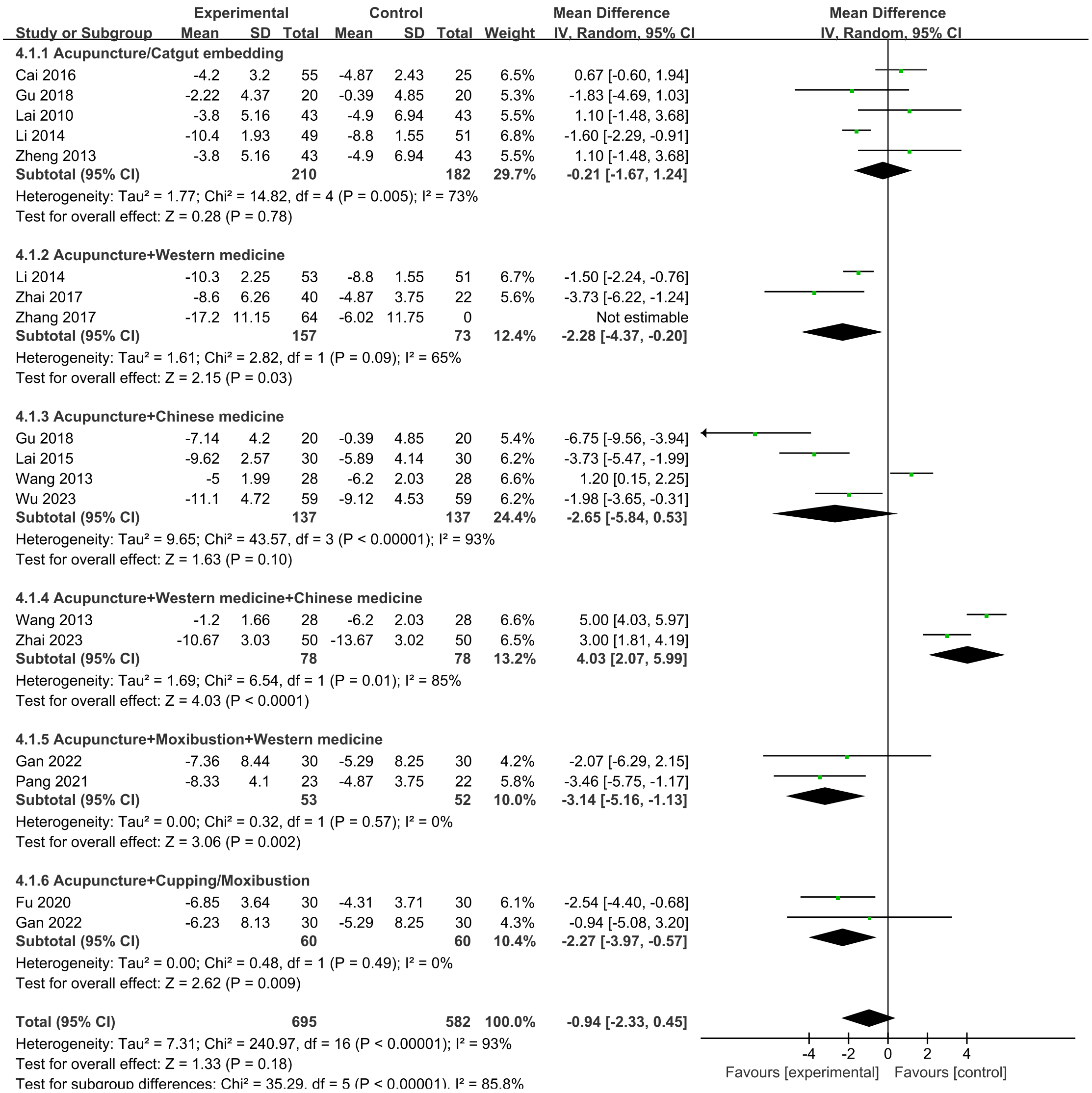

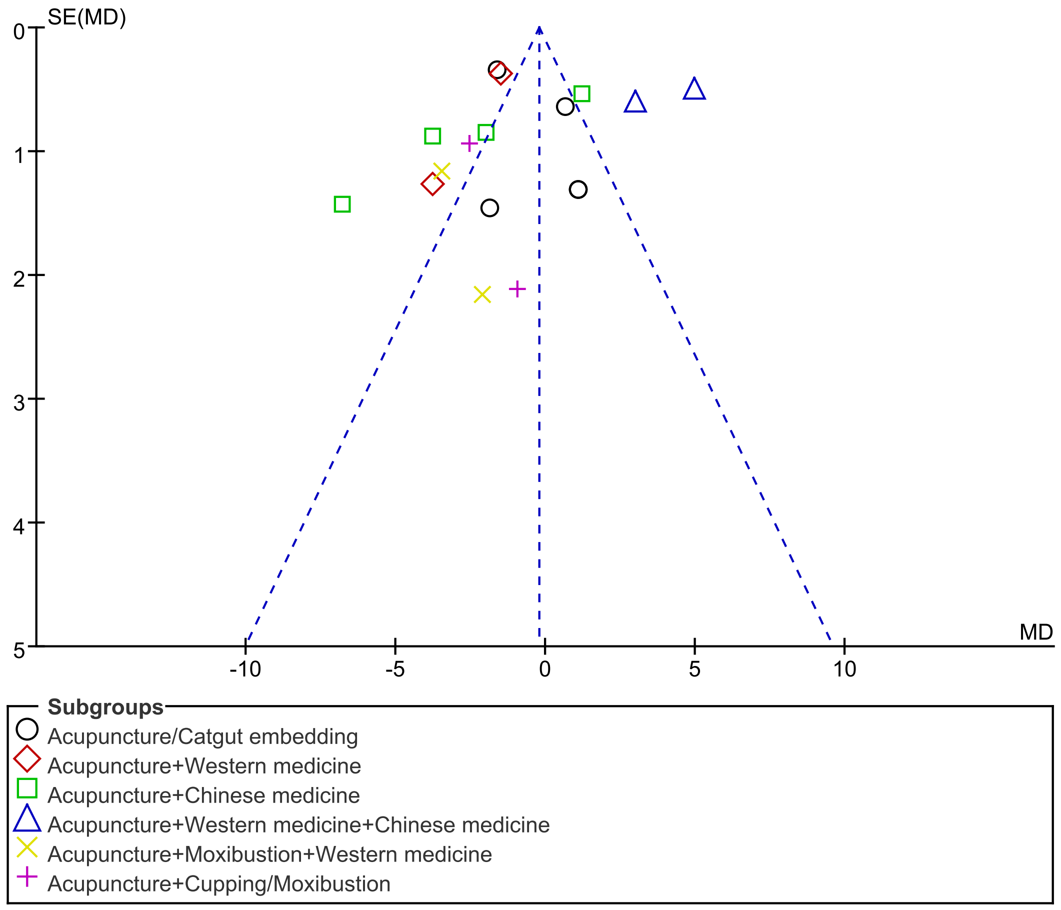

07-The forest plot, funnel plot of IR

Study or Subgroup	Experimental Mean	Experimental SD	Experimental Total	Control Mean	Control SD	Control Total	Weight	Mean Difference IV, Random, 95% CI
**5.1.1 Acupuncture**								
Cai 2016	-1.03	0.93	55	-1.21	0.82	25	6.3%	0.18 [-0.22, 0.58]
Lai 2010	-0.1	1.31	43	0.1	1.75	43	5.9%	-0.20 [-0.85, 0.45]
Li 2014	-1.6	1.06	49	-1.7	1.65	51	6.1%	0.10 [-0.44, 0.64]
Wang 2020	-3.17	0.75	30	-3.03	0.79	30	6.3%	-0.14 [-0.53, 0.25]
Yao 2018	-1.93	1.24	48	-1.83	1.23	48	6.2%	-0.10 [-0.59, 0.39]
Zheng 2013	-0.1	1.31	43	0.1	1.75	43	5.9%	-0.20 [-0.85, 0.45]
**Subtotal (95% CI)**			**268**			**240**	**36.6%**	**-0.03 [-0.23, 0.17]**
Heterogeneity: Tau² = 0.00; Chi² = 2.16, df = 5 (P = 0.83); I² = 0%								
Test for overall effect: Z = 0.33 (P = 0.75)								
**5.1.2 Acupuncture+Western medicine**								
Li 2014	-2	1.23	53	-1.7	1.65	51	6.0%	-0.30 [-0.86, 0.26]
Wang 2020	-4.06	0.77	30	-0.1	0.78	28	6.3%	-3.96 [-4.36, -3.56]
Zhang 2017	-2.3	1.2	64	-2.77	0.42	64	6.4%	0.47 [0.16, 0.78]
**Subtotal (95% CI)**			**147**			**143**	**18.7%**	**-1.26 [-4.18, 1.65]**
Heterogeneity: Tau² = 6.58; Chi² = 302.43, df = 2 (P < 0.00001); I² = 99%								
Test for overall effect: Z = 0.85 (P = 0.40)								
**5.1.3 Acupuncture+Chinese medicine**								
Du 2021	-0.79	0.51	48	-0.52	0.44	48	6.5%	-0.27 [-0.46, -0.08]
Lai 2015	-0.49	0.3	30	-0.44	0.27	30	6.5%	-0.05 [-0.19, 0.09]
Wang 2013	-1.4	0.53	28	-3.03	0.79	30	6.4%	1.63 [1.29, 1.97]
**Subtotal (95% CI)**			**106**			**108**	**19.4%**	**0.42 [-0.40, 1.24]**
Heterogeneity: Tau² = 0.51; Chi² = 93.63, df = 2 (P < 0.00001); I² = 98%								
Test for overall effect: Z = 1.00 (P = 0.32)								
**5.1.4 Acupuncture+Western medicine+Chinese medicine**								
Wang 2013	-1.3	0.61	28	-3.03	0.79	30	6.3%	1.73 [1.37, 2.09]
Zhai 2023	-3.66	0.88	50	-2.57	0.99	50	6.3%	-1.09 [-1.46, -0.72]
**Subtotal (95% CI)**			**78**			**80**	**12.7%**	**0.32 [-2.44, 3.08]**
Heterogeneity: Tau² = 3.94; Chi² = 114.95, df = 1 (P < 0.00001); I² = 99%								
Test for overall effect: Z = 0.23 (P = 0.82)								
**5.1.5 Acupuncture+Moxibustion+Chinese medicine**								
Xu 2017	-0.86	0.34	40	-0.77	0.35	40	6.5%	-0.09 [-0.24, 0.06]
**Subtotal (95% CI)**			**40**			**40**	**6.5%**	**-0.09 [-0.24, 0.06]**
Heterogeneity: Not applicable								
Test for overall effect: Z = 1.17 (P = 0.24)								
**5.1.6 Acupuncture+Cupping**								
Fu 2020	-1.91	1.06	30	-1.21	1.2	30	6.0%	-0.70 [-1.27, -0.13]
**Subtotal (95% CI)**			**30**			**30**	**6.0%**	**-0.70 [-1.27, -0.13]**
Heterogeneity: Not applicable								
Test for overall effect: Z = 2.39 (P = 0.02)								
**Total (95% CI)**			**669**			**641**	**100.0%**	**-0.18 [-0.67, 0.31]**
Heterogeneity: Tau² = 0.95; Chi² = 606.00, df = 15 (P < 0.00001); I² = 98%								
Test for overall effect: Z = 0.73 (P = 0.46)								
Test for subgroup differences: Chi² = 6.85, df = 5 (P = 0.23), I² = 27.0%								

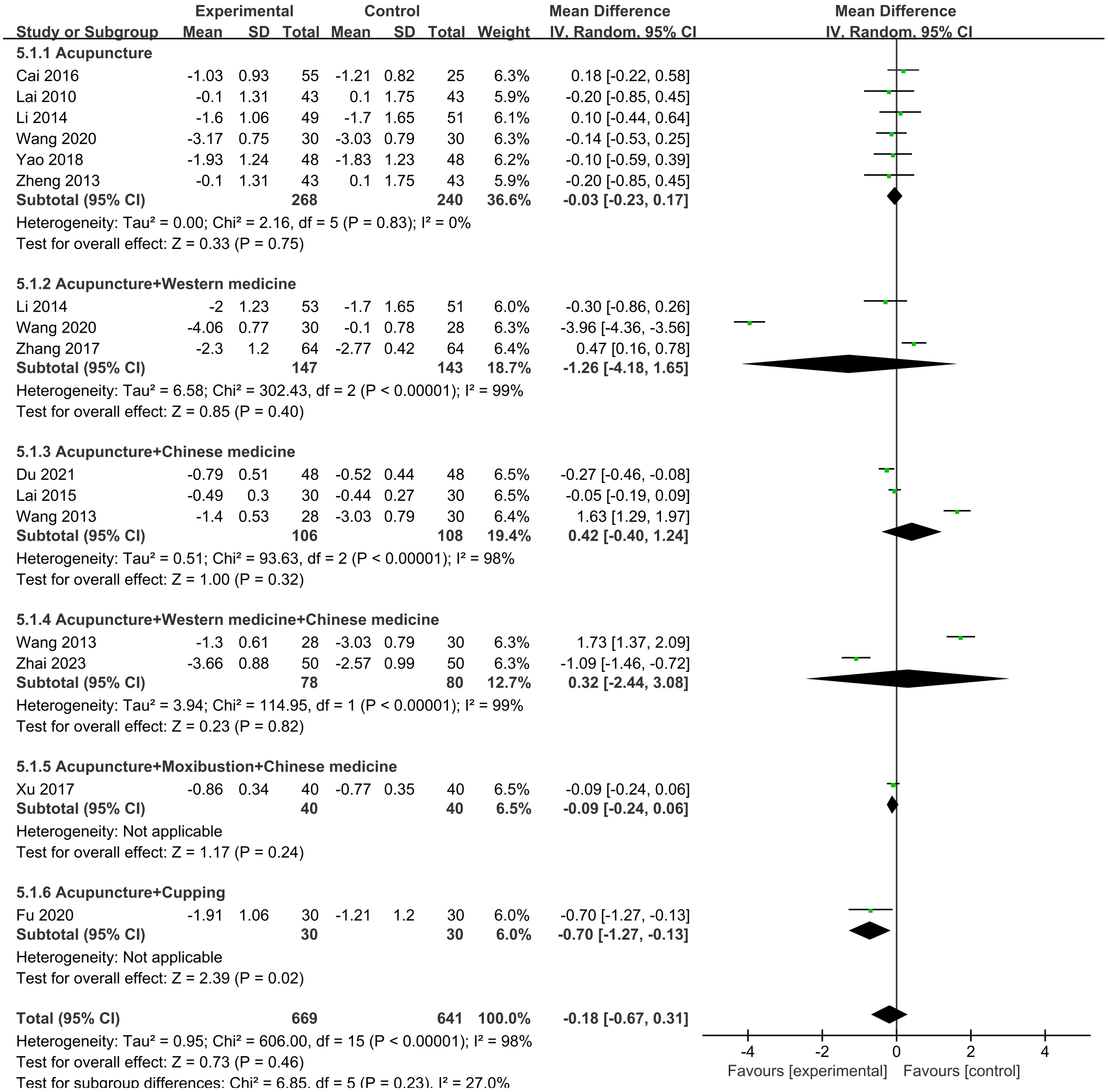

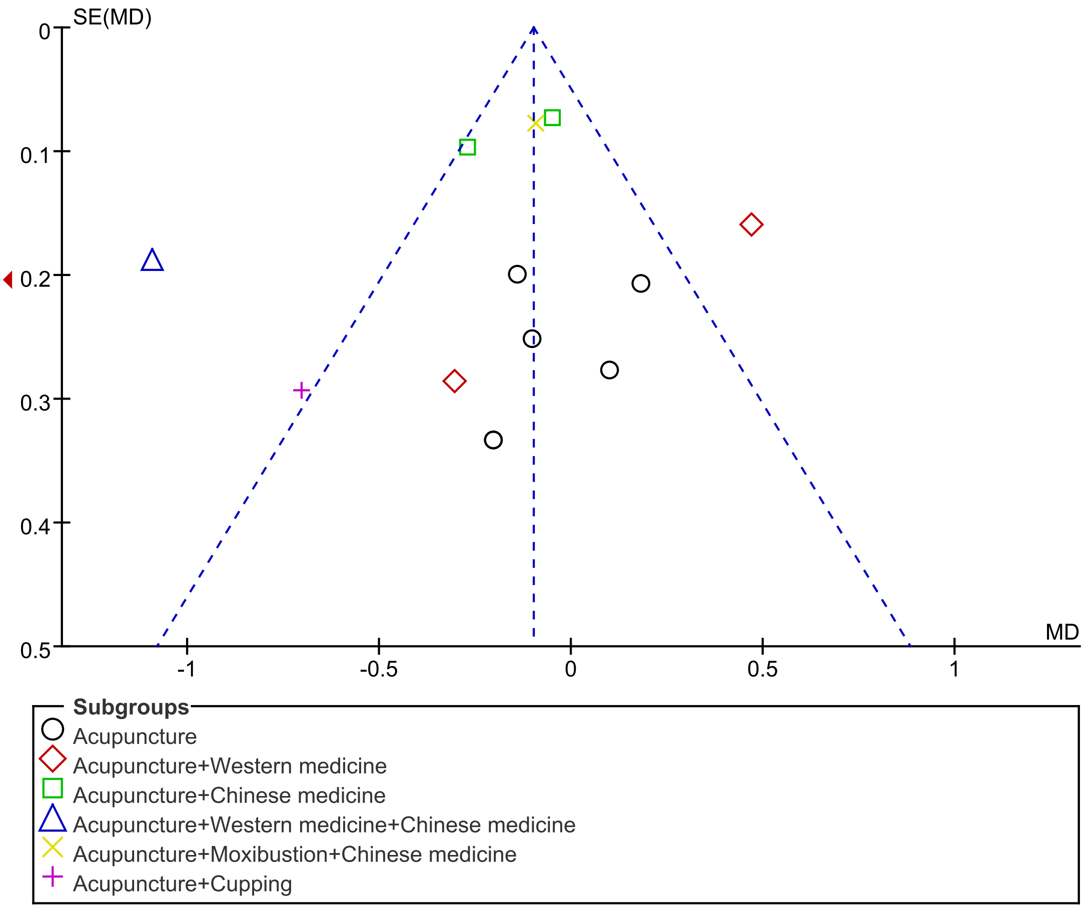

08-The forest plot of TG

Study or Subgroup	Experimental Mean	Experimental SD	Experimental Total	Control Mean	Control SD	Control Total	Weight	Mean Difference IV, Fixed, 95% CI
**6.1.1 Acupuncture related therapy**								
Deng 2023	-0.56	0.14	38	-0.19	0.14	39	71.1%	-0.37 [-0.43, -0.31]
Fu 2020	-1.04	1.06	30	-0.52	0.83	30	1.2%	-0.52 [-1.00, -0.04]
Lai 2010	-0.9	0.79	43	-0.7	0.69	43	2.8%	-0.20 [-0.51, 0.11]
Li 2014	-0.86	0.75	102	-0.8	0.17	51		Not estimable
Liu 2022	-0.87	0.64	52	-0.63	0.69	52	4.2%	-0.24 [-0.50, 0.02]
Wang 2020	-0.52	0.5	60	-0.31	0.49	30	5.9%	-0.21 [-0.43, 0.01]
Wu 2023	-0.66	0.46	59	-0.33	0.42	59	11.0%	-0.33 [-0.49, -0.17]
Zheng 2013	-0.9	0.79	43	-0.7	0.69	43	2.8%	-0.20 [-0.51, 0.11]
**Subtotal (95% CI)**			**325**			**296**	**99.1%**	**-0.34 [-0.40, -0.29]**
Heterogeneity: Chi² = 4.93, df = 6 (P = 0.55); I² = 0%								
Test for overall effect: Z = 12.68 (P < 0.00001)								
**6.1.2 Acupuncture combined with moxibustion therapy**								
Gan 2022	-1.02	1.25	60	-0.14	1.29	30	0.9%	-0.88 [-1.44, -0.32]
**Subtotal (95% CI)**			**60**			**30**	**0.9%**	**-0.88 [-1.44, -0.32]**
Heterogeneity: Not applicable								
Test for overall effect: Z = 3.08 (P = 0.002)								
**Total (95% CI)**			**385**			**326**	**100.0%**	**-0.35 [-0.40, -0.29]**
Heterogeneity: Chi² = 8.45, df = 7 (P = 0.29); I² = 17%								
Test for overall effect: Z = 12.91 (P < 0.00001)								
Test for subgroup differences: Chi² = 3.51, df = 1 (P = 0.06), I² = 71.5%								

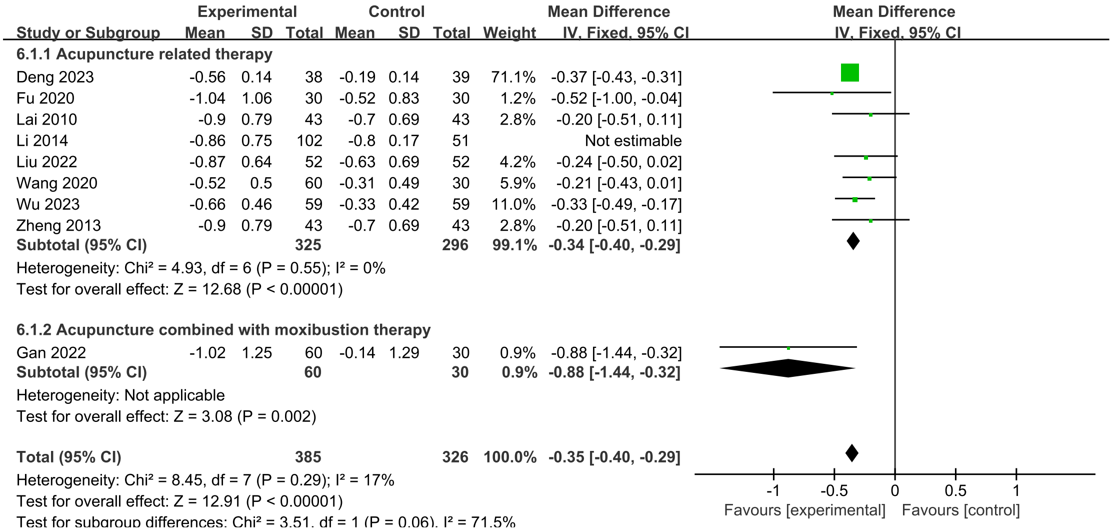

09-The forest plot of TC

Study or Subgroup	Experimental Mean	Experimental SD	Experimental Total	Control Mean	Control SD	Control Total	Weight	Mean Difference IV, Fixed, 95% CI
Deng 2023	-0.72	0.5	38	-0.18	0.51	39	32.9%	-0.54 [-0.77, -0.31]
Fu 2020	-0.69	1.03	30	-0.46	1.01	30	6.3%	-0.23 [-0.75, 0.29]
Lai 2010	-1.2	1.1	43	-1.1	1.05	43	8.1%	-0.10 [-0.55, 0.35]
Li 2014	-0.95	1.24	102	-0.8	0.98	51	12.9%	-0.15 [-0.51, 0.21]
Liu 2022	-2.4	1.48	52	-1.68	1.49	52	5.1%	-0.72 [-1.29, -0.15]
Wu 2023	-1.23	0.65	59	-0.87	0.74	59	26.5%	-0.36 [-0.61, -0.11]
Zheng 2013	-1.2	1.1	43	-1.1	1.05	43	8.1%	-0.10 [-0.55, 0.35]
**Total (95% CI)**			**367**			**317**	**100.0%**	**-0.36 [-0.49, -0.23]**

Heterogeneity: Chi² = 8.03, df = 6 (P = 0.24); I² = 25%
Test for overall effect: Z = 5.46 (P < 0.00001)

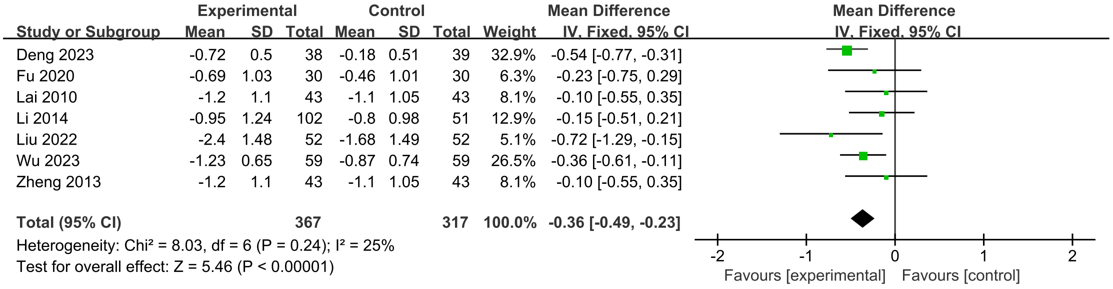

10-The forest plot of HDL

Study or Subgroup	Experimental Mean	Experimental SD	Experimental Total	Control Mean	Control SD	Control Total	Weight	Mean Difference IV, Fixed, 95% CI
**8.1.1 Acupuncture related therapy**								
Fu 2020	0.21	0.3	30	0.07	0.3	30	21.1%	0.14 [-0.01, 0.29]
Lai 2010	0.4	0.35	43	0.4	0.44	43	17.2%	0.00 [-0.17, 0.17]
Li 2014	0.5	0.52	102	0.3	0.72	51	9.9%	0.20 [-0.02, 0.42]
Wu 2023	0.37	0.38	59	0.13	0.36	59	27.2%	0.24 [0.11, 0.37]
Zheng 2013	0.4	0.35	43	0.4	0.44	43	17.2%	0.00 [-0.17, 0.17]
**Subtotal (95% CI)**			**277**			**226**	**92.6%**	**0.12 [0.05, 0.20]**
Heterogeneity: Chi² = 7.57, df = 4 (P = 0.11); I² = 47%								
Test for overall effect: Z = 3.35 (P = 0.0008)								
**8.1.2 Acupuncure combined with moxibustion therapy**								
Gan 2022	0.27	0.66	60	0.12	0.54	30	7.4%	0.15 [-0.11, 0.41]
**Subtotal (95% CI)**			**60**			**30**	**7.4%**	**0.15 [-0.11, 0.41]**
Heterogeneity: Not applicable								
Test for overall effect: Z = 1.15 (P = 0.25)								
**Total (95% CI)**			**337**			**256**	**100.0%**	**0.13 [0.06, 0.20]**

Heterogeneity: Chi² = 7.61, df = 5 (P = 0.18); I² = 34%
Test for overall effect: Z = 3.54 (P = 0.0004)
Test for subgroup differences: Chi² = 0.04, df = 1 (P = 0.85), I² = 0%

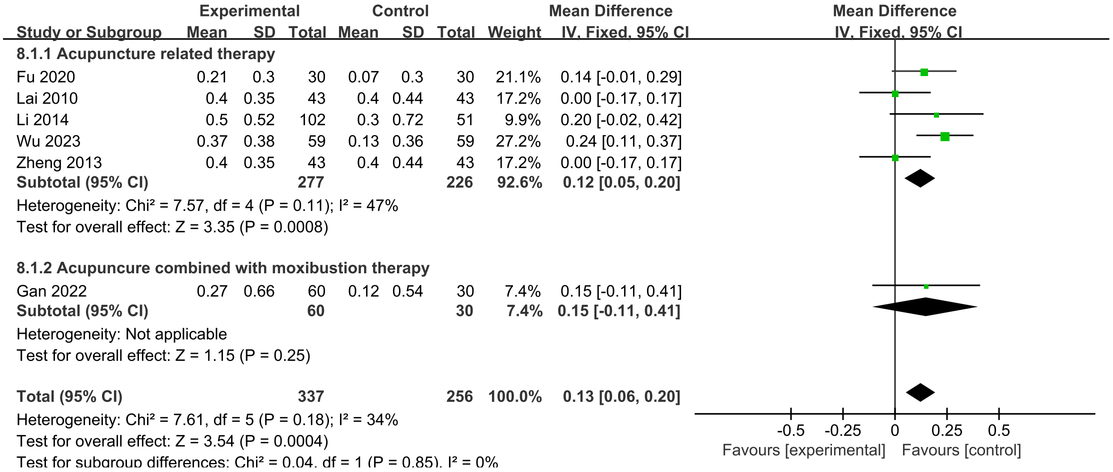

11-The forest plot of LDL

Study or Subgroup	Experimental Mean	Experimental SD	Experimental Total	Control Mean	Control SD	Control Total	Weight	Mean Difference IV, Fixed, 95% CI
Fu 2020	-0.62	0.82	30	-0.5	0.82	30	21.3%	-0.12 [-0.53, 0.29]
Lai 2010	-0.9	1.51	43	-0.6	0.92	43	13.2%	-0.30 [-0.83, 0.23]
Li 2014	-0.76	1.01	102	-1	1.71	51	14.2%	0.24 [-0.27, 0.75]
Wu 2023	-2.02	0.84	59	-1.67	0.88	59	38.1%	-0.35 [-0.66, -0.04]
Zheng 2013	-0.9	1.51	43	-0.6	0.92	43	13.2%	-0.30 [-0.83, 0.23]
**Total (95% CI)**			**277**			**226**	**100.0%**	**-0.20 [-0.40, -0.01]**

Heterogeneity: Chi² = 4.19, df = 4 (P = 0.38); I² = 4%
Test for overall effect: Z = 2.09 (P = 0.04)

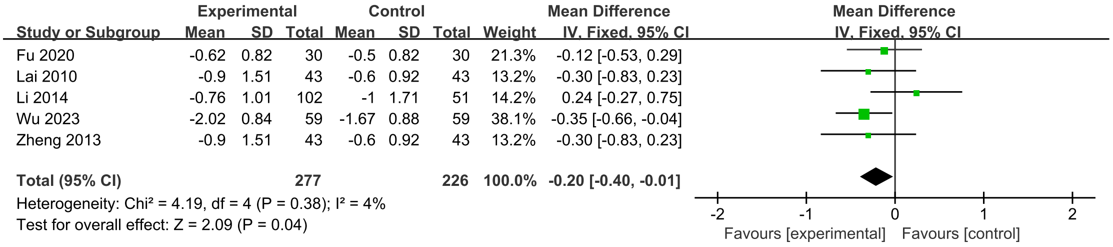

S5.The Trace plot, Density plot, and Gelman plot

01-The Trace plot, Density plot, and Gelman plot of BMI

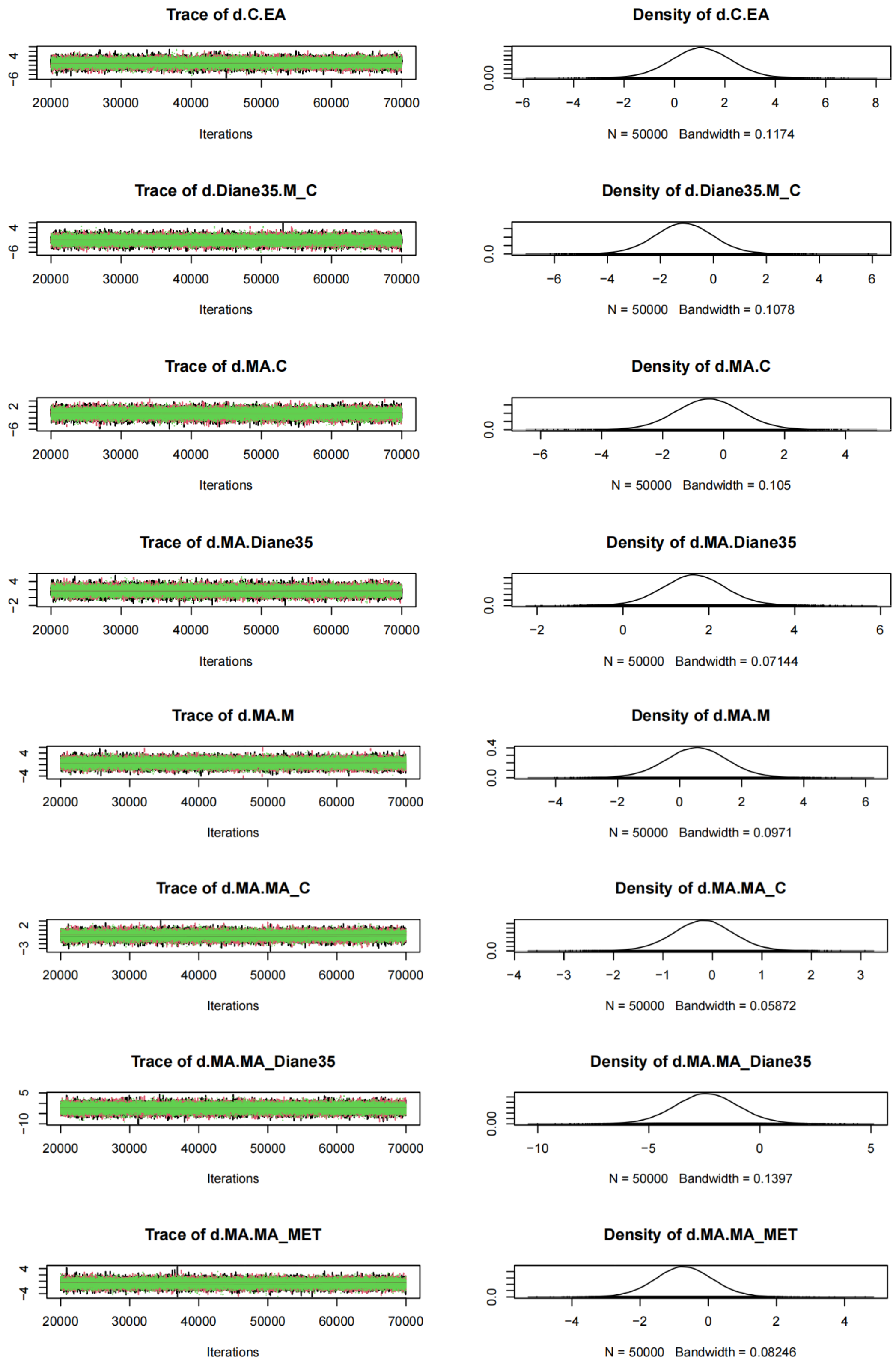
Trace of d.C.EA
Iterations
Density of d.C.EA
N = 50000 Bandwidth = 0.1174
Trace of d.Diane35.M_C
Iterations
Density of d.Diane35.M_C
N = 50000 Bandwidth = 0.1078
Trace of d.MA.C
Iterations
Density of d.MA.C
N = 50000 Bandwidth = 0.105
Trace of d.MA.Diane35
Iterations
Density of d.MA.Diane35
N = 50000 Bandwidth = 0.07144
Trace of d.MA.M
Iterations
Density of d.MA.M
N = 50000 Bandwidth = 0.0971
Trace of d.MA.MA_C
Iterations
Density of d.MA.MA_C
N = 50000 Bandwidth = 0.05872
Trace of d.MA.MA_Diane35
Iterations
Density of d.MA.MA_Diane35
N = 50000 Bandwidth = 0.1397
Trace of d.MA.MA_MET
Iterations
Density of d.MA.MA_MET
N = 50000 Bandwidth = 0.08246

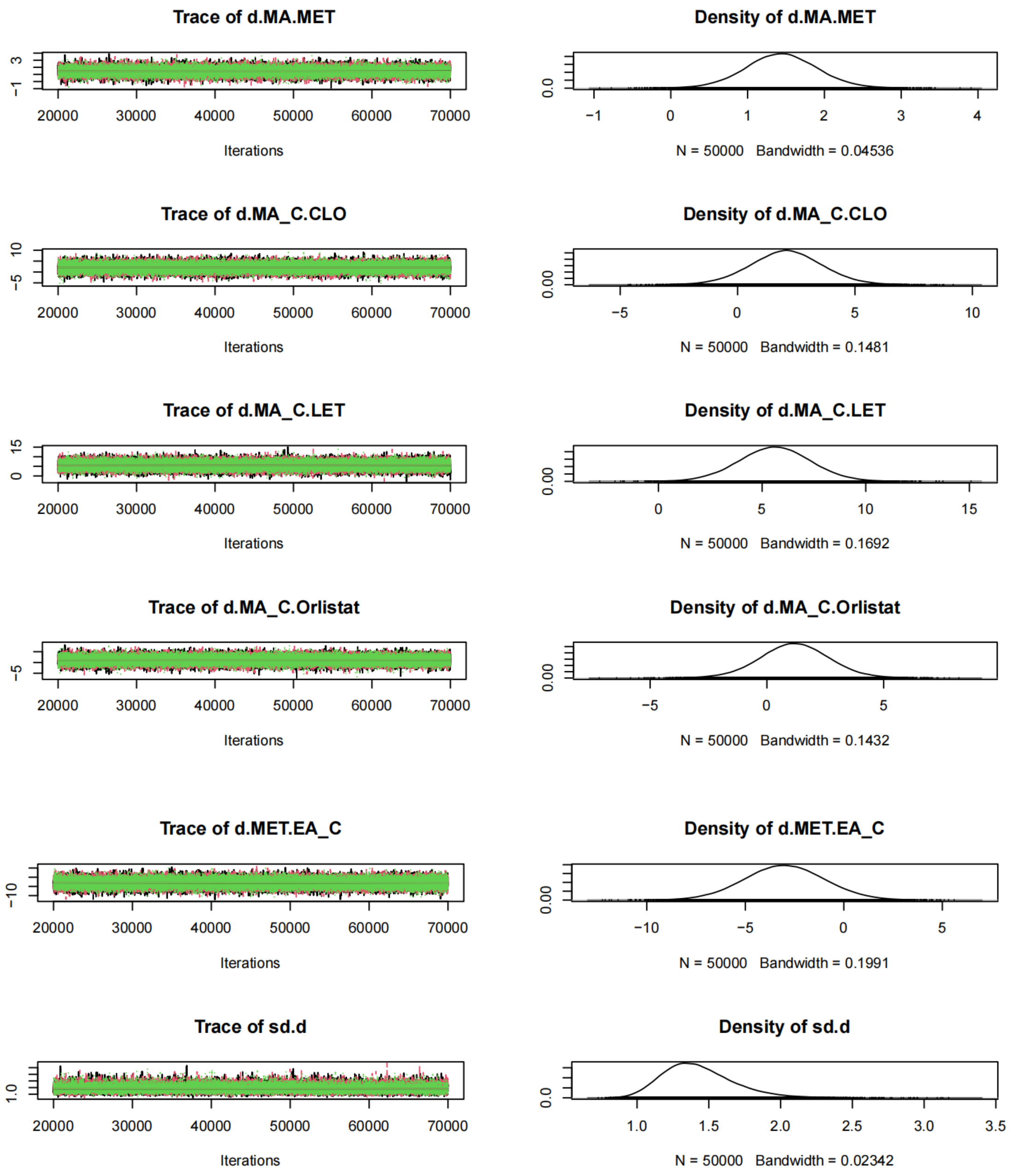
Trace of d.MA.MET
Iterations
Density of d.MA.MET
N = 50000 Bandwidth = 0.04536
Trace of d.MA_C.CLO
Iterations
Density of d.MA_C.CLO
N = 50000 Bandwidth = 0.1481
Trace of d.MA_C.LET
Iterations
Density of d.MA_C.LET
N = 50000 Bandwidth = 0.1692
Trace of d.MA_C.Orlistat
Iterations
Density of d.MA_C.Orlistat
N = 50000 Bandwidth = 0.1432
Trace of d.MET.EA_C
Iterations
Density of d.MET.EA_C
N = 50000 Bandwidth = 0.1991
Trace of sd.d
Iterations
Density of sd.d
N = 50000 Bandwidth = 0.02342

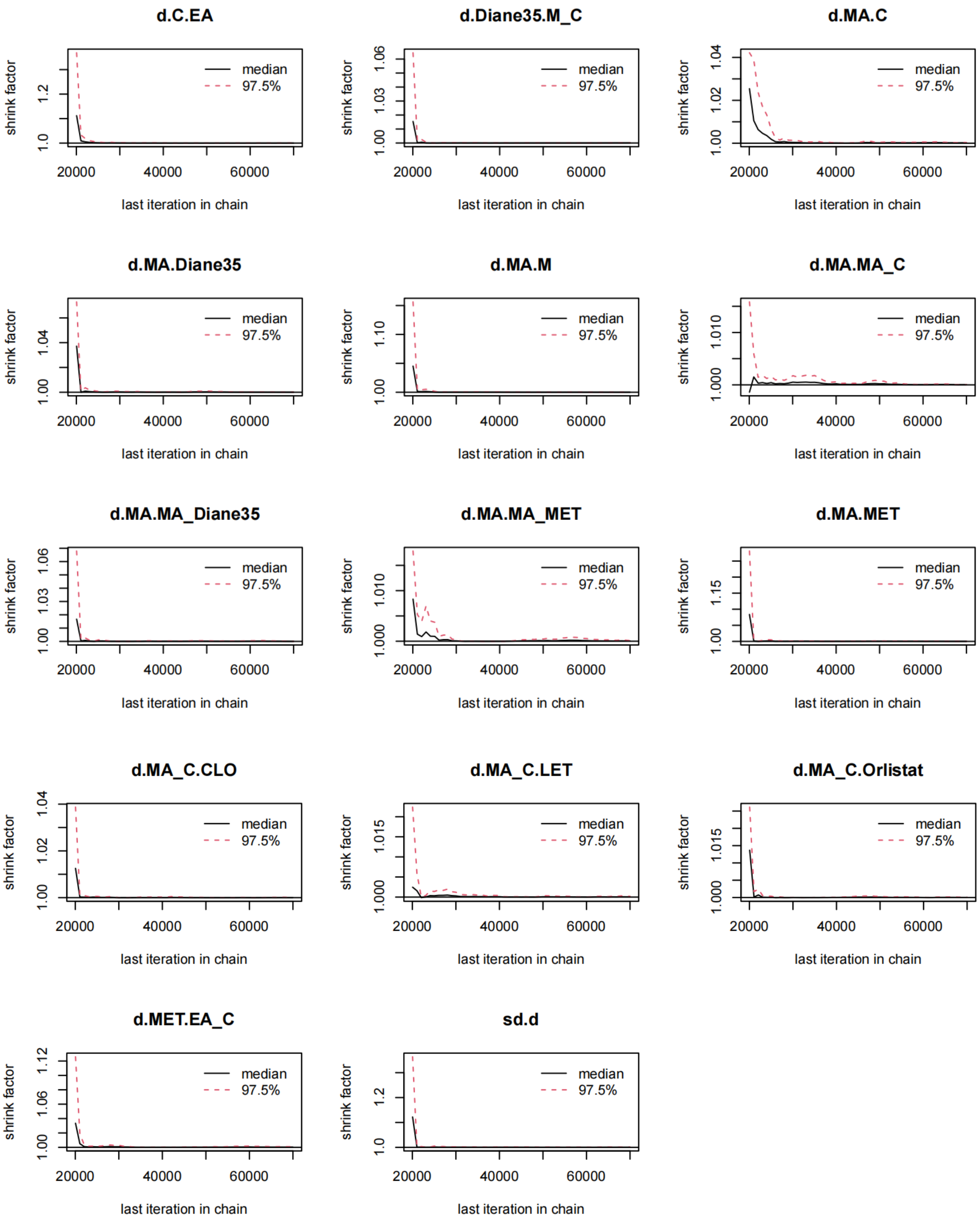
d.C.EA
d.Diane35.M_C
d.MA.C
d.MA.Diane35
d.MA.M
d.MA.MA_C
d.MA.MA_Diane35
d.MA.MA_MET
d.MA.MET
d.MA_C.CLO
d.MA_C.LET
d.MA_C.Orlistat
d.MET.EA_C
sd.d
median
97.5%
shrink factor
last iteration in chain
20000
40000
60000

02-The Trace plot, Density plot, and Gelman plot of WHR

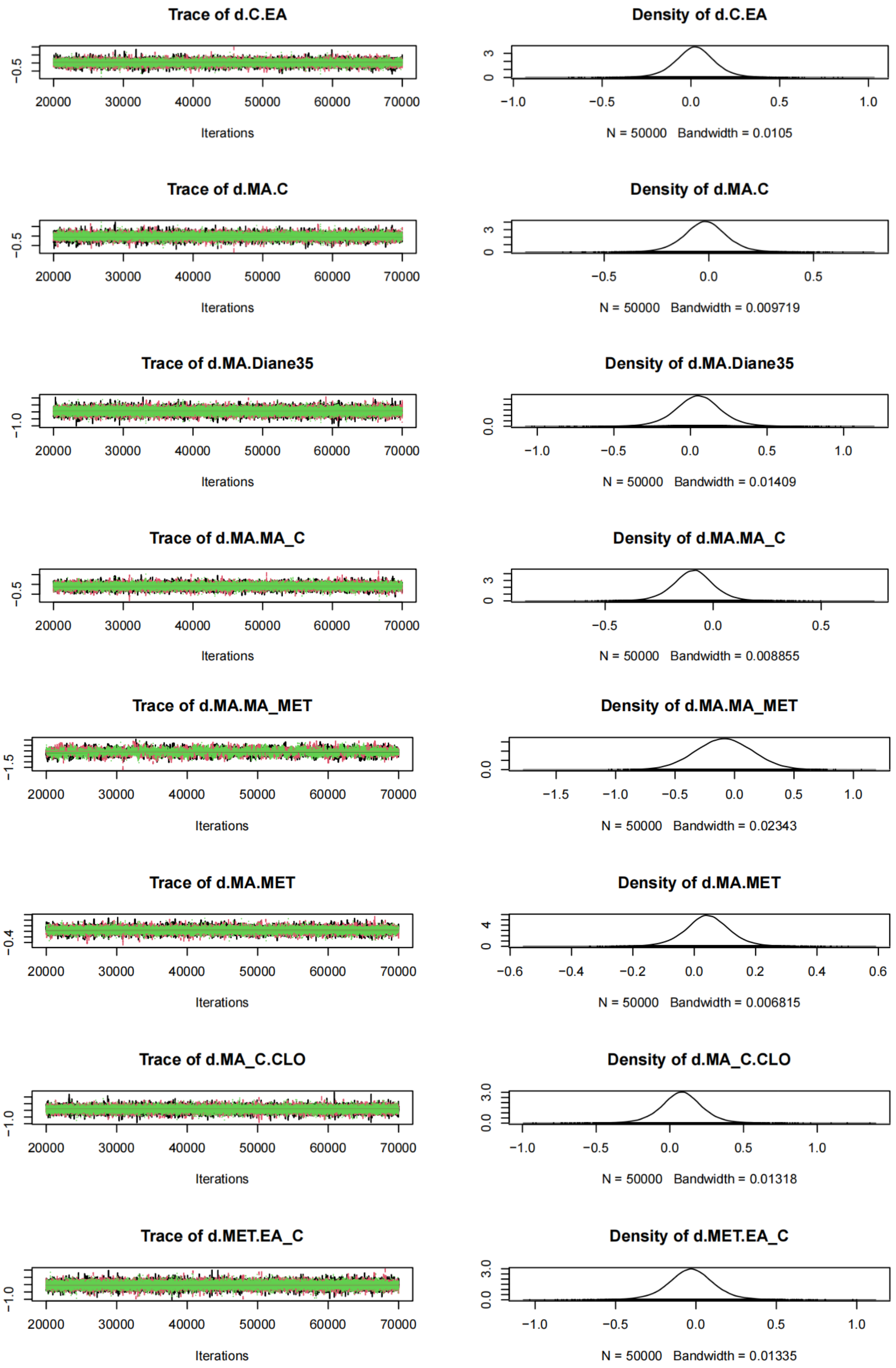

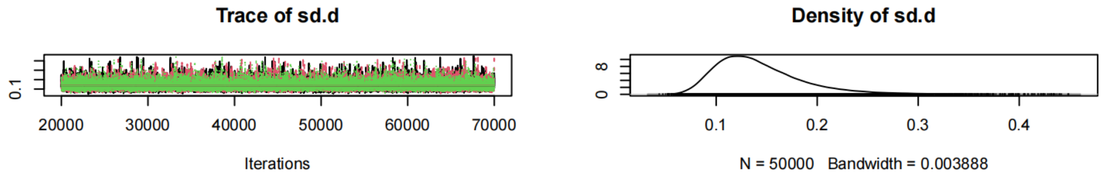

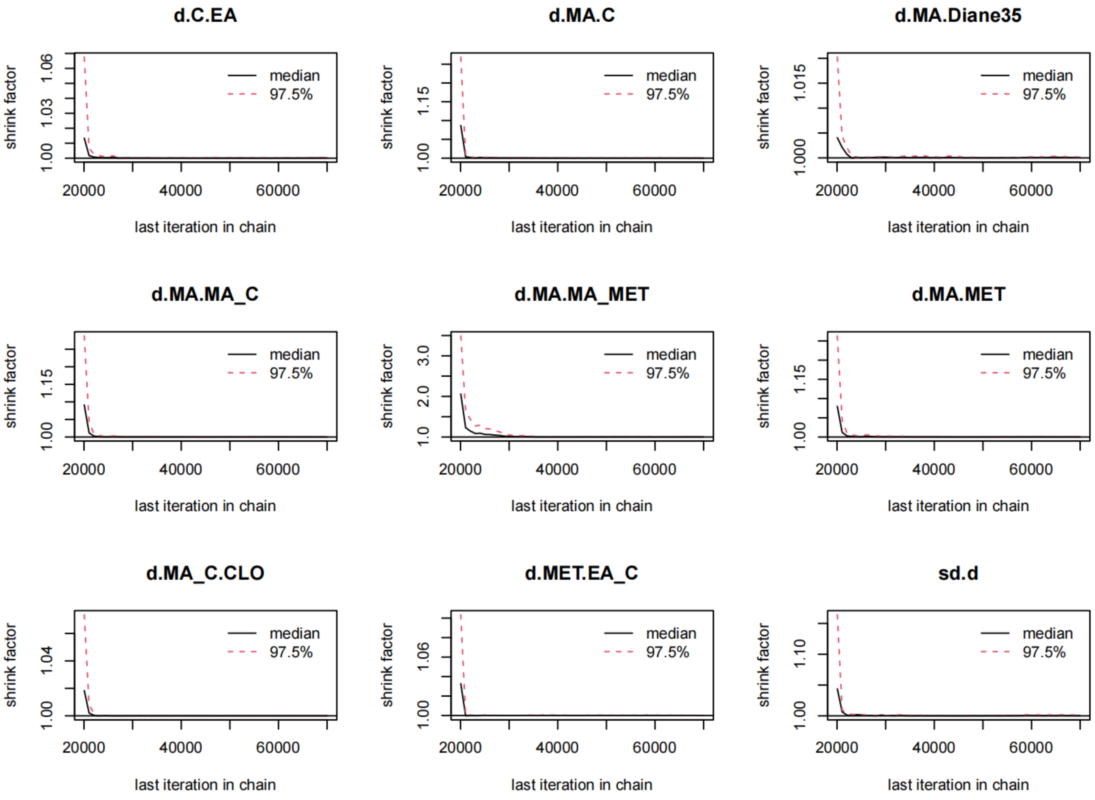

03-The Trace plot t, Density plot, and Gelman plot of BMI

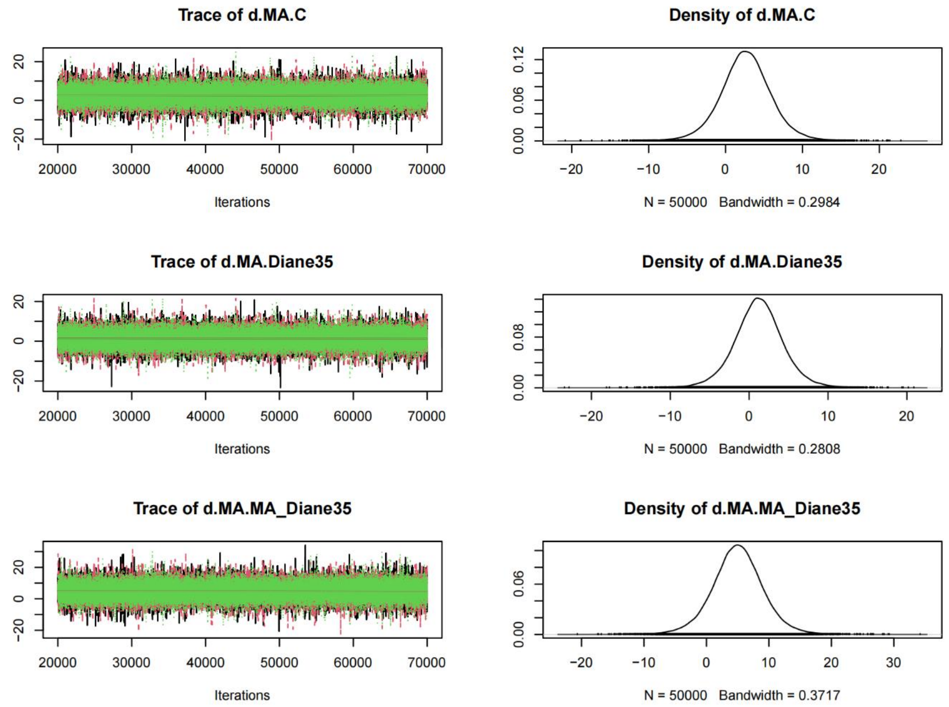

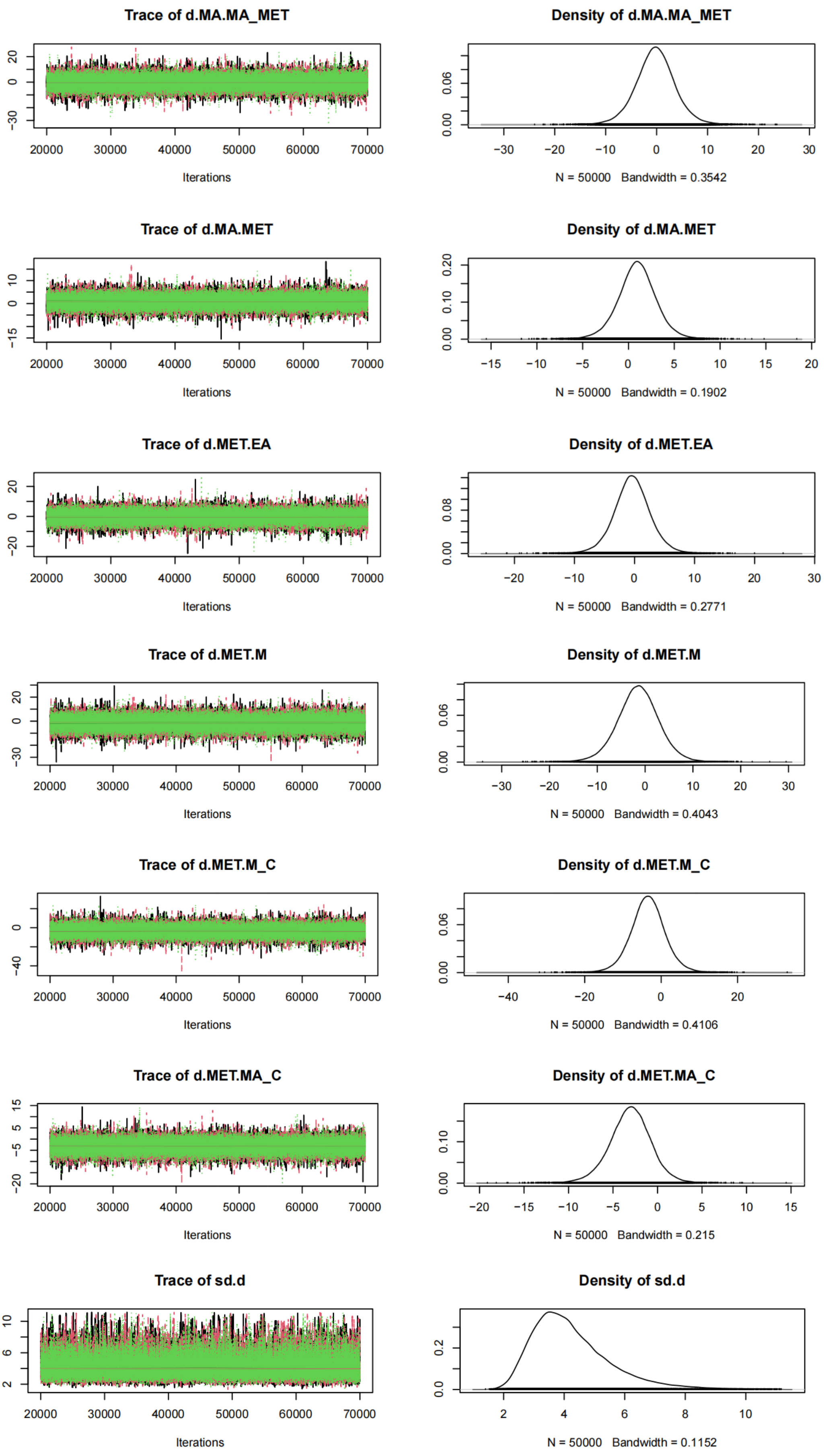
Trace of d.MA.MA_MET
Iterations
Density of d.MA.MA_MET
N = 50000 Bandwidth = 0.3542
Trace of d.MA.MET
Iterations
Density of d.MA.MET
N = 50000 Bandwidth = 0.1902
Trace of d.MET.EA
Iterations
Density of d.MET.EA
N = 50000 Bandwidth = 0.2771
Trace of d.MET.M
Iterations
Density of d.MET.M
N = 50000 Bandwidth = 0.4043
Trace of d.MET.M_C
Iterations
Density of d.MET.M_C
N = 50000 Bandwidth = 0.4106
Trace of d.MET.MA_C
Iterations
Density of d.MET.MA_C
N = 50000 Bandwidth = 0.215
Trace of sd.d
Iterations
Density of sd.d
N = 50000 Bandwidth = 0.1152

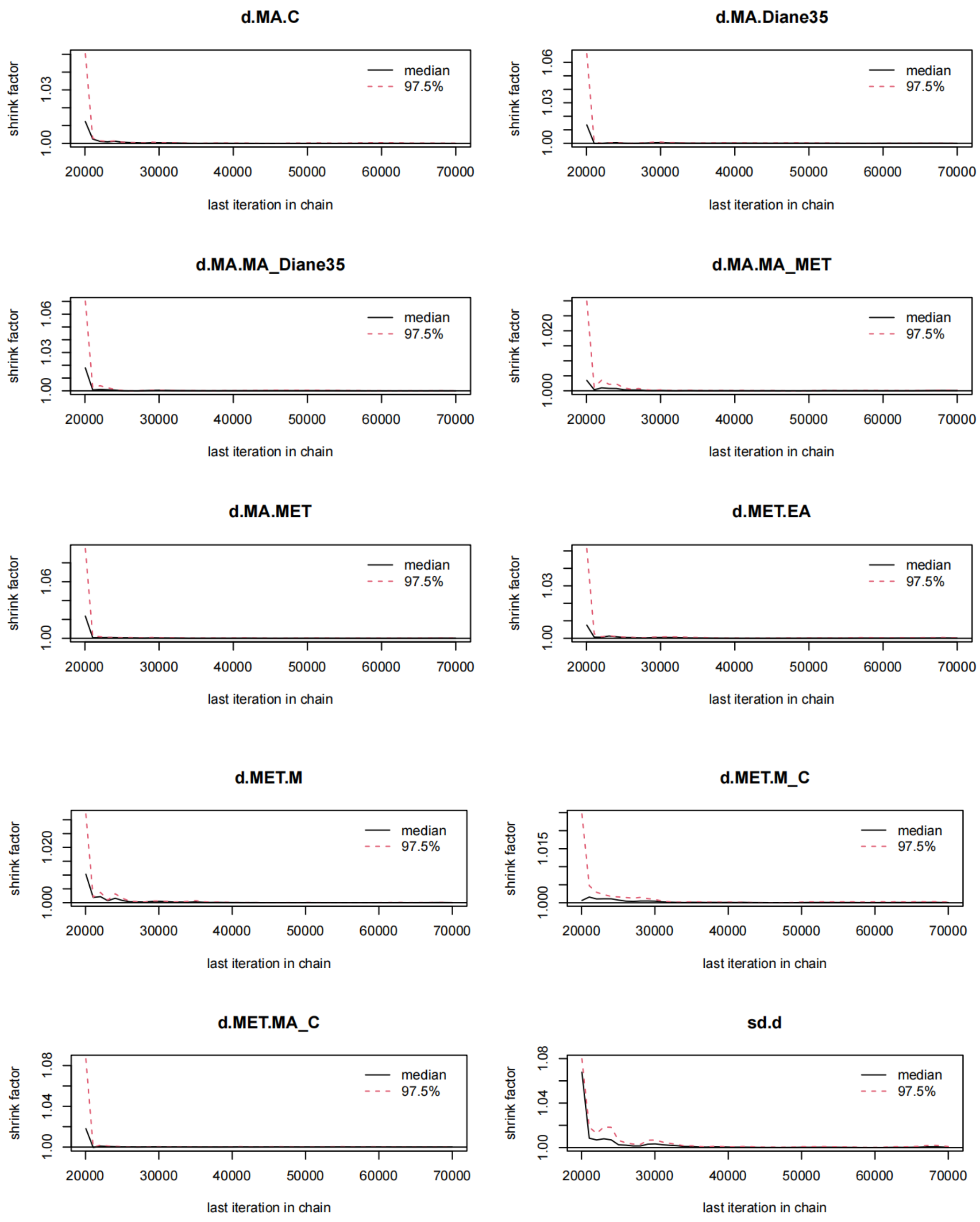
d.MA.C
d.MA.Diane35
d.MA.MA_Diane35
d.MA.MA_MET
d.MA.MET
d.MET.EA
d.MET.M
d.MET.M_C
d.MET.MA_C
sd.d
shrink factor
last iteration in chain
median
97.5%
20000 30000 40000 50000 60000 70000

04-The Trace plot t, Density plot, and Gelman plot of FPG

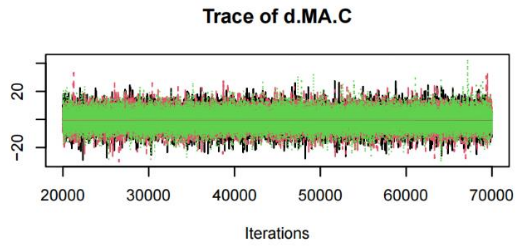

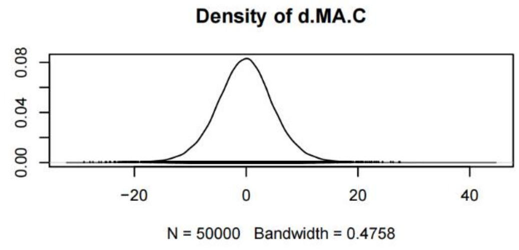

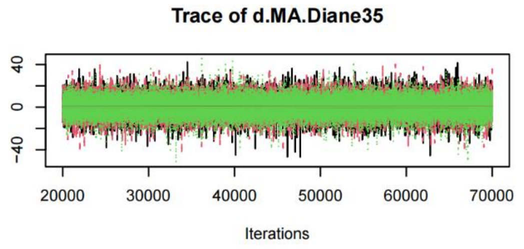

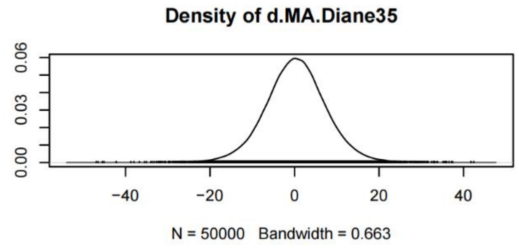

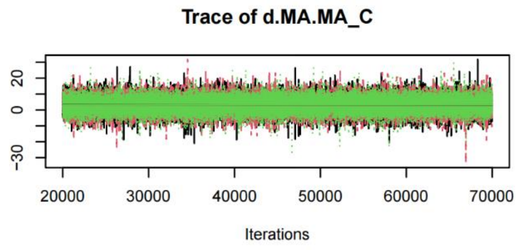

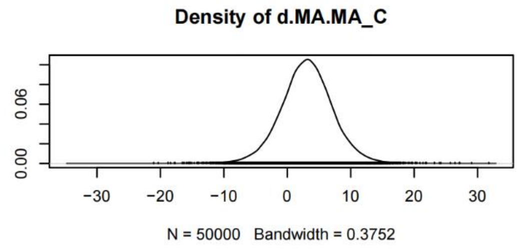

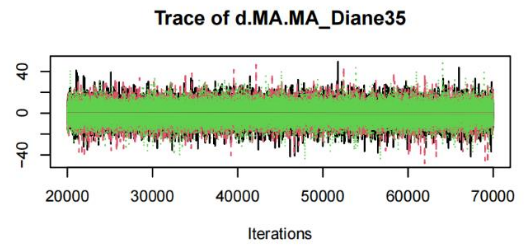

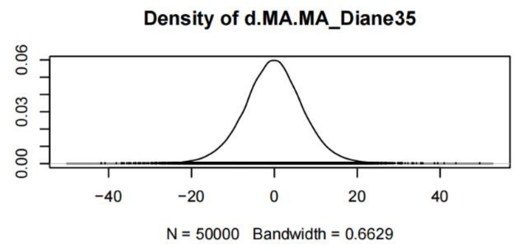

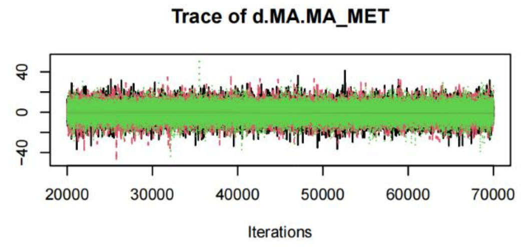

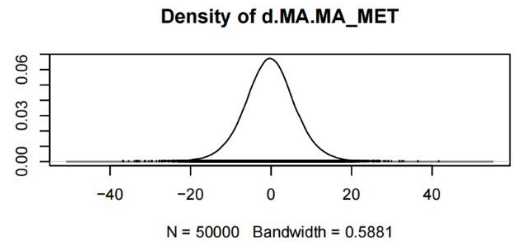

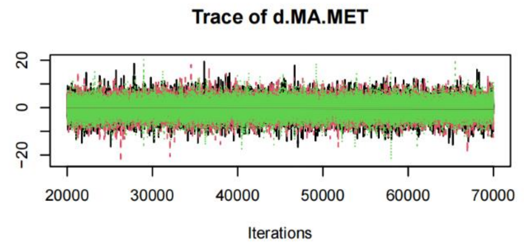

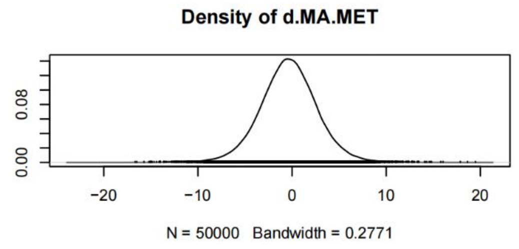

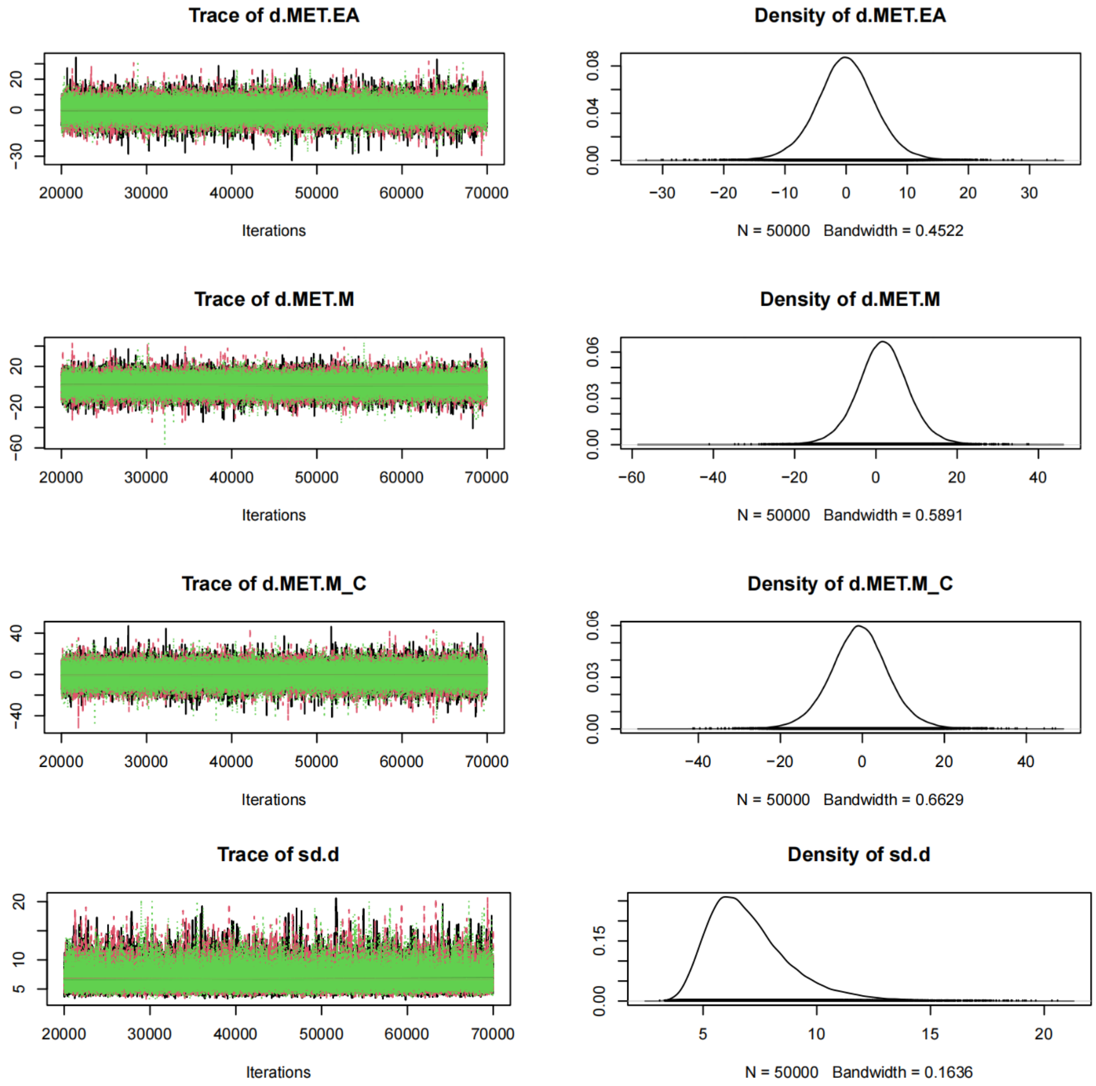
Trace of d.MET.EA
Iterations
Density of d.MET.EA
N = 50000 Bandwidth = 0.4522
Trace of d.MET.M
Iterations
Density of d.MET.M
N = 50000 Bandwidth = 0.5891
Trace of d.MET.M_C
Iterations
Density of d.MET.M_C
N = 50000 Bandwidth = 0.6629
Trace of sd.d
Iterations
Density of sd.d
N = 50000 Bandwidth = 0.1636

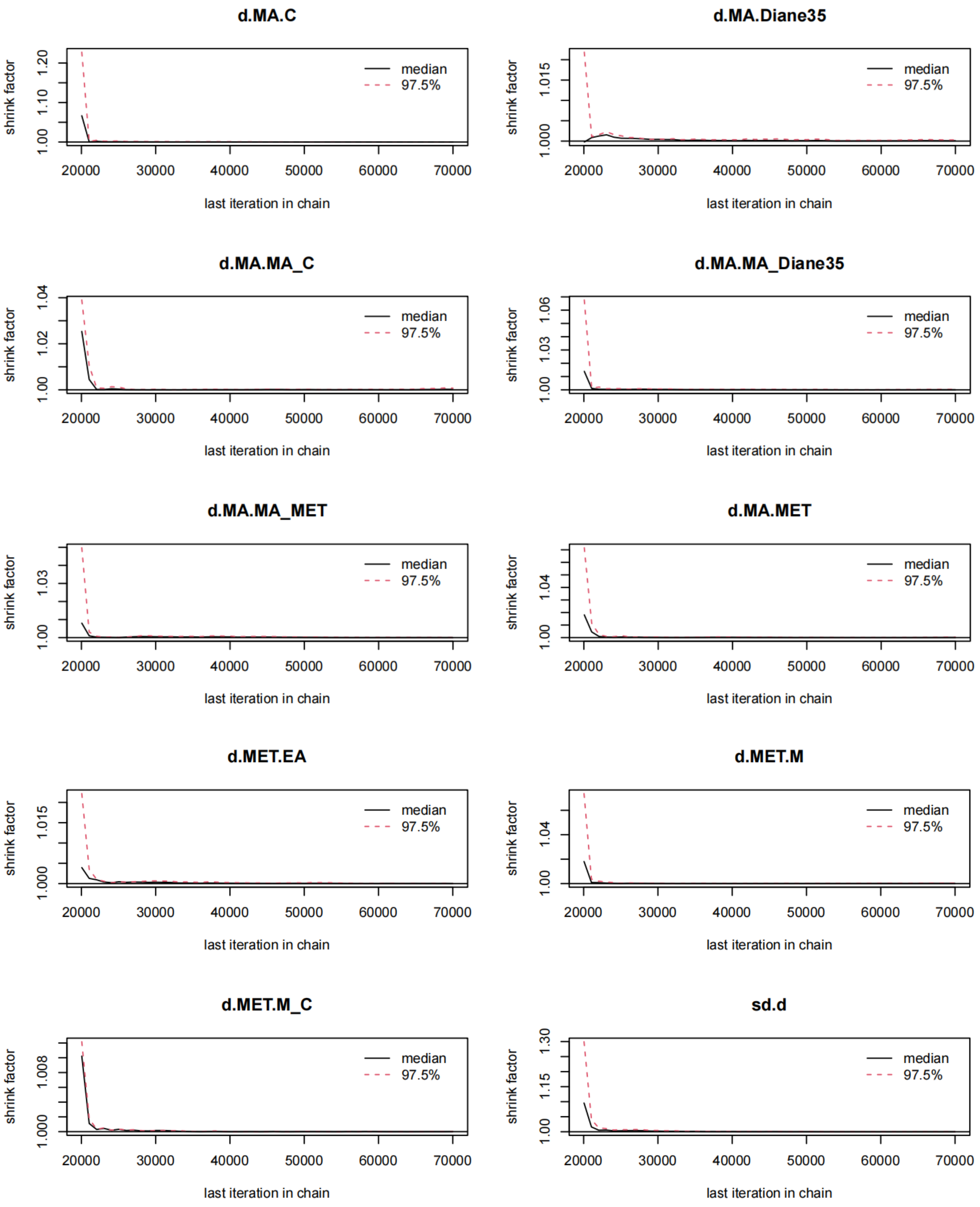
d.MA.C
shrink factor
1.00 1.10 1.20
median
97.5%
20000 30000 40000 50000 60000 70000
last iteration in chain
d.MA.Diane35
shrink factor
1.000 1.015
median
97.5%
20000 30000 40000 50000 60000 70000
last iteration in chain
d.MA.MA_C
shrink factor
1.00 1.02 1.04
median
97.5%
20000 30000 40000 50000 60000 70000
last iteration in chain
d.MA.MA_Diane35
shrink factor
1.00 1.03 1.06
median
97.5%
20000 30000 40000 50000 60000 70000
last iteration in chain
d.MA.MA_MET
shrink factor
1.00 1.03
median
97.5%
20000 30000 40000 50000 60000 70000
last iteration in chain
d.MA.MET
shrink factor
1.00 1.04
median
97.5%
20000 30000 40000 50000 60000 70000
last iteration in chain
d.MET.EA
shrink factor
1.000 1.015
median
97.5%
20000 30000 40000 50000 60000 70000
last iteration in chain
d.MET.M
shrink factor
1.00 1.04
median
97.5%
20000 30000 40000 50000 60000 70000
last iteration in chain
d.MET.M_C
shrink factor
1.000 1.008
median
97.5%
20000 30000 40000 50000 60000 70000
last iteration in chain
sd.d
shrink factor
1.00 1.15 1.30
median
97.5%
20000 30000 40000 50000 60000 70000
last iteration in chain

05-The Trace plot t, Density plot, and Gelman plot of IR

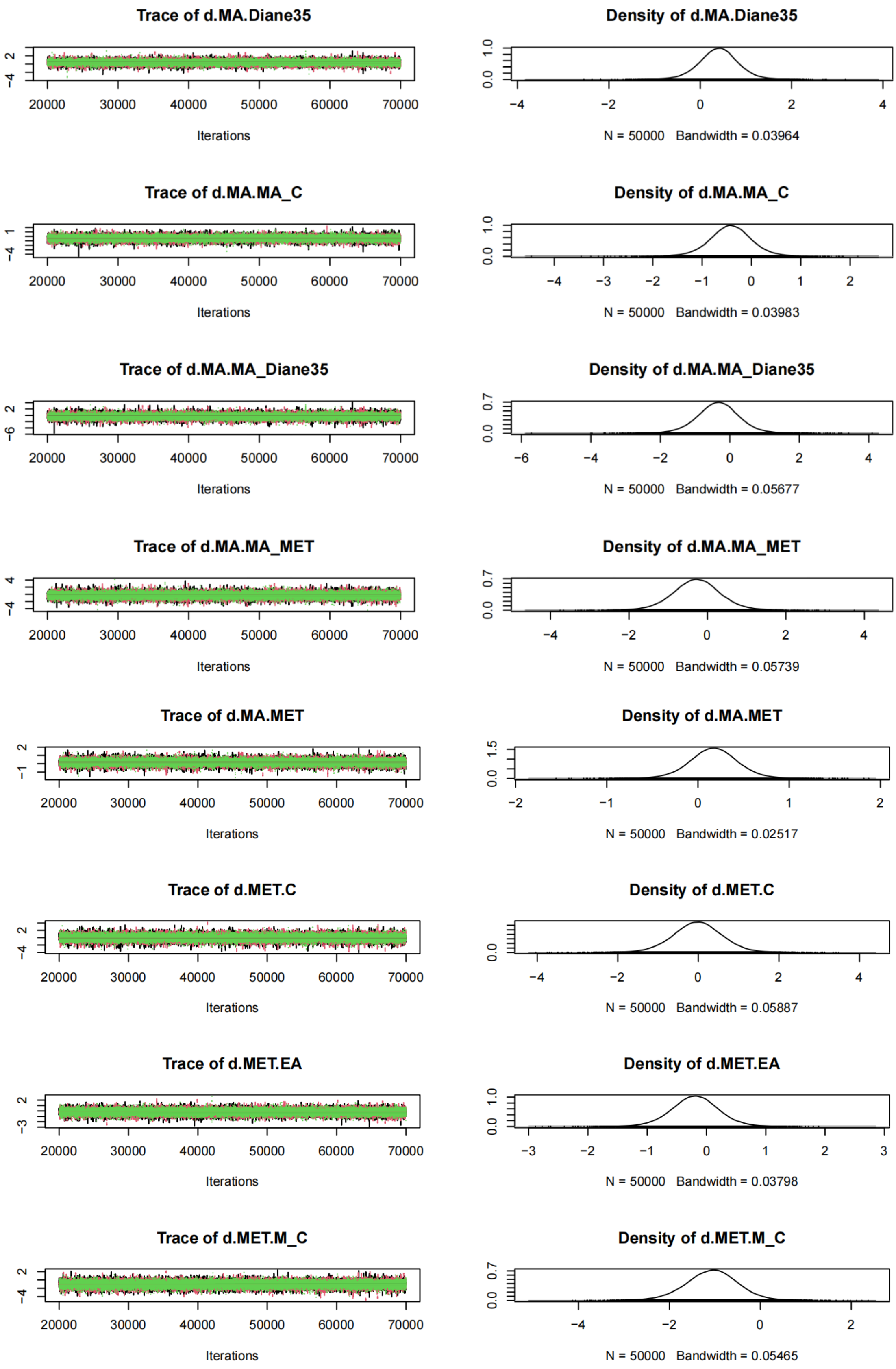

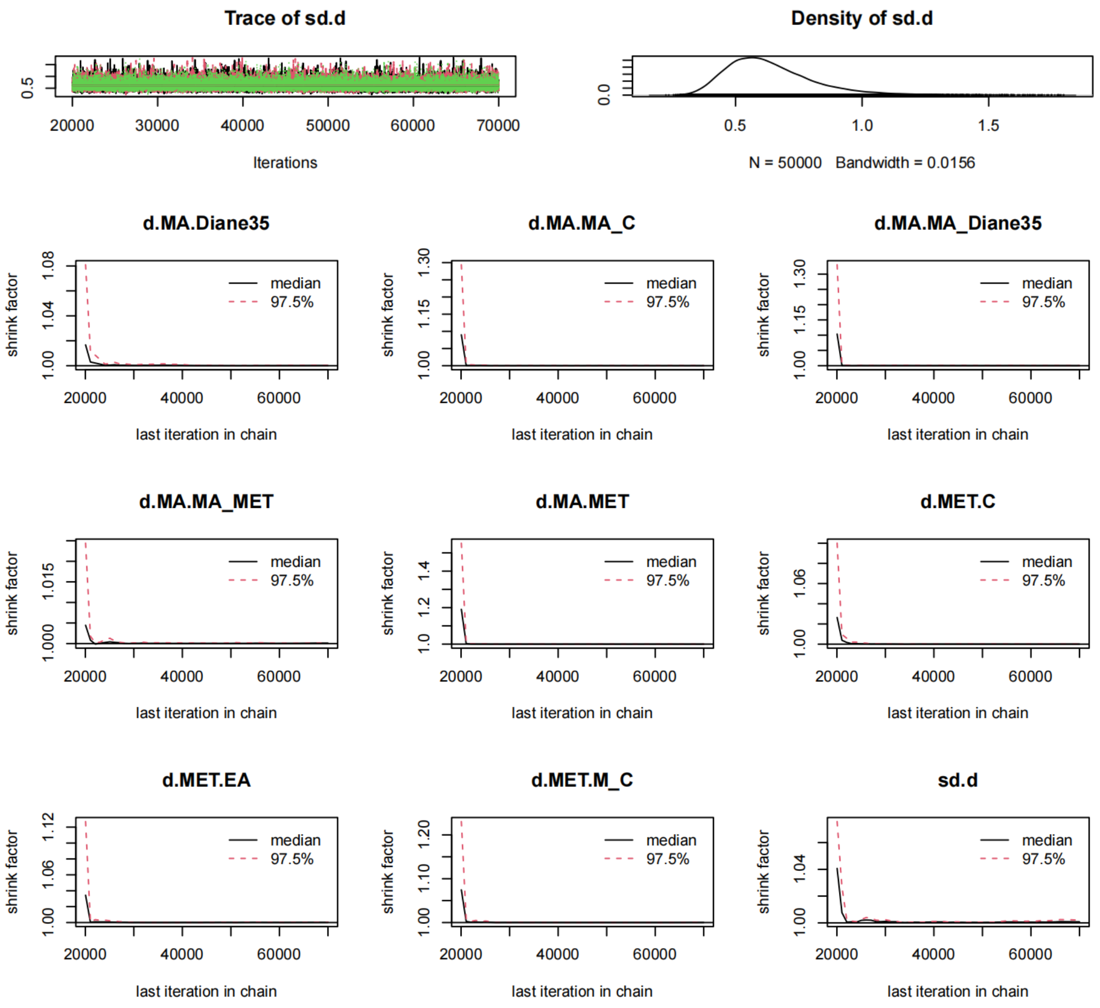
Trace of sd.d
0.5
20000
30000
40000
50000
60000
70000
Iterations
Density of sd.d
0.0
0.5
1.0
1.5
N = 50000 Bandwidth = 0.0156
d.MA.Diane35
d.MA.MA_C
d.MA.MA_Diane35
d.MA.MA_MET
d.MA.MET
d.MET.C
d.MET.EA
d.MET.M_C
sd.d
shrink factor
median
97.5%
last iteration in chain
20000
40000
60000

06-The Trace plot t, Density plot, and Gelman plot of TG

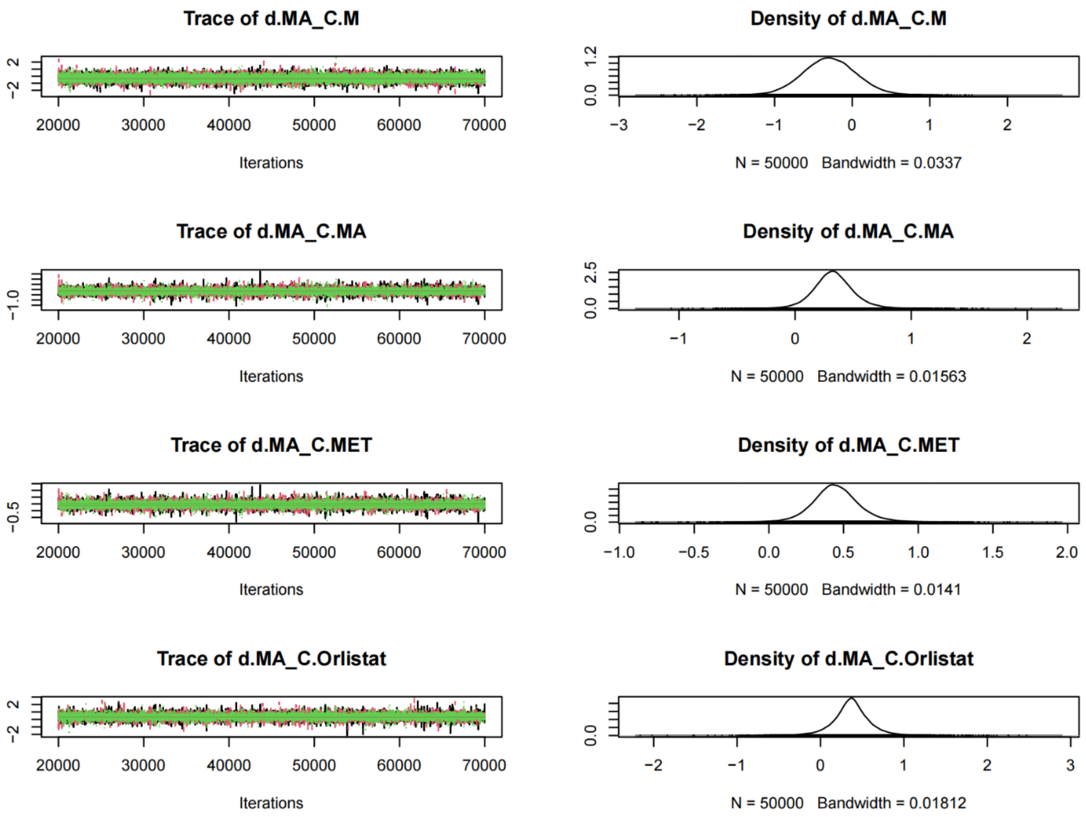

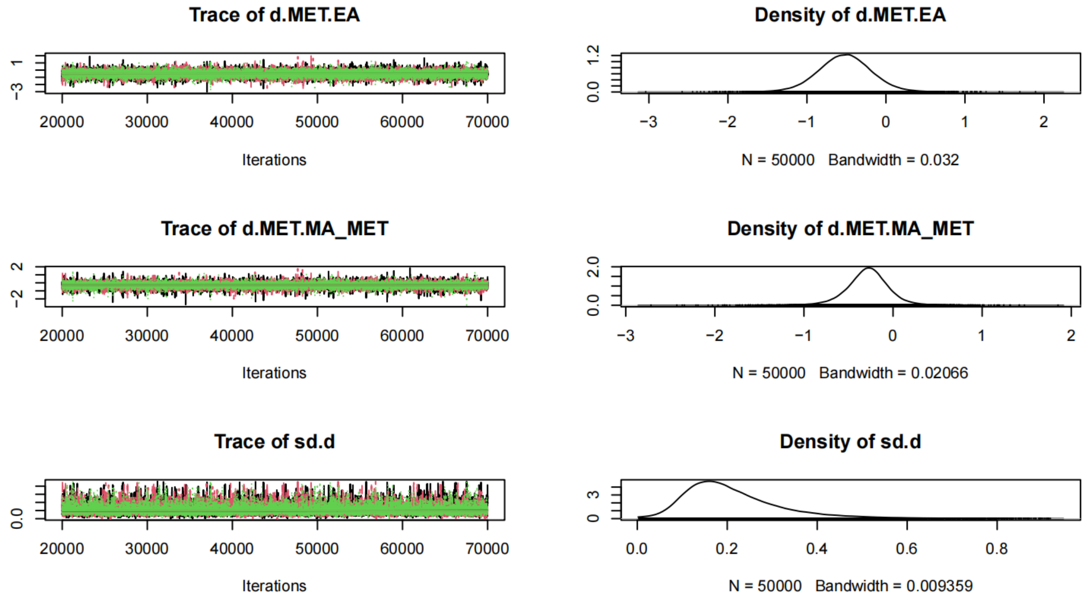

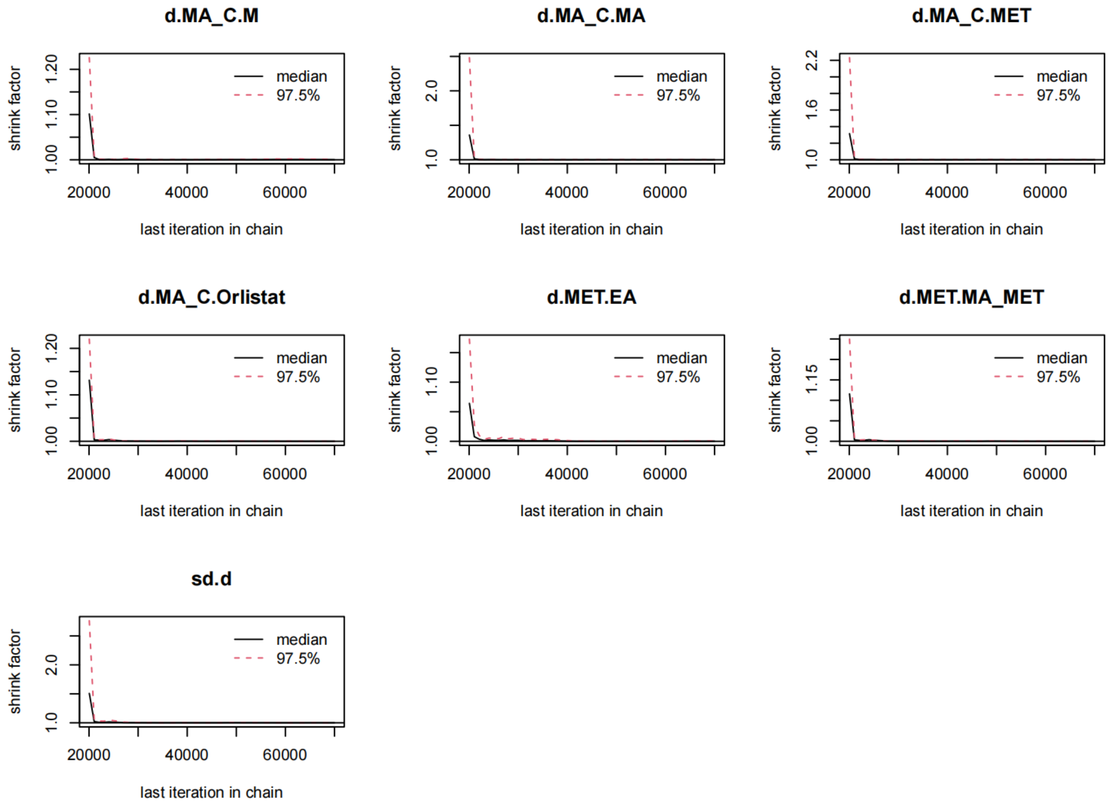
d.MA_C.M
d.MA_C.MA
d.MA_C.MET
d.MA_C.Orlistat
d.MET.EA
d.MET.MA_MET
sd.d
shrink factor
last iteration in chain
median
97.5%
20000
40000
60000
1.00
1.10
1.20
1.0
1.6
2.0
2.2
1.15

S6. Network meta-analysis of BMI at the end of treatment

**MA**													
0.18 (-1.02, 1.41)	**MA+C**												
0.72 (-0.99, 2.41)	0.53 (-1.37, 2.41)	**MA+MET**											
2.4 (-0.5, 5.26)	2.21 (-0.85, 5.23)	1.68 (-1.62, 5)	**MA+Diane35**										
0.48 (-1.67, 2.62)	0.3 (-2.04, 2.61)	-0.24 (-2.85, 2.42)	-1.91 (-5.47, 1.66)	**C**									
-1.89 (-5.18, 1.41)	-2.08 (-5.14, 1)	-2.61 (-6.18, 1.02)	-4.29 (-8.56, 0.04)	-2.37 (-6.2, 1.47)	**CLO**								
**-1.66 (-3.15, -0.19)**	**-1.84 (-3.5, -0.22)**	**-2.37 (-4.54, -0.22)**	**-4.05 (-6.9, -1.19)**	-2.14 (-4.7, 0.4)	0.24 (-3.27, 3.68)	**Diane-35**							
-0.57 (-2.52, 1.38)	-0.76 (-2.81, 1.29)	-1.29 (-3.66, 1.12)	-2.97 (-6.4, 0.48)	-1.06 (-3.46, 1.37)	1.32 (-2.38, 5)	1.08 (-1.23, 3.45)	**EA**						
1.57 (-2.57, 5.68)	1.39 (-2.8, 5.52)	0.86 (-3.48, 5.16)	-0.82 (-5.85, 4.18)	1.09 (-3.47, 5.62)	3.46 (-1.72, 8.6)	3.23 (-1.09, 7.53)	2.15 (-2.27, 6.52)	**EA+C**					
**-5.43 (-9.08, -1.76)**	**-5.61 (-9.06, -2.16)**	**-6.14 (-10.1, -2.2)**	**-7.82 (-12.4, -3.18)**	**-5.91 (-10.06, -1.74)**	-3.53 (-8.18, 1.09)	-3.76 (-7.56, 0.05)	**-4.85 (-8.85, -0.87)**	**-6.99 (-12.37, -1.57)**	**LET**				
**-1.44 (-2.39, -0.51)**	**-1.63 (-2.69, -0.59)**	**-2.15 (-3.78, -0.54)**	**-3.84 (-6.8, -0.86)**	-1.92 (-4.07, 0.21)	0.45 (-2.81, 3.68)	0.22 (-1.34, 1.8)	-0.86 (-2.65, 0.88)	-3.01 (-7.01, 1.01)	**3.99 (0.36, 7.58)**	**MET**			
-0.55 (-2.56, 1.46)	-0.73 (-2.81, 1.34)	-1.26 (-3.77, 1.28)	-2.95 (-6.31, 0.45)	-1.03 (-3.88, 1.85)	1.34 (-2.37, 5.04)	1.11 (-1.03, 3.29)	0.03 (-2.66, 2.69)	-2.12 (-6.6, 2.39)	**4.88 (0.86, 8.88)**	0.89 (-1.12, 2.92)	**M**		
-0.54 (-2.86, 1.76)	-0.73 (-3.13, 1.64)	-1.26 (-3.99, 1.48)	-2.94 (-6.43, 0.58)	-1.02 (-4.09, 2.02)	1.35 (-2.55, 5.2)	1.12 (-1.12, 3.36)	0.03 (-2.83, 2.86)	-2.12 (-6.71, 2.48)	**4.88 (0.68, 9.06)**	0.9 (-1.34, 3.15)	0.01 (-2.87, 2.85)	**M+C**	
-1 (-4.22, 2.22)	-1.19 (-4.17, 1.79)	-1.72 (-5.23, 1.83)	-3.4 (-7.63, 0.87)	-1.49 (-5.25, 2.3)	0.89 (-3.39, 5.14)	0.66 (-2.74, 4.07)	-0.43 (-4.04, 3.19)	-2.57 (-7.69, 2.57)	4.42 (-0.14, 8.99)	0.44 (-2.71, 3.63)	-0.46 (-4.09, 3.15)	-0.46 (-4.25, 3.36)	**Orlistat**

S7.Network meta-analysis of WHR at the end of treatment

**C**								
-0.01 (-0.4, 0.39)	**CLO**							
-0.07 (-0.45, 0.32)	-0.06 (-0.54, 0.41)	**Diane-35**						
-0.02 (-0.26, 0.22)	-0.02 (-0.4, 0.36)	0.04 (-0.34, 0.43)	**EA**					
-0.03 (-0.4, 0.34)	-0.02 (-0.48, 0.43)	0.04 (-0.42, 0.5)	-0.01 (-0.36, 0.34)	**EA+C**				
-0.02 (-0.24, 0.2)	-0.01 (-0.37, 0.35)	0.05 (-0.26, 0.36)	0.01 (-0.21, 0.23)	0.01 (-0.33, 0.35)	**MA**			
0.07 (-0.18, 0.33)	0.08 (-0.22, 0.38)	0.14 (-0.23, 0.51)	0.1 (-0.13, 0.33)	0.1 (-0.23, 0.45)	0.09 (-0.1, 0.29)	**MA+C**		
0.07 (-0.43, 0.57)	0.07 (-0.49, 0.64)	0.14 (-0.43, 0.7)	0.09 (-0.39, 0.58)	0.1 (-0.45, 0.64)	0.09 (-0.38, 0.56)	-0.01 (-0.49, 0.47)	**MA+MET**	
-0.06 (-0.27, 0.16)	-0.06 (-0.39, 0.28)	0.01 (-0.34, 0.36)	-0.04 (-0.21, 0.13)	-0.03 (-0.33, 0.27)	-0.04 (-0.19, 0.11)	-0.13 (-0.29, 0.02)	-0.13 (-0.58, 0.33)	**MET**

S8.Network meta-analysis of FIN at the end of treatment

**C**									
1.41 (-7.77, 10.68)	**Diane35**								
2.08 (-5.45, 9.74)	0.65 (-8.99, 10.34)	**EA**							
3.11 (-7.81, 14.24)	1.67 (-9.89, 13.4)	1.01 (-9.65, 11.93)	**M**						
5.14 (-6.16, 16.39)	3.73 (-8.24, 15.69)	3.06 (-8.03, 14.2)	2.05 (-10.76, 14.65)	**M+C**					
2.64 (-4.05, 9.42)	1.21 (-5.19, 7.64)	0.56 (-6.67, 7.86)	-0.46 (-10.38, 9.24)	-2.51 (-12.58, 7.65)	**MA**				
4.72 (-3.17, 13.05)	3.28 (-5.57, 12.52)	2.61 (-4.96, 10.66)	1.61 (-7.18, 10.5)	-0.43 (-10.53, 10.09)	2.07 (-4.13, 8.65)	**MA+C**			
-2.36 (-13.09, 8.47)	-3.79 (-12.19, 4.63)	-4.43 (-15.58, 6.76)	-5.48 (-18.37, 7.37)	-7.49 (-20.69, 5.6)	-5 (-13.4, 3.44)	-7.06 (-17.78, 3.27)	**MA+Diane35**		
2.87 (-7.03, 12.9)	1.44 (-8.86, 11.75)	0.78 (-9.28, 10.89)	-0.22 (-12.24, 11.54)	-2.27 (-14.52, 9.94)	0.22 (-7.82, 8.25)	-1.85 (-11.37, 7.3)	5.24 (-6.49, 16.83)	**MA+MET**	
1.68 (-4.83, 8.31)	0.27 (-7.41, 8.01)	-0.4 (-6.61, 5.86)	-1.41 (-10.31, 7.32)	-3.46 (-12.64, 5.74)	-0.95 (-5.24, 3.37)	-3.02 (-7.95, 1.55)	4.05 (-5.4, 13.51)	-1.19 (-9.18, 6.88)	**MET**

S9.Network meta-analysis of IR at the end of treatment

**C**								
-0.25 (-1.84, 1.35)	**Diane-35**							
0.18 (-1.11, 1.45)	0.42 (-0.86, 1.69)	**EA**						
1.04 (-0.65, 2.8)	**1.29 (0.22, 2.42)**	0.87 (-0.52, 2.33)	**M+C**					
0.15 (-1.23, 1.53)	0.4 (-0.47, 1.27)	-0.02 (-1, 0.96)	-0.89 (-2.1, 0.25)	**MA**				
0.57 (-0.91, 2.07)	0.82 (-0.37, 2.01)	0.4 (-0.7, 1.52)	-0.46 (-1.86, 0.87)	0.42 (-0.44, 1.3)	**MA+C**			
0.49 (-1.35, 2.34)	0.74 (-0.53, 2)	0.31 (-1.24, 1.89)	-0.55 (-2.16, 1)	0.33 (-0.91, 1.59)	-0.09 (-1.59, 1.43)	**MA+Diane35**		
0.43 (-1.36, 2.21)	0.68 (-0.84, 2.17)	0.26 (-1.25, 1.75)	-0.61 (-2.32, 1.03)	0.28 (-0.98, 1.53)	-0.15 (-1.61, 1.29)	-0.06 (-1.84, 1.69)	**MA+MET**	
-0.02 (-1.3, 1.24)	0.23 (-0.75, 1.19)	-0.19 (-1.01, 0.62)	-1.06 (-2.26, 0.07)	-0.17 (-0.72, 0.37)	-0.59 (-1.36, 0.16)	-0.51 (-1.86, 0.82)	-0.45 (-1.71, 0.81)	**MET**

S10. Ranking for BMI (Ranking indicates the probability to be the best treatment, the second best, the third best and so on, among interventions).

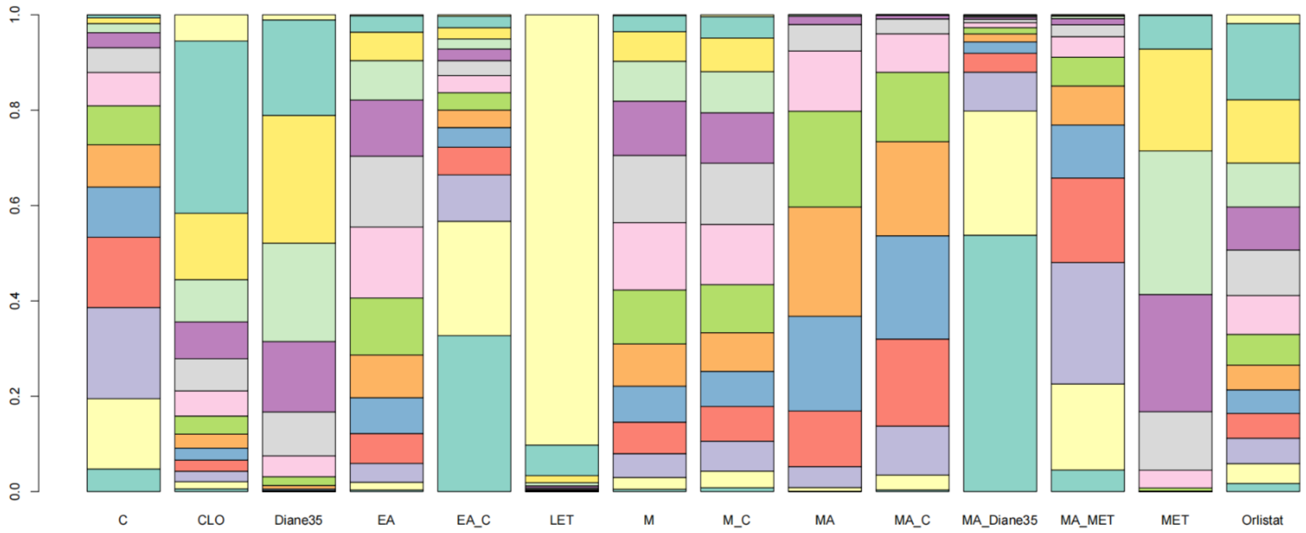

S11. Ranking for WHR (Ranking indicates the probability to be the best treatment, the second best, the third best and so on, among interventions).

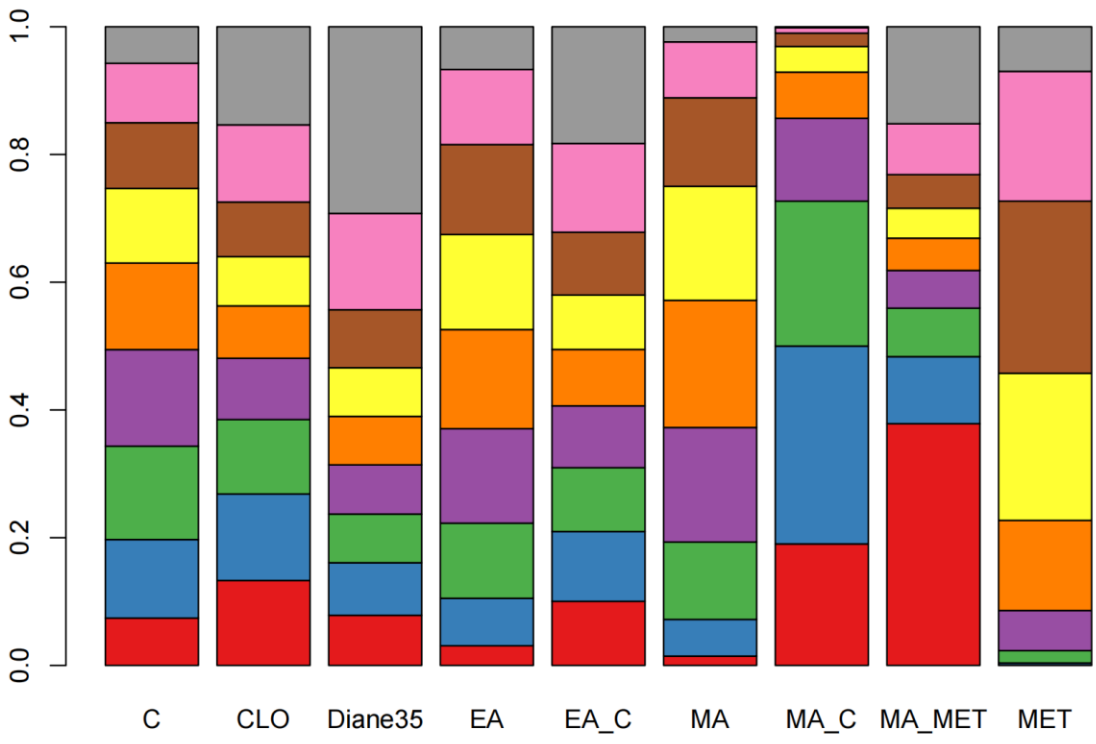

S12. Ranking for FIN (Ranking indicates the probability to be the best treatment, the second best, the third best and so on, among interventions).

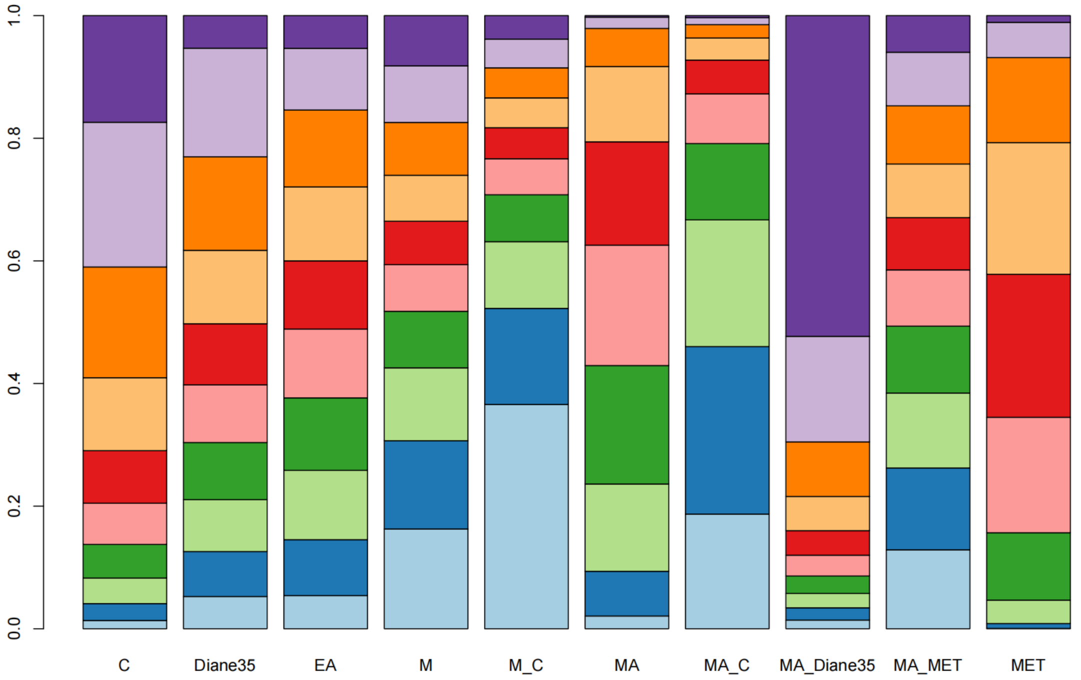

S13. Ranking for FPG (Ranking indicates the probability to be the best treatment, the second best, the third best and so on, among interventions).

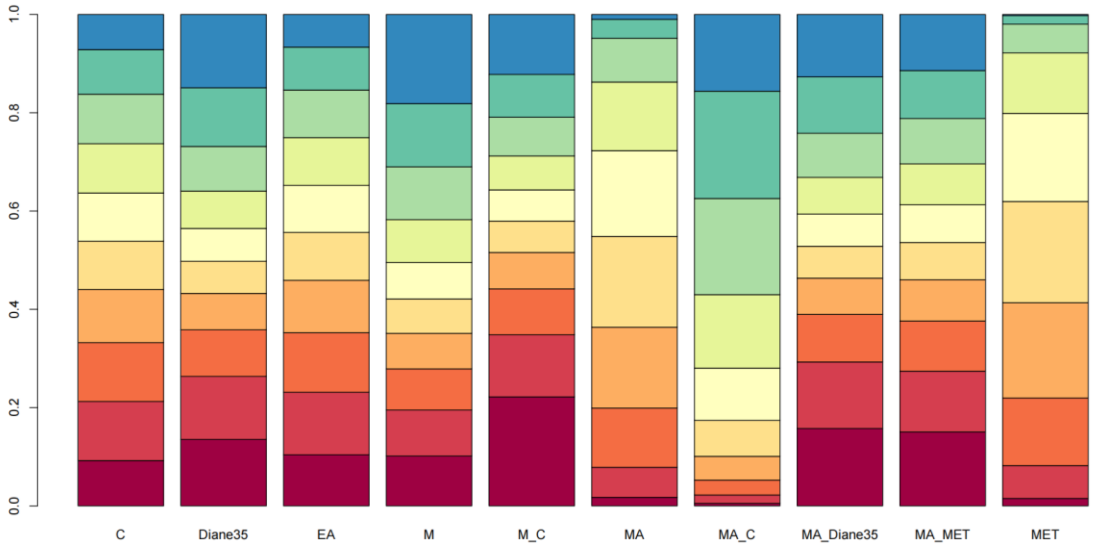

S14. Ranking for IR (Ranking indicates the probability to be the best treatment, the second best, the third best and so on, among interventions).

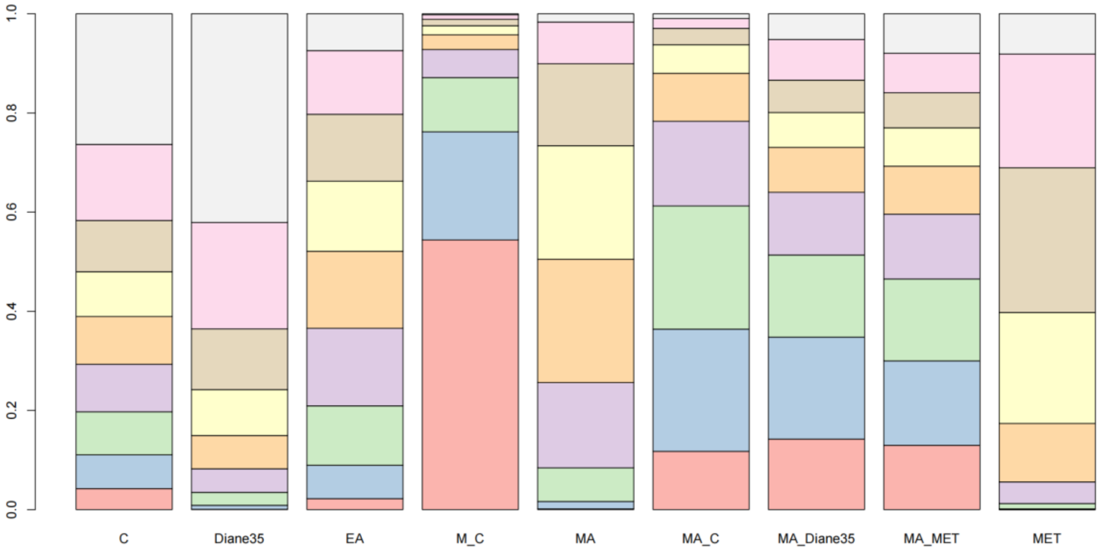

S15. Ranking for TG (Ranking indicates the probability to be the best treatment, the second best, the third best and so on, among interventions).

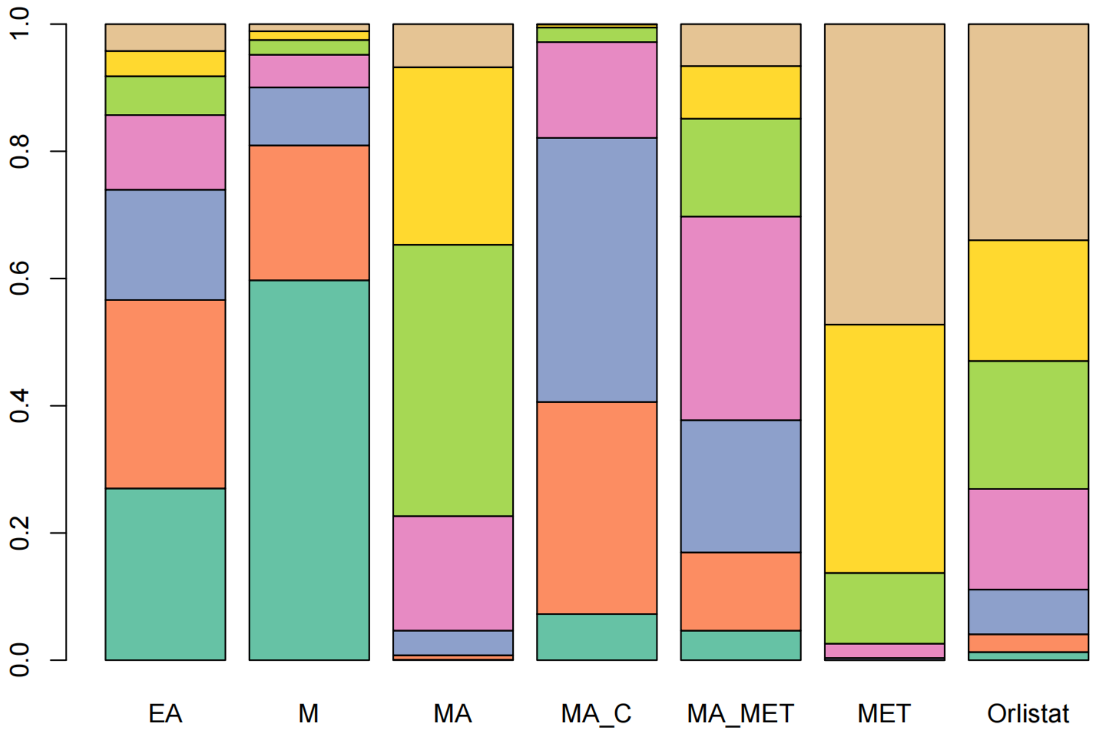

S16. The node-splitting analysis

S13-01 The node-splitting analysis of BMI

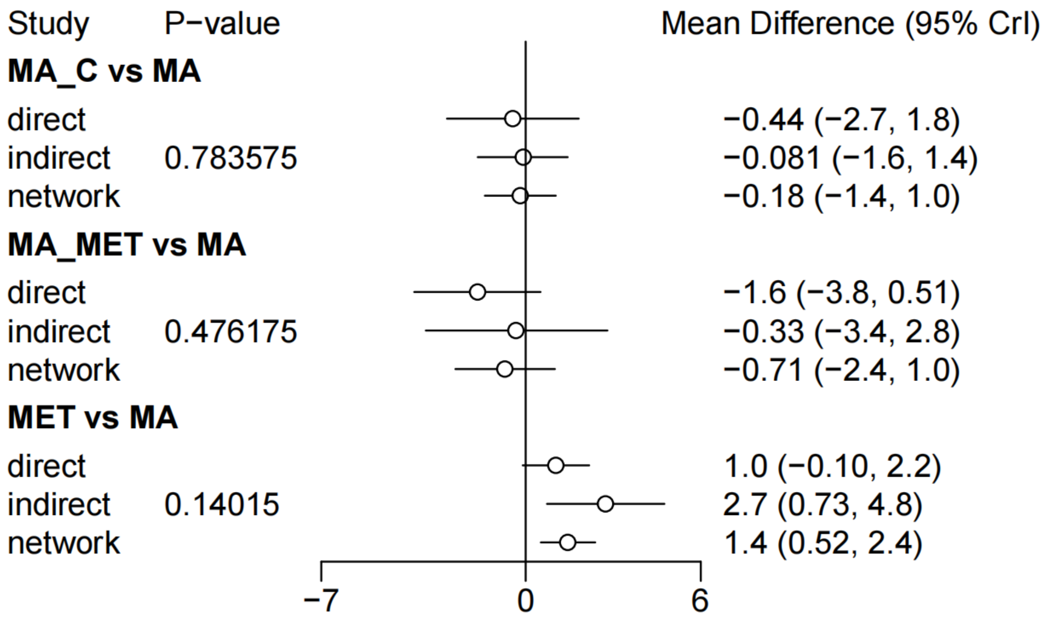

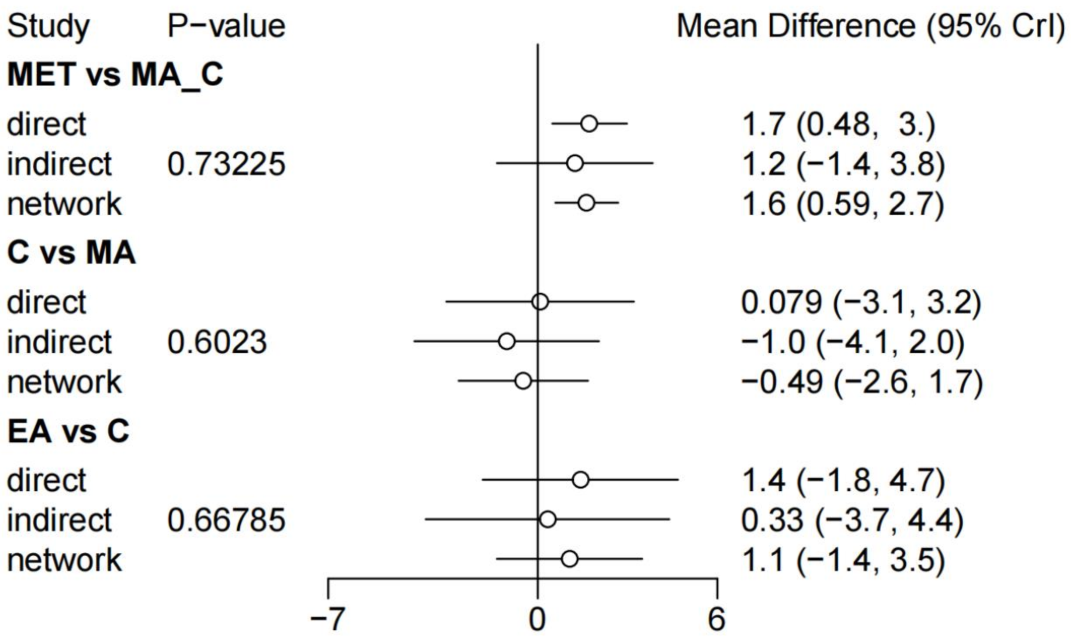

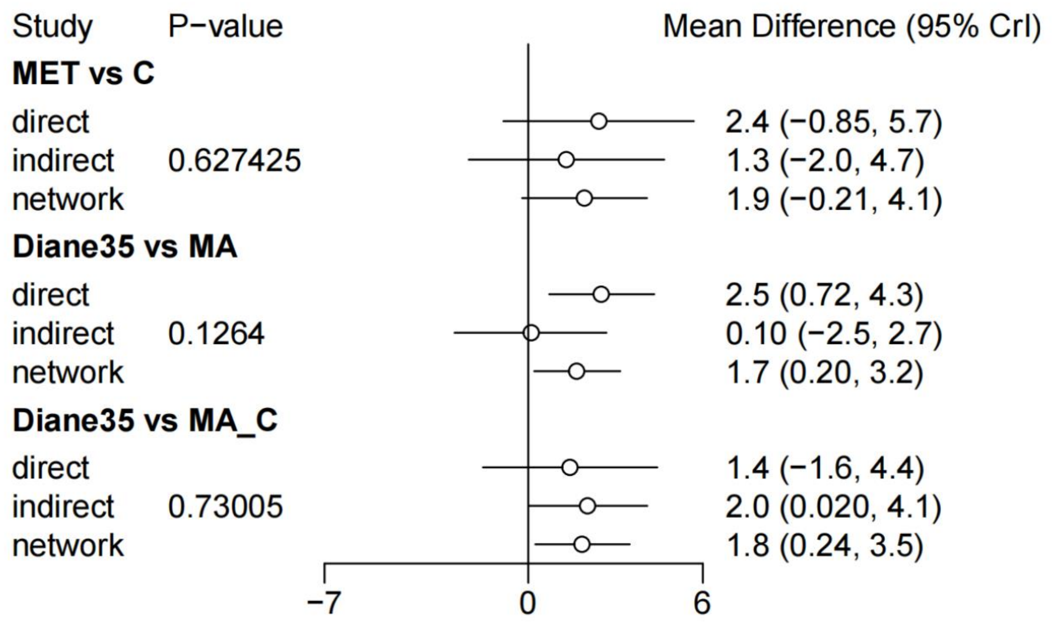
Study
P−value
Mean Difference (95% CrI)
MET vs C
direct
2.4 (−0.85, 5.7)
indirect
0.627425
1.3 (−2.0, 4.7)
network
1.9 (−0.21, 4.1)
Diane35 vs MA
direct
2.5 (0.72, 4.3)
indirect
0.1264
0.10 (−2.5, 2.7)
network
1.7 (0.20, 3.2)
Diane35 vs MA_C
direct
1.4 (−1.6, 4.4)
indirect
0.73005
2.0 (0.020, 4.1)
network
1.8 (0.24, 3.5)
−7
0
6

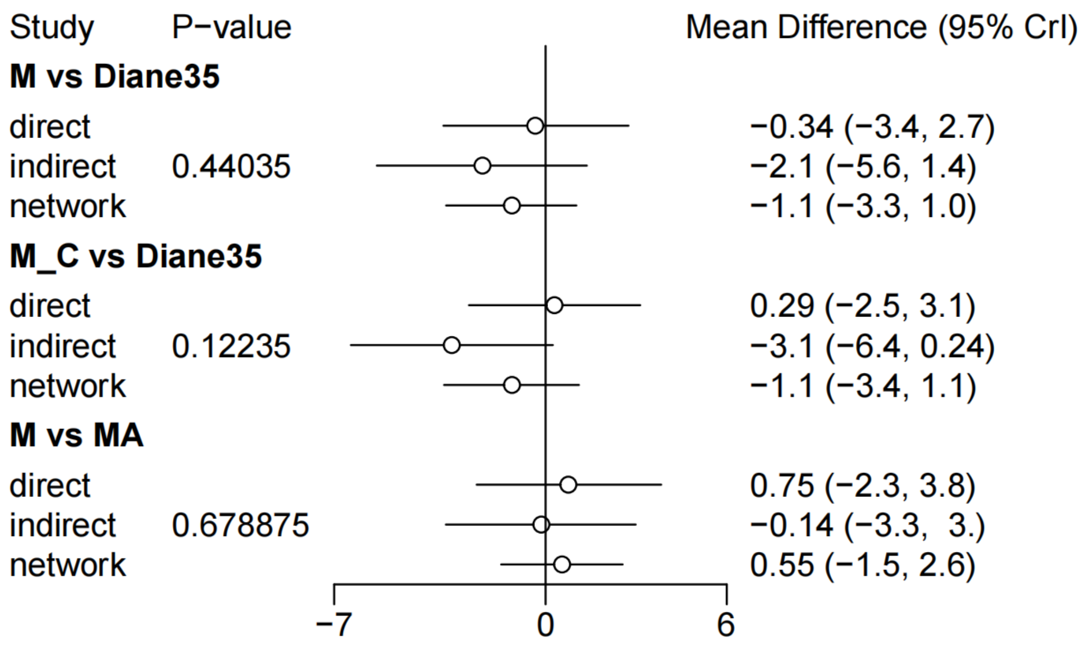
Study
P−value
Mean Difference (95% CrI)
M vs Diane35
direct
−0.34 (−3.4, 2.7)
indirect
0.44035
−2.1 (−5.6, 1.4)
network
−1.1 (−3.3, 1.0)
M_C vs Diane35
direct
0.29 (−2.5, 3.1)
indirect
0.12235
−3.1 (−6.4, 0.24)
network
−1.1 (−3.4, 1.1)
M vs MA
direct
0.75 (−2.3, 3.8)
indirect
0.678875
−0.14 (−3.3, 3.)
network
0.55 (−1.5, 2.6)
−7
0
6

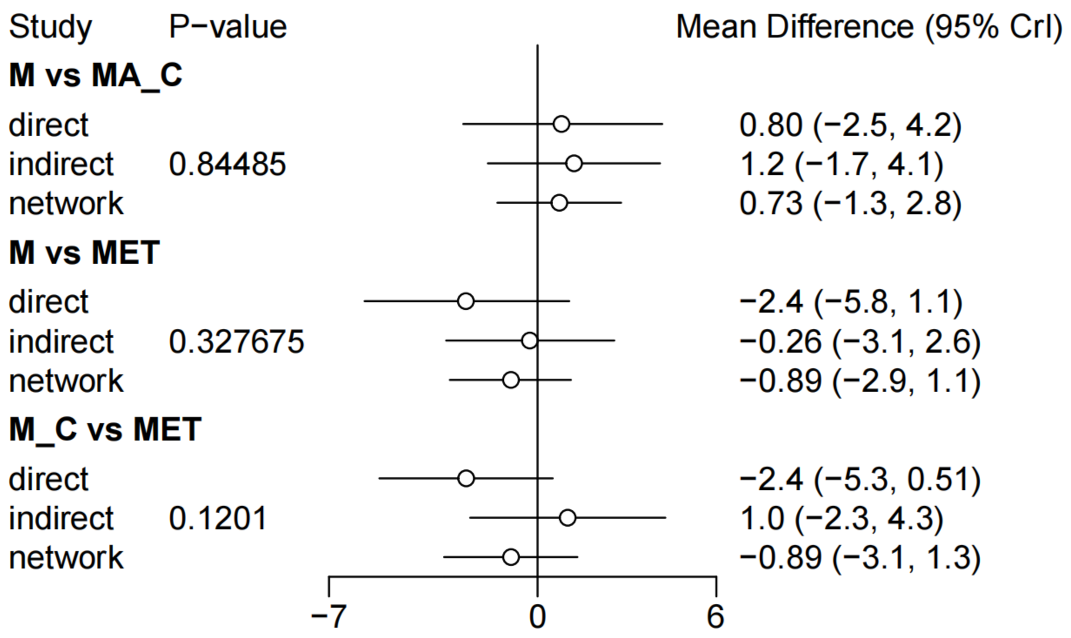

S13-02 The node-splitting analysis of WHR

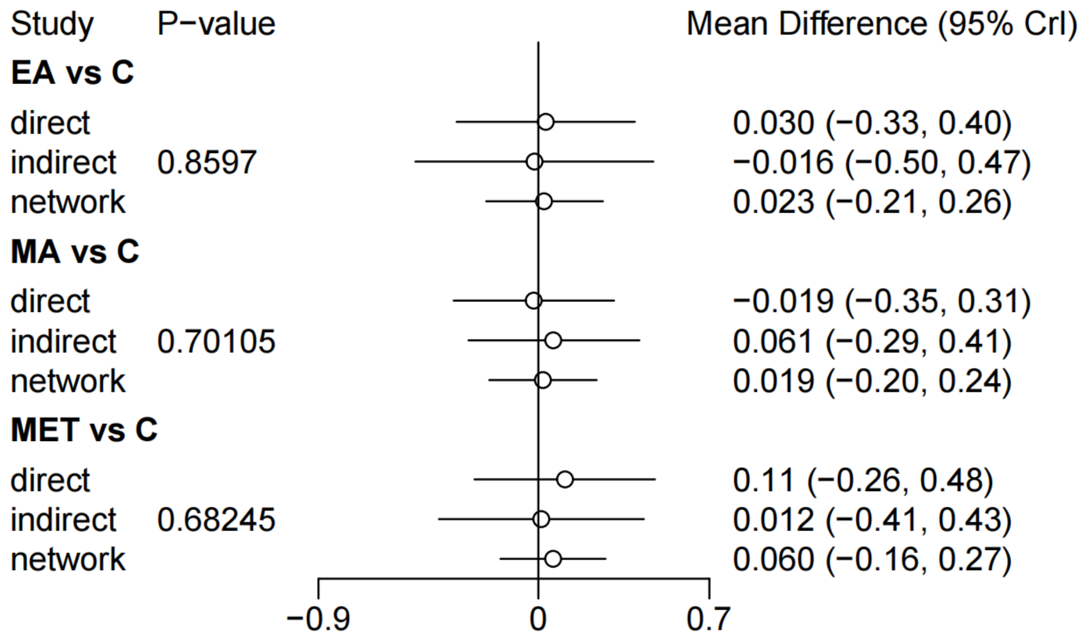

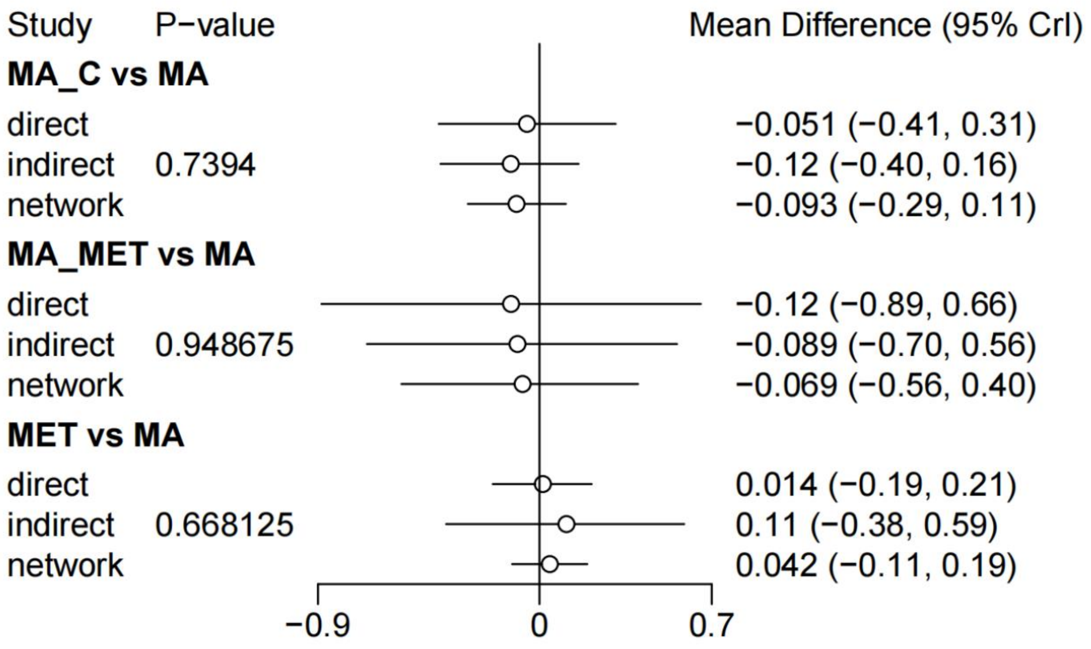

S13-03 The node-splitting analysis of FIN

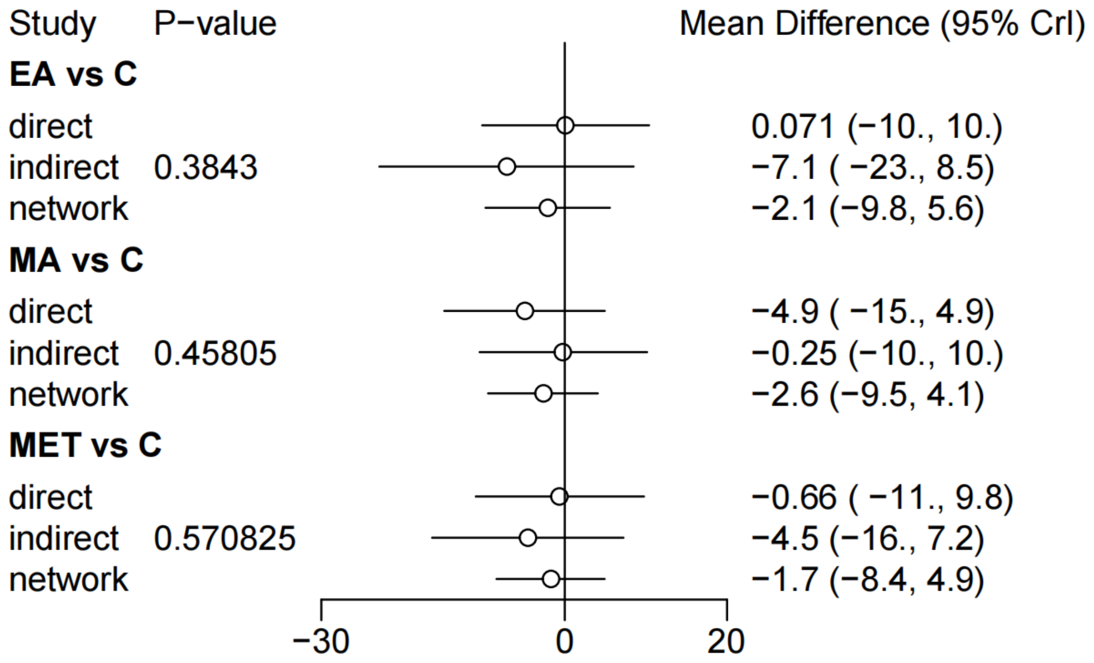

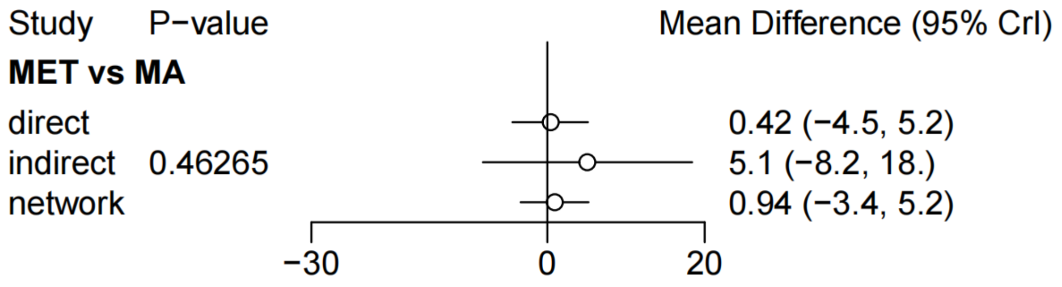

S13-04 The node-splitting analysis of FPG

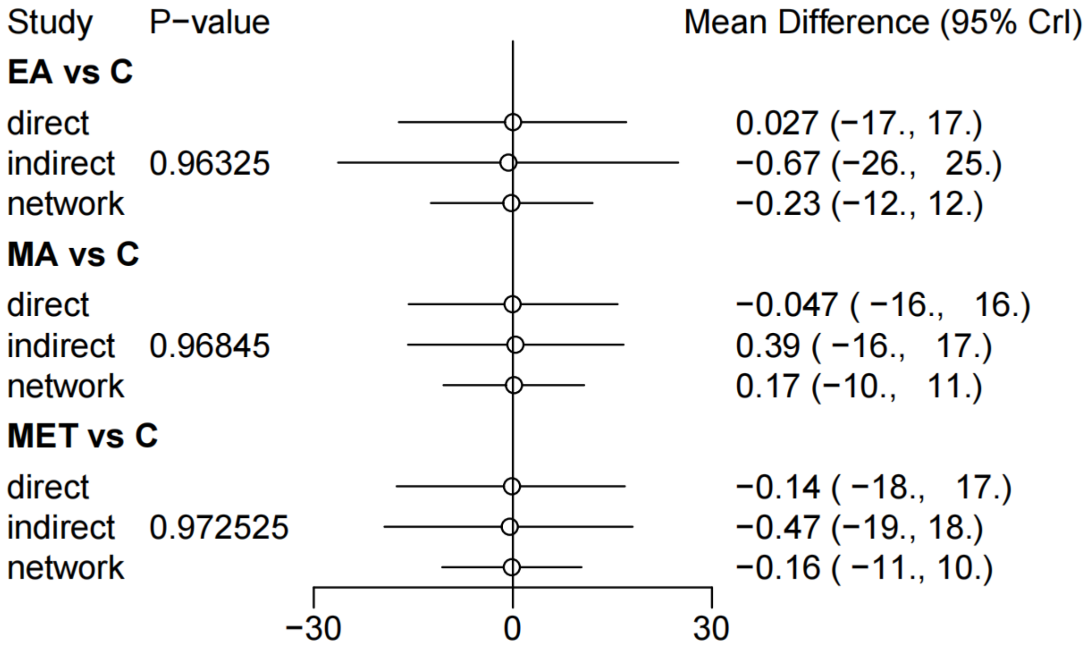

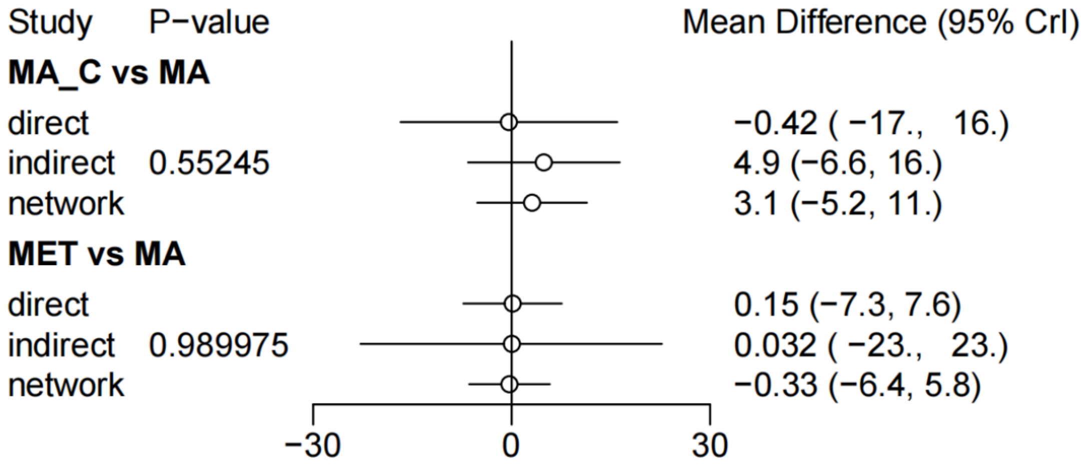

S13-05 The node-splitting analysis of IR

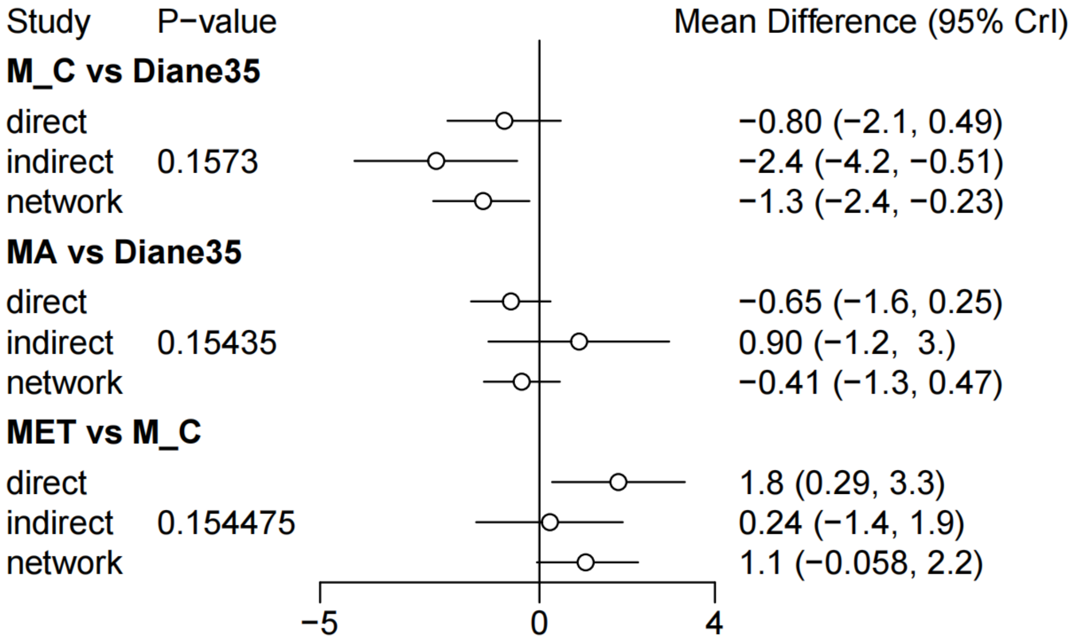

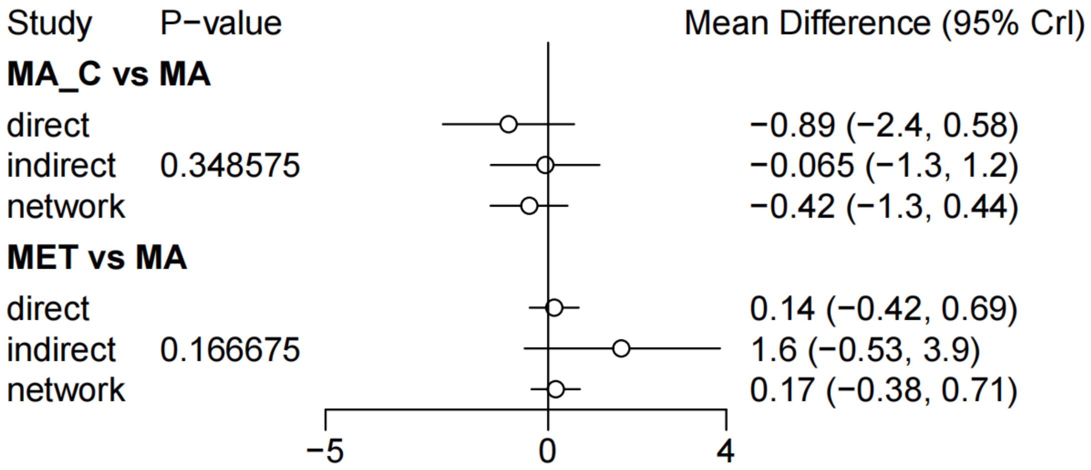

S13-06 The node-splitting analysis of TG

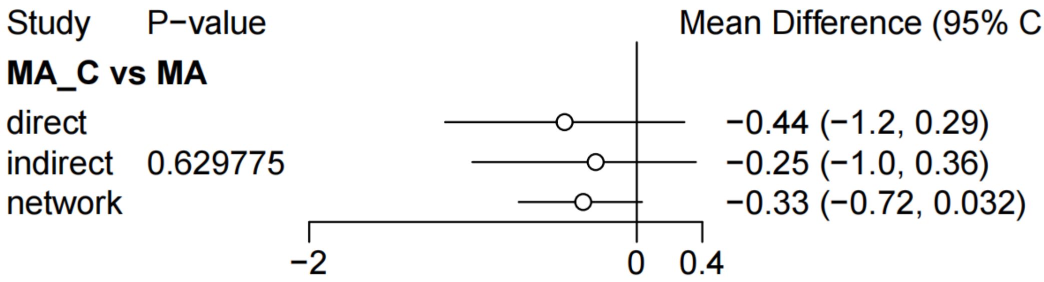

S17. Evidence quality of acupuncture or moxibustion-related therapy

[Acupuncture or moxibustion related therapy] compared to [western medicine or blank control] for [obese PCOS patients]

Bibliography:

Certainty assessment							Summary of findings				
Participants (studies) Follow-up	Risk of bias	Inconsistency	Indirectness	Imprecision	Publication bias	Overall certainty of evidence	Study event rates (%)		Relative effect (95% CI)	Anticipated absolute effects	
							With [对照]	With [干预]		Risk with [对照]	Risk difference with [干预]
**BMI**											
2740 (29 RCTs)	not serious	very serious[a]	not serious	not serious	publication bias strongly suspected[b]	⨁◯◯◯ Very low	1335	1405	-		MD **1.62 lower** (2.05 lower to 1.19 lower)
**WHR**											
1482 (15 RCTs)	not serious	very serious[a]	not serious	not serious	none	⨁⨁◯◯ Low	726	756	-		MD **0.07 lower** (0.11 lower to 0.04 lower)
**FPG**											

[Acupuncture or moxibustion related therapy] compared to [western medicine or blank control] for [obese PCOS patients]

Bibliography:

Certainty assessment							Summary of findings				
1166 (13 RCTs)	not serious	not serious	not serious	not serious	publication bias strongly suspected[b]	⊕⊕⊕◯ Moderate	473	693	-		MD **0.19 lower** (0.28 lower to 0.09 lower)
**FIN**											
1277 (14 RCTs)	not serious	very serious[a]	not serious	not serious	none	⊕⊕◯◯ Low	582	695	-		MD **0.94 lower** (2.23 lower to 0.45 higher)
**IR**											
1310 (14 RCTs)	not serious	very serious[a]	not serious	not serious	none	⊕⊕◯◯ Low	641	669	-		MD **0.18 lower** (0.67 lower to 0.31 higher)
**IR subgroup analysis (simple acupuncture or catgut embedding vs control group)**											
508 (6 RCTs)	serious[d]	not serious	not serious	not serious	none	⊕⊕⊕◯ Moderate	240	268	-		MD **0.03 lower** (0.23 lower to 0.17 higher)
**TG**											

[Acupuncture or moxibustion related therapy] compared to [western medicine or blank control] for [obese PCOS patients]

Bibliography:

Certainty assessment							Summary of findings				
711 (8 RCTs)	serious[d]	not serious	not serious	not serious	none	⊕⊕⊕◯ Moderate	326	385	-		MD **0.35 lower** (0.4 lower to 0.29 lower)

TC

684 (7 RCTs)	serious[d]	not serious	not serious	not serious	none	⊕⊕⊕◯ Moderate	317	367	-		MD **0.36 lower** (0.49 lower to 0.23 lower)

HDL

593 (6 RCTs)	serious[d]	not serious	not serious	not serious	none	⊕⊕⊕◯ Moderate	256	337	-		MD **0.13 higher** (0.06 higher to 0.2 higher)

LDL

503 (5 RCTs)	serious[d]	not serious	not serious	not serious	none	⊕⊕⊕◯ Moderate	226	277	-		MD **0.2 lower** (0.4 lower to 0.01 lower)

W

[Acupuncture or moxibustion related therapy] compared to [western medicine or blank control] for [obese PCOS patients]

Bibliography:

Certainty assessment							Summary of findings				
242 (4 RCTs)	not serious	not serious	not serious	serious[e]	none	⊕⊕⊕◯ Moderate	114	128	-		MD **2.34 lower** (4.51 lower to 0.17 lower)
**WC**											
182 (3 RCTs)	not serious	serious[c]	not serious	serious[e]	none	⊕⊕◯◯ Low	84	98	-		MD **3.13 lower** (4.61 lower to 1.64 lower)

CI: confidence interval; **MD:** mean difference

Explanations

a. Confidence intervals with minimal overlap and tests for heterogeneity highly significant

b. The funnel plot of this intervention is asymmetric.

c. Confidence intervals with minimal overlap and tests for heterogeneity significant

d. The proportion of unclear or high-risk studies was relatively high.

e. The number of the included studies is insufficient.
